# Aryl-Substituted Acridine Donor Derivatives Modulate the Transition Dipole Moment Orientation and Exciton Harvesting Properties of Donor–Acceptor TADF Emitters

**DOI:** 10.1021/acs.jpcc.4c03344

**Published:** 2024-08-20

**Authors:** Ettore Crovini, Kleitos Stavrou, Prakhar Sahay, Bình Minh Nguyễn, Thomas Comerford, Stuart Warriner, Wolfgang Brütting, Andrew Monkman, Eli Zysman-Colman

**Affiliations:** † Organic Semiconductor Centre, EaStCHEM School of Chemistry, University of St Andrews, St Andrews, Fife KY16 9ST, U.K.; ‡ OEM group, Department of Physics, Durham University, South Road, Durham DH1 3LE, U.K.; § Experimental Physics IV, Institute of Physics, University of Augsburg, Augsburg 86159, Germany; ∥ School of Chemistry, University of Leeds, Woodhouse Lane, Leeds LS2 9JT, U.K.

## Abstract

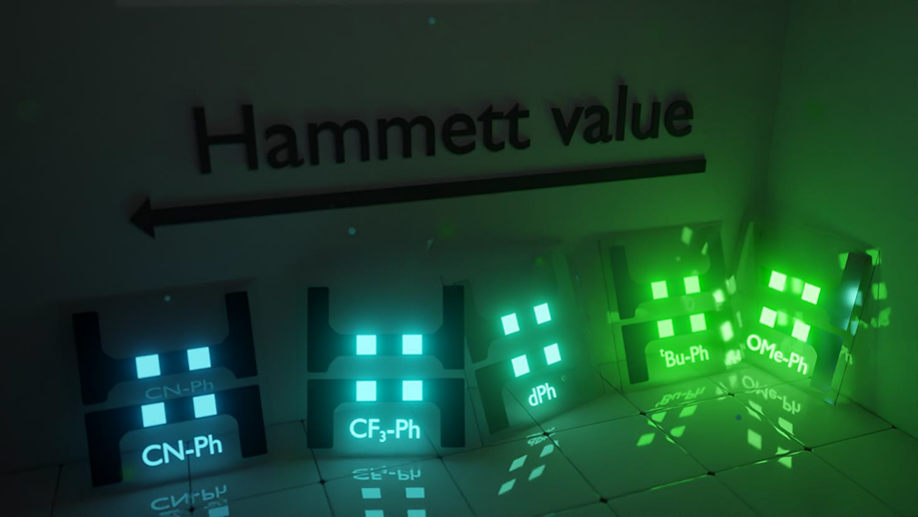

Thermally activated delayed fluorescence (TADF) compounds are highly attractive as sensitizing and emitting materials for organic light-emitting diodes (OLEDs). The efficiency of the OLED depends on multiple parameters, most of which rely on the properties of the emitter including those that govern the internal quantum and outcoupling efficiencies. Herein, we investigate a series of aryl substituted acridine donor derivatives of the donor–acceptor TADF emitter **DMAC-TRZ**, with the objective of correlating their properties, such as triplet harvesting efficiency and transition dipole moment orientation, with their corresponding device efficiency. The decoration of the DMAC donor with substituted aryl groups not only modifies the molecular weight and length of the emitter but also affects the emission color and the capacity for the emitters to efficiently harvest triplet excitons. The presence of electron-withdrawing 4-cyanophenyl and 4-trifluoromethylphenyl groups in, respectively, **CNPh-DMAC-TRZ** and **CF3Ph-DMAC-TRZ**, blue-shifts the emission spectrum but slows down the reverse intersystem crossing rate constant (*k*_RISC_), while the opposite occurs in the presence of electron-donating groups in **^*t*^BuPh-DMAC-TRZ** and **OMePh-DMAC-TRZ** (red-shifted emission spectrum and faster *k*_RISC_). In contrast to our expectations, the OLED performance of the five DMAC-TRZ derivatives does not scale with their degree of horizontal emitter orientation but follows the *k*_RISC_ rates. This, in turn, demonstrates that triplet harvesting (and not horizontal emitter orientation) is the dominant effect for device efficiency using this family of emitters. Nonetheless, highly efficient OLEDs were fabricated with **^*t*^BuPh-DMAC-TRZ** and **OMePh-DMAC-TRZ** as emitters, with improved EQE_max_ (∼28%) compared to the reference **DMAC-TRZ** devices.

## Introduction

Organic light-emitting diodes (OLEDs) are steadily becoming the dominant display technology across a range of consumer electronic markets, such as smart watches, mobile phones, and televisions. This is thanks to their excellent power efficiency, that they are light in weight, they can produce both saturated colors necessary for ultrahigh definition displays as well as true black, and they can be fabricated on a panoply of substrates, including those that are flexible and transparent.^1,2^ OLEDs are based on a multilayer structure of organic semiconductors sandwiched between two electrodes, where the light-emission results from the electroluminescence of the emitter. The application of current to the device injects electrons and generates holes, which migrate through the device via a so-called hopping mechanism. When these recombine, then an exciton is formed, and it is the radiative decay of the exciton that is responsible for the light that ultimately emanates from the OLED. Based on the statistics of spin multiplicity, 25% of the exciton formed by their recombination will have singlet multiplicity, and 75% will have triplet multiplicity. In fluorescent compounds, the singlet exciton can decay radiatively, while the triplet exciton cannot due to the spin-forbidden nature of the T_1_ → S_0_ transition. Both phosphorescent and thermally activated delayed fluorescent (TADF) materials, however, can harvest both singlet and triplet excitons to produce light, but do so via distinct photophysical mechanisms. TADF materials have suitably small energy gaps between the S_1_ and T_1_ excited states (Δ*E*_ST_) that enable triplet excitons to endothermically upconvert to singlets at ambient temperatures through a process called reverse intersystem crossing (RISC). The small Δ*E*_ST_ results from the small exchange integral where the HOMO and LUMO are localized on distinct parts of the emitter molecule. The most widely employed design is based on an emitter with a strongly twisted donor–acceptor (D–A) conformation, where the HOMO will be mainly localized on the donor and the LUMO on the acceptor.

An archetype D–A TADF emitter is **DMAC-TRZ**, first reported by Tsai et al.^3^ This compound has a very small Δ*E*_ST_ of 0.046 eV in 8 wt % doped films in mCPCN resulting from the near orthogonal conformation between the dimethylacridine (DMAC) donor and the triazine (TRZ) acceptor. In this host, **DMAC-TRZ** has a sky-blue emission with a peak photoluminescence wavelength (λ_PL_) of 495 nm and a high photoluminescence quantum yield (Φ_PL_) of 90%. The OLEDs with this emitter showed a maximum external quantum efficiency, EQE_max_, of 26.5% (at 1 cd m^–2^), at a λ_EL_ of 500 nm. **DMAC-TRZ** also exhibits very low concentration quenching, with the neat film of the material having a Φ_PL_ of 83%; the nondoped device showed an EQE_max_ of 20.0%.^3^**DMAC-TRZ** has since been investigated extensively as the emitter in several computational and photophysical studies.^4−6^

**Figure 1:**
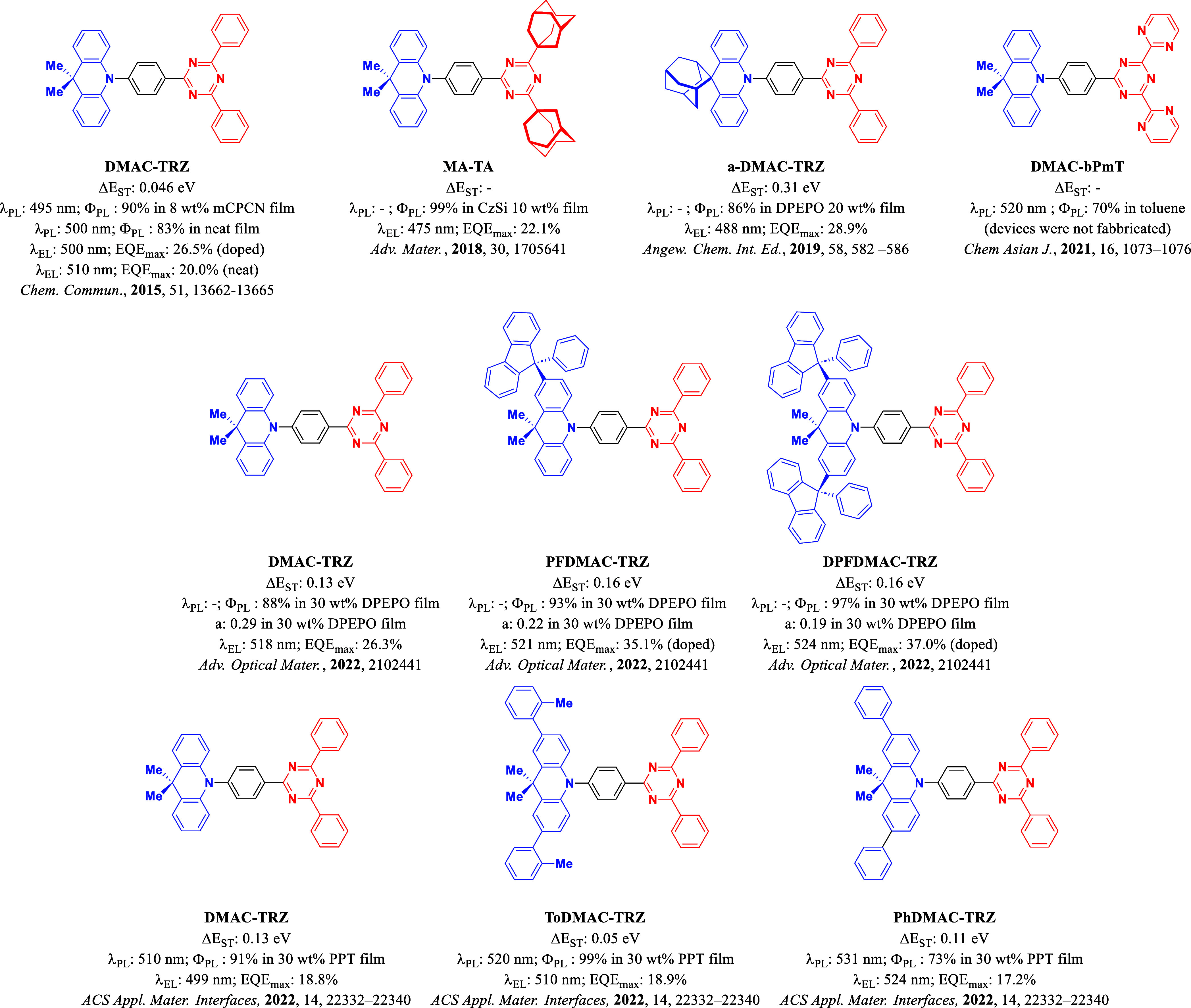
Chemical structure and properties of **DMAC-TRZ** and several reported derivatives. The variation in reported ΔΕ_ST_ values of the doped films of **DMAC-TRZ** originates from a combination of the different doping concentrations, host, and measurement parameters used in each study.

Many have focused on improving further the molecular design of this emitter and have investigated derivatives. Kaji and co-workers reported two modified structures of **DMAC-TRZ** incorporating adamantane at different positions in **MA-TA**,^7^ and **a-DMAC-TRZ**(8) (Figure 1). In the first report, the substitution of the two distal phenyl rings on the triazine acceptor with adamantyl groups reduced the conjugation length of the acceptor, weakening it and leading to a bluer emission. Toluene solutions and neat films of **MA-TA** emit at 469 and 453 nm, respectively, compared to 494 and 500 nm for **DMAC-TRZ**. The 10 wt % doped film of **MA-TA** in CzSi shows a near unity Φ_PL_ of 99%, thus efficient solution-processed blue devices showed an EQE_max_ of 22.1%, at λ_EL_ of 475 nm.^9^ The functionalization of the donor in **a-DMAC-TRZ** increased the rigidity of the structure ultimately leading to a more efficient device than with **DMAC-TRZ**, showing an EQE_max_ of 28.9% and a λ_EL_ of 488 nm.

Kaji and co-workers reported the emitter **DMAC-bPmT**, which contains two distal pyrimidines in lieu of phenyl groups in **DMAC-TRZ**.^10^ The computational study revealed that the presence of these more π-accepting pyrimidines stabilizes the energy of the S_2_ and T_2_ states, bringing them closer in energy to the S_1_ and T_1_ states, which enhanced the reverse intersystem crossing rate constant (*k*_RISC_). Indeed, experimentally *k*_RISC_ is 8.8 × 10^5^ s^–1^, which is three times faster than the *k*_RISC_ of **DMAC-TRZ**, at 2.9 × 10^5^ s^–1^; however, the trade-off is that the Φ_PL_ of **DMAC-bPmT** of 70% is lower (Φ_PL_ of 93% for **DMAC-TRZ**) and the emission in toluene is red-shifted at λ_PL_ of 520 nm (λ_PL_ 500 nm for **DMAC-TRZ**).

Feng et al.^11^ described an arylmethylation strategy to enhance the horizontal orientation of the transition dipole moment (TDM) of **DMAC-TRZ** derivatives in films. **PFDMAC-TRZ** and **DPFDMAC-TRZ** (Figure 1) contain a functionalized DMAC donor containing one and two 9-phenyl-9*H*-fluorene groups, respectively. **PFDMAC-TRZ** and **DPFDMAC-TRZ** have the same Δ*E*_ST_ of 0.16 eV and high Φ_PL_ of 93 and 97%, respectively, in 30 wt % doped films in DPEPO. **DMAC-TRZ**, **PFDMAC-TRZ**, and **DPFDMAC-TRZ** possess anisotropy factors, *a*, of 0.29, 0.22, and 0.19, respectively, and the more horizontal orientation of the latter two was then exploited in the devices, which showed EQE_max_ values of 26.3, 35.1, and 37.0%, respectively.^12^

**ToDMAC-TRZ** and **PhDMAC-TRZ** are examples of two other extended **DMAC-TRZ** derivatives (Figure 1).^13^ The functionalization of **DMAC-TRZ** with either an *ortho*-tolyl unit or a phenyl unit results in a decreased Δ*E*_ST_ from 0.13 eV for **DMAC-TRZ** to 0.05 for **ToDMAC-TRZ** and 0.11 eV for **PhDMAC-TRZ,** in 30 wt % doped films in PPT. Although the Δ*E*_ST_ value of **DMAC-TRZ** here is different from the previously reported one,^3^ is likely a function of a combination of the different host, emitter concentration, and measurement parameters used, in both cases, the value is very small. The λ_PL_ in these doped films is red-shifted from 510 nm for **DMAC-TRZ** to 520 and 531 nm for **ToDMAC-TRZ** and **PhDMAC-TRZ**, respectively. This is due to the strengthening of the donor due to increased conjugation between these aromatic groups and the DMAC donor core. As 30 wt % doped films in PPT, **DMAC-TRZ**, **ToDMAC-TRZ**, and **PhDMAC-TRZ** have Φ_PL_ values of 91, 99, and 73%, respectively. The trend in EQE_max_ of 18.8, 18.9, and 17.2% of the corresponding devices mirrors their Φ_PL_ while their λ_EL_ are blue-shifted at 499, 510, and 524 nm, compared to their λ_PL_.

**Figure 2:**
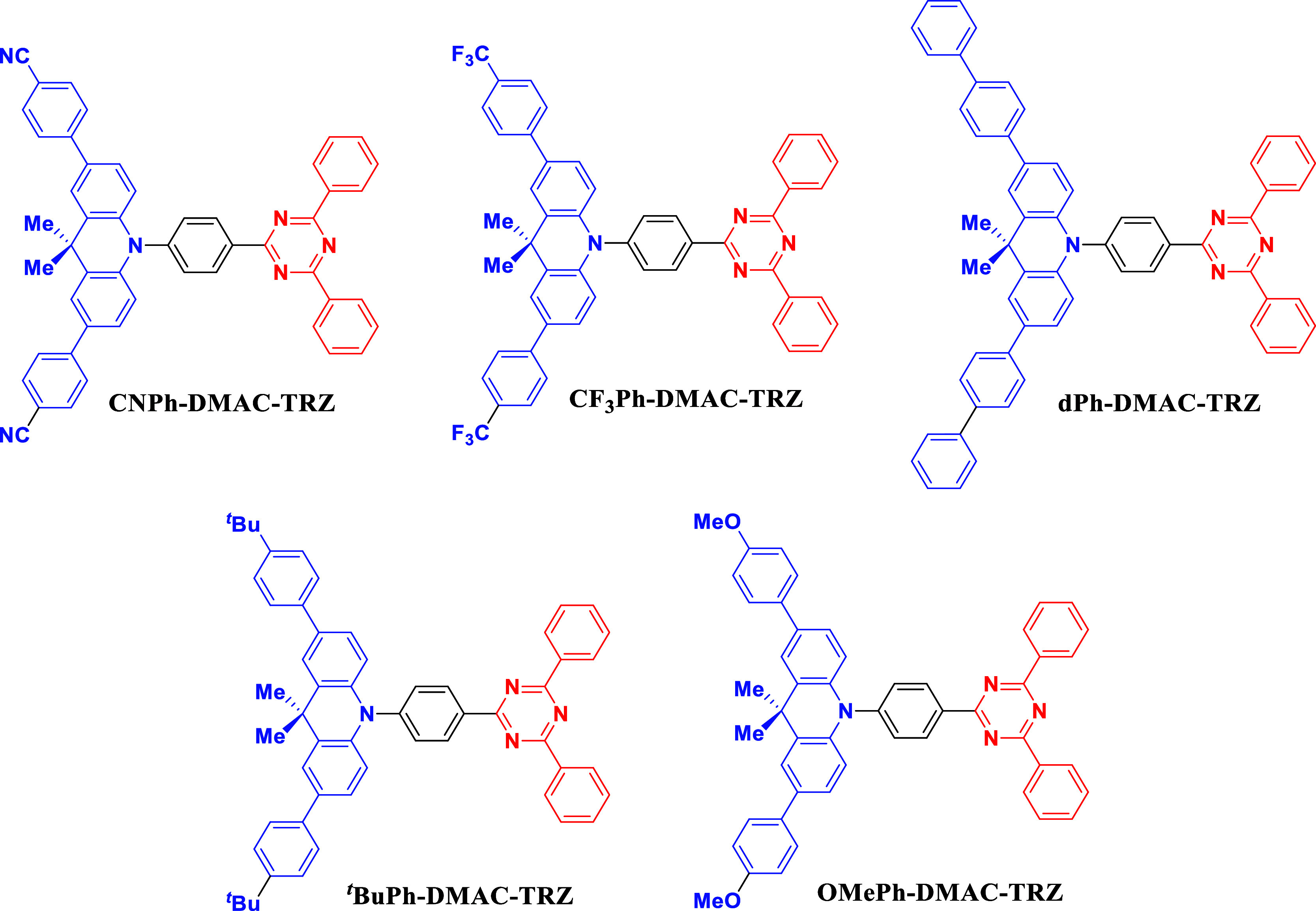
Chemical structures of **CNPh-DMAC-TRZ**, **CF**_**3**_**Ph-DMAC-TRZ**, **dPh-DMAC-TRZ**, ^***t***^**BuPh-DMAC-TRZ**, and **OMePh-DMAC-TRZ**.

Here, we report a rationally designed family of **DMAC-TRZ** derivatives, **CNPh-DMAC-TRZ**, **CF**_**3**_**Ph-DMAC-TRZ**, **dPh-DMAC-TRZ**, ^***t***^**BuPh-DMAC-TRZ, and OMePh-DMAC-TRZ** (Figure 2) wherein the central DMAC donor is decorated with two aryl groups containing at the 4-position differing electron-donating or withdrawing groups. The effect of donor decoration on the color tuning of the emitters, their triplet exciton harvesting, and PL efficiencies is demonstrated. Further, using angle-resolved photoluminescence spectroscopy, we explore how the increased molecular weight and length of the emitter (depending on the nature of the peripheral substituent on the donor) affect the orientation of its TDM. Using optical simulations, the effect of the TDM orientation on the light outcoupling efficiency is explored, revealing that its effect is minor compared to the impact of the RISC rate on the OLED efficiency.

## Methods

### General Synthetic Procedures

The following starting materials were synthesized according to literature protocols, **DMAC**.^14^ All other reagents and solvents were obtained from commercial sources and used as received. Air-sensitive reactions were performed under a nitrogen atmosphere using Schlenk techniques, and no special precautions were taken to exclude air or moisture during workup and crystallization. Flash column chromatography was carried out using silica gel (Silia-P from Silicycle, 60 Å, 40–63 μm). Analytical thin-layer chromatography (TLC) was performed with silica plates with aluminum backings (250 μm with F-254 indicator). TLC visualization was accomplished by a 254/365 nm UV lamp. HPLC analysis was conducted on a Shimadzu LC-40 HPLC system. HPLC traces were performed by using a Shim-pack GIST 3 μm C18 reverse-phase analytical column. GCMS analysis was conducted using a Shimadzu QP2010SE GC-MS instrument equipped with a Shimadzu SH-Rtx-1 column (30 m × 0.25 mm). ^1^H, ^13^C, and ^19^F NMR spectra were recorded on a Bruker Advance spectrometer (500 MHz for ^1^H, 125 MHz for ^13^C, 471 MHz for ^19^F, and 202 MHz for ^31^P). The following abbreviations have been used for multiplicity assignments: “s” for singlet, “d” for doublet, “t” for triplet, “q” for quartet, and “m” for multiplet. ^19^F and ^31^P NMR spectra were recorded with proton decoupling. ^1^H and ^13^C NMR spectra referenced residual solvent peaks with respect to TMS (δ = 0 ppm). Melting points were measured using open-ended capillaries on an Electrothermal 1101D Mel-Temp apparatus and were uncorrected. High-resolution mass spectrometry (HRMS) was performed at the University of Leeds. Atmospheric pressure chemical ionization (APCI) spectra were recorded on a Bruker Impact spectrometer equipped with a direct insertion probe. Elemental analyses were performed at the School of Geosciences at the University of Edinburgh using a Thermo Fisher Scientific Flash SMART 2000 Instrument.

### Electrochemistry Measurements

Cyclic voltammetry (CV) and Differential Pulse Voltammetry analyses were performed on an Electrochemical Analyzer model 620D from CH Instruments. Sample of the different materials were prepared in DCM that was degassed by sparging with DCM-saturated nitrogen gas for 10 min before measurements. All measurements were performed in 0.1 M DCM or a solution of tetrabutylammonium hexafluorophosphate, which was used as the supporting electrolyte. An Ag/Ag^+^ electrode was used as the reference electrode while a glassy carbon electrode and a platinum wire were used as the working electrode and counter electrode, respectively. The redox potentials are reported relative to a saturated calomel electrode (SCE) with a ferrocene/ferrocenium (Fc/Fc^+^) redox couple as the internal standard (0.46 V vs SCE for DCM).^15^ CV was performed with a scan rate of 100 mV/s and a sensitivity of 1 × 10^–5^ A/V. DPV was performed with an increment of a potential of 0.01 V, amplitude of 0.05 V, pulse width of 0.06 s, sample width of 0.02 s, pulse period 0.05 s, quiet time of 2 s, and sensitivity of 1 × 10^–5^ A/V.

### Photophysical Measurements

Optically dilute solutions of concentrations on the order of 10^–5^ M were prepared in HPLC grade methyl-cyclohexane, toluene, DCM, THF, and MeCN for absorption and emission analysis. Absorption spectra were recorded at room temperature on a Shimadzu UV-2600 double-beam spectrophotometer with a 1 cm quartz cuvette. Molar absorptivity determination was verified by a linear least-squares fit of values obtained from at least five independent solutions at varying concentrations ranging from 1 × 10^–5^ to 3.34 × 10^–6^ M.

For emission studies, aerated solutions were bubbled with compressed air for 5 min, and spectra were taken using the same cuvette as for the absorption analysis. Degassed solutions were prepared via three freeze–pump–thaw cycles, and spectra were taken using a homemade Schlenk quartz cuvette. Steady-state emission, excitation spectra, and time-resolved emission spectra were recorded at 298 K using an Edinburgh Instruments F980 and FS5 (the latter for solvatochromism measurements). Samples were excited at 340 nm for steady-state measurements and at 378 nm for time-resolved measurements. Photoluminescence quantum yields for solutions were determined using the optically dilute method^16^ in which four sample solutions with absorbances of ca. 0.09, 0.07, 0.05, and 0.03 at 360 nm were used. The Beer–Lambert law was found to remain linear at the concentrations of the solutions. For each sample, linearity between absorption and emission intensity was verified through linear regression analysis with the Pearson regression factor (*R*^2^) for the linear fit of the data set surpassing 0.9. Individual relative quantum yield values were calculated for each solution, and the values reported represent the slope obtained from the linear fit of these results. The equation Φ_s_ = Φ_r_(*A*_r_/*A*_s_)(*I*_s_/*I*_r_)(*n*_s_/*n*_r_)^2^ was used to calculate the relative quantum yield of the sample, where (Φ_r_) is the absolute quantum yield of the external reference quinine sulfate (Φ_r_ = 54.6% in 1 N H_2_SO_4_).^17^ A stands for the absorbance at the excitation wavelength, *I* is the integrated area under the corrected emission curve, and *n* is the refractive index of the solvent. The subscripts “s” and “r” represent sample and reference, respectively. The experimental uncertainty in the emission quantum yields is conservatively estimated to be 10%, though we have found that statistically, we can reproduce Φ_PL_ values to 3% relative error. Thin-film Φ_PL_ measurements were performed by using an integrating sphere in a Hamamatsu C9920-02 system. A xenon lamp coupled to a monochromator enabled excitation selectivity, chosen here to be 340 nm. The output was then fed into the integrating sphere via a fiber, exciting the sample. PL spectra were collected with a multimode fiber and detected with a back-thinned CCD. Doped thin films were prepared by mixing sample (10 wt %) and host material in Chlorobenzene solution, followed by spin-casting on a quartz substrate. The Φ_PL_ of the films were then measured in air and by purging the integrating sphere with flowing N_2_ gas for 10 min. Time-resolved PL measurements of the solution samples were carried out using the time-correlated single-photon counting technique. The samples were excited at 378 nm by a pulsed laser diode (Picoquant, model PLS 370) and were kept in a vacuum of <8 × 10^–4^ mbar.

Solid-state sample time-resolved measurements were performed using a spectrograph (Horiba Triax) and a Stanford Computer Optics 4 Picos intensified charge-coupled device camera, where samples were excited with a Nd:YAG laser (EKSPLA, 10 Hz, 355 nm) under a vacuum. The singlet–triplet splitting energy, Δ*E*_ST_, was estimated by recording the prompt fluorescence spectra and phosphorescence emission at 77 K.

### Angular-Dependent Photoluminescence Spectroscopy

Thin films of emitter and host were co-evaporated in a high vacuum on a precleaned glass substrate. This substrate was then glued with an index-matching fluid on a fused-silica prism, which was mounted on the rotating stage. The organic film was then irradiated with a UV laser (Kimmon, HeCd laser, λ = 325 nm) under vertical incidence and was rotated from −90° to +90° with respect to the substrate normal. The luminescence was recorded with a grating spectrograph coupled to a liquid-nitrogen-cooled charge-coupled device (Princeton Instruments Acton 2300i with PyLoN detector) in s- and p-polarization mode. p-polarized signal was then subjected to numerical simulation to calculate the anisotropy factor (*a*):
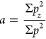
where Σ*p*_*z*_^2^ is the sum of the power emitted by vertically oriented dipoles and Σ*p*^2^ is the sum of the power emitted by all emitting dipoles.^18^ The parameter a (or synonymously Θ_v_ = ⟨cos^2^ ϑ⟩) denotes the second moment of the TDM’s angular distribution around the surface normal of the film, where ϑ is the angle between the molecule’s TDM vector and the said direction. The details about the method can be further found in the references.^18,19^

### OLED Fabrication and Testing

OLED devices of the materials were fabricated on patterned indium tin oxide (ITO)-coated glass (VisionTek Systems) with a sheet resistance of 15 Ω/sq. After sonicating in acetone and isopropanol, oxygen-plasma-cleaned substrates were loaded into a Kurt J. Lesker Super Spectros deposition chamber, and both the small molecule and cathode layers were thermally evaporated at a pressure below 10^–7^ mbar. Devices tested in a calibrated 10 inch integrating sphere (Labsphere) and their electrical properties were measured using a source meter (Keithley 2400). Emission spectra were simultaneously measured using a calibrated fiber-coupled spectrometer (Ocean Optics USB4000). All devices were evaluated at RT (298 K) and under an air atmosphere.

### Theoretical Calculations

Calculations were submitted and processed using the Silico v 0.20.5 software package^20,50^ which incorporates a number of publicly available software libraries, including: cclib^21^ for parsing of result files, VMD^22^/Tachyon^23^ for 3D rendering, Open Babel^24^/Pybel^25^ for file interconversion.

All ground-state optimizations have been carried out using the density functional theory (DFT) level implemented within Gaussian 16^26^ in the gas phase, using the PBE0^27^ functional and the 6-31G(d,p) basis set.^28^ Excited-state calculations were performed using TD-DFT within the Tamm–Dancoff approximation^29^ using the same functional and basis set as for ground-state geometry optimization. This methodology has been demonstrated to show a quantitative estimate of Δ*E*_ST_ in comparison to the experiment.^30^

## Results and Discussion

### Theoretical Calculations

**Figure 3:**
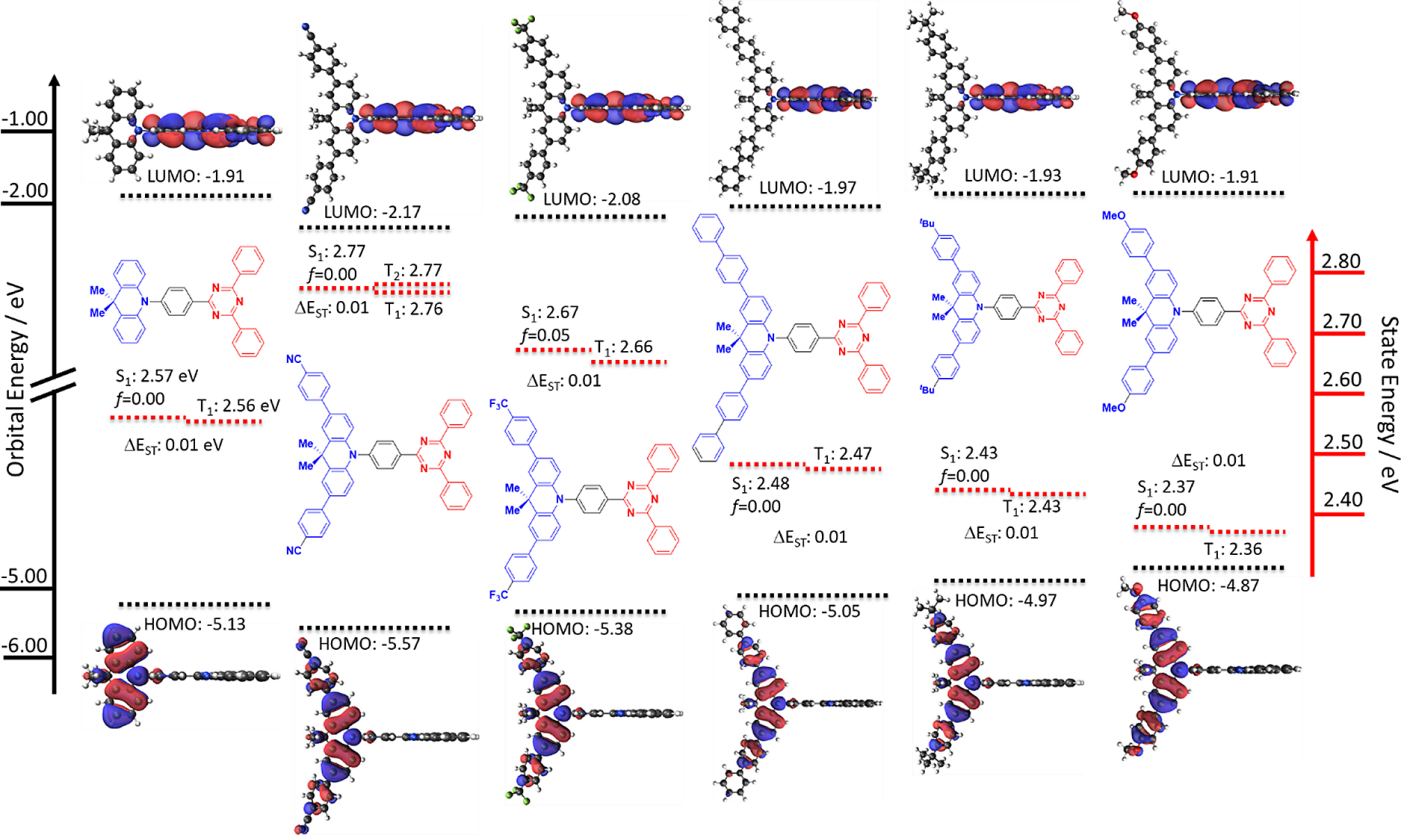
HOMO and LUMO electron density distributions and energy levels, excited-state energy levels of **DMAC-TRZ** and its five derivatives (obtained via DFT and TD-DFT at the PBE0/6-31G(d,p) level, isovalues: MO = 0.02, density = 0.0004).

The optoelectronic properties of **DMAC-TRZ** and the five new derivatives were first assessed using a combined DFT and TD-DFT theoretical study at the PBE0/6-031G(d,p)^27,28^ level (Figure 3). **DMAC-TRZ** has HOMO and LUMO energy levels of −5.13 and −1.91 eV, respectively. The DMAC is twisted to almost 90° with respect to the triazine, as previously observed in the literature,^3^ leading to a very small overlap between the HOMO located on the DMAC and the LUMO located on the triazine, and thus an almost zero Δ*E*_ST_; S_1_ and T_1_ levels are 2.57 and 2.56 eV, respectively. The small overlap between HOMO and LUMO also leads to an oscillator strength with a value of zero.

The ground-state optimized structures of the five derivatives all possess similar conformations as that of **DMAC-TRZ**, thus leading to Δ*E*_ST_ values of ca. 0.01 eV. As the substituents at the 4-position of the aryl groups change from electron-withdrawing (CN and CF_3_) to electron-donating (^*t*^Bu and OMe) the HOMO energy level becomes progressively destabilized, with values of −5.57, −5.38, −5.05, −4.97, and −4.87 eV for **CNPh-DMAC-TRZ**, **CF**_**3**_**Ph-DMAC-TRZ**, **dPh-DMAC-TRZ**, ^*t*^**BuPh-DMAC-TRZ**, and **OMePh-DMAC-TRZ**, respectively. The LUMO levels are likewise destabilized along the series, although to a lesser extent than the HOMO levels as the triazine is only weakly conjugated to the DMAC-based donor. The LUMO values are −2.17, −2.08, −1.97, −1.93, and −1.91 eV for **CNPh-DMAC-TRZ**, **CF**_**3**_**Ph-DMAC-TRZ**, **dPh-DMAC-TRZ**, ^***t***^**BuPh-DMAC-TRZ**, and **OMePh-DMAC-TRZ**, respectively. The HOMO level of **DMAC-TRZ** (−5.13 eV) falls in the middle of the series between those of **CF**_**3**_**Ph-DMAC-TRZ** and **dPh-DMAC-TRZ**. The LUMO of **DMAC-TRZ** (−1.91 eV) falls between those of **dPh-DMAC-TRZ** and ^***t***^**BuPh-DMAC-TRZ**. The biphenyl group is an inductively electron-withdrawing moiety, which can then slightly stabilize the LUMO level, making it lower in energy than **DMAC-TRZ**.

The singlet and triplet energy levels are also affected by the nature of the substituent on the DMAC donor. The energies of the excited states become systematically more stable across the series (Figure 3). The S_1_/T_1_ energies are 2.77/2.76, 2.67/2.66, 2.48/2.47, 2.37/2.36, and 2.43/2.43 eV for **CNPh-DMAC-TRZ**, **CF**_**3**_**Ph-DMAC-TRZ**, **dPh-DMAC-TRZ**, ^***t***^**BuPh-DMAC-TRZ**, and **OMePh-DMAC-TRZ**, respectively. The S_1_/T_1_ energies of **DMAC-TRZ** lie between those of **CF**_**3**_**Ph-DMAC-TRZ** and **dPh-DMAC-TRZ**. All of the compounds possess S_1_ and T_1_ states that possess charge transfer (CT) character (HOMO → LUMO transition). **dCNPh-DMAC-TRZ** is the only molecule that has a degenerate T_2_ state, which has a locally excited (LE) character localized on the donor moiety (HOMO → LUMO+2 transition). The ca. 90° conformation and very small Δ*E*_ST_ values of ca. 0.01 eV also lead to an effective oscillator strength value of 0.00. The only exception is observed in **CF**_**3**_**Ph-DMAC-TRZ** with a *f* of 0.05.

### Synthesis

**Scheme 1:**
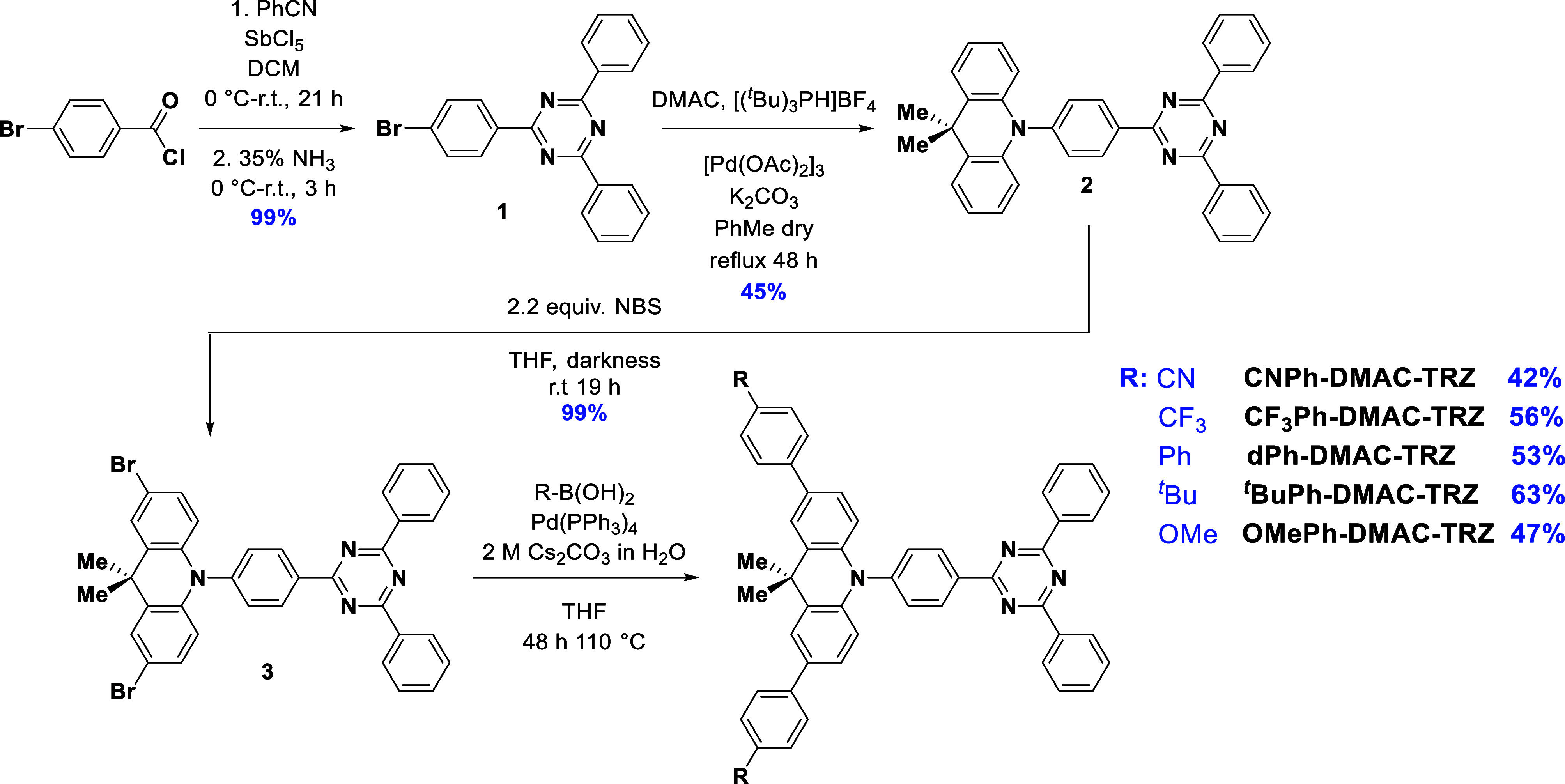
Synthetic Procedure for the Five **DMAC-TRZ** Derivatives

The five **DMAC-TRZ** derivatives were synthesized via a four-step synthetic procedure (Scheme 1). Bromotriazine (**1**) was obtained in quantitative yield following a Lewis acid-catalyzed cyclization of 4-bromobenzoyl chloride and benzonitrile using antimony pentachloride in DCM, followed by the addition of 35% ammonia.^31^ Triazine is then coupled to DMAC through a Buchwald–Hartwig coupling in 45% yield.^3^**DMAC-TRZ** (**2**) was then quantitatively dibrominated using 2.2 equiv of NBS in THF, shielded from light.^32^ Finally, the DMAC-TRZ derivatives were accessed via a Suzuki–Miyaura cross-coupling of **3** with the corresponding arylboronic acid; the reaction was carried out in a pressure vessel at 110 °C for 48 h to improve the yield of the coupled products. Yields for the final step ranged between c.a. 42 and 63%. The identity and purity of the compounds were confirmed by combination ^1^H, ^13^C, and ^19^F NMR spectroscopy, Mp determination, HRMS, HPLC, and EA.

### Optoelectronics Properties

**Figure 4:**
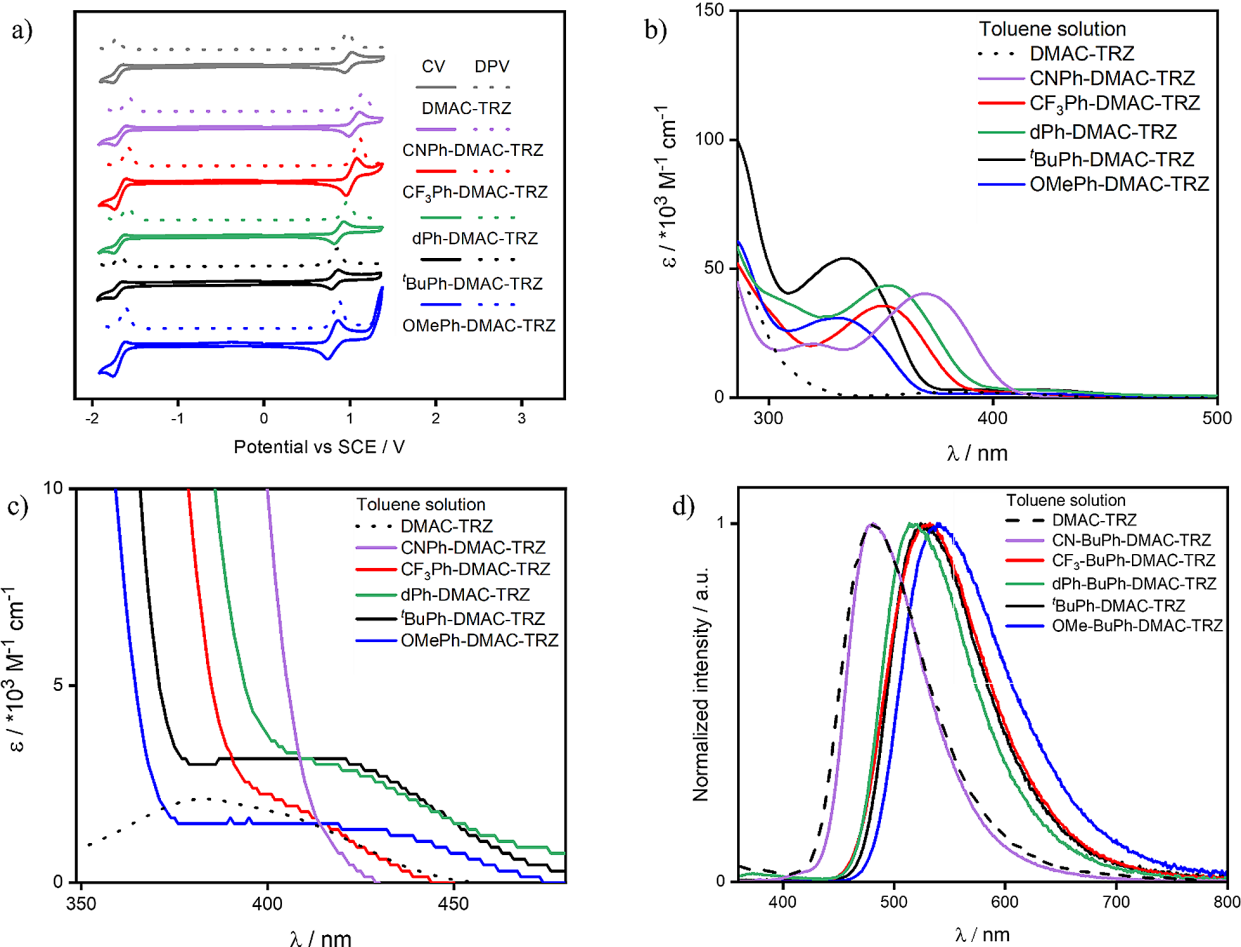
(a) Cyclic voltammetry (CV) and differential pulse voltammetry (DPV) of **DMAC-TRZ** and derivatives in DCM (scan rate = 100 mV/s); (b) UV–vis absorption spectra of **DMAC-TRZ**, and derivatives in toluene and (c) zoom on the low intensity CT bands; (d) photoluminescence spectra of **DMAC-TRZ** and its derivatives in toluene (λ_exc_ = 340 nm, 10^–5^ M solution).

CV and DPV of **DMAC-TRZ** (for comparison) and the five emitters (Figure 4a) were carried out in DCM. The electrochemistry of **DMAC-TRZ** (oxidation attributed to the DMAC and reduction attributed to the triazine) matches the previously reported data,^3,33,34^ with oxidation and reduction potentials at *E*_ox_ of 0.97 V and *E*_red_ −1.72 V, respectively (taken from DPV) versus SCE. The corresponding HOMO and LUMO energy values are −5.31 and −2.62 V.

Despite the predicted small changes in LUMO energies, there is no significant change in the reduction potentials, with values of −1.68, −1.68, −1.69, −1.71, and −1.69 V for **CNPh-DMAC-TRZ**, **CF**_**3**_**Ph-DMAC-TRZ**, **dPh-DMAC-TRZ**, ^***t***^**BuPh-DMAC-TRZ**, and **OMePh-DMAC-TRZ**, respectively, leading to LUMO values of around ∼ −2.65 eV, comparable to that of **DMAC-TRZ** (−2.62 eV). The oxidation potentials follow the trend observed for the DFT calculations, with electron-donating and conjugating groups cathodically shifting the oxidation potential, with values of 1.04, 1.01, 0.87, 0.81, and 0.79 V for **CNPh-DMAC-TRZ**, **CF**_**3**_**Ph-DMAC-TRZ**, **dPh-DMAC-TRZ**, ^*t*^**BuPh-DMAC-TRZ**, and **OMePh-DMAC-TRZ**, respectively. The HOMO energy levels values, obtained from these measured oxidation potentials, are −5.38, −5.35, −5.21, −5.15, and −5.13 eV for **CNPh-DMAC-TRZ**, **CF**_**3**_**Ph-DMAC-TRZ**, **dPh-DMAC-TRZ**, ^*t*^**BuPh-DMAC-TRZ**, and **OMePh-DMAC-TRZ**, respectively, while **DMAC-TRZ** falls in the middle of the series (−5.31 eV).

The UV–vis absorption of **DMAC-TRZ** and its five derivatives were carried out in a toluene solution (Figure 4b,c). The very low intensity of the lowest energy absorption band of **DMAC-TRZ** (∼400 nm) is reflective of the orthogonal conformation between donor and acceptor. In this conformation, the poor overlap between HOMO and LUMO results in an oscillator strength of almost zero, reflected in the very low molar absorptivity for this CT transition (ε = 2000 M^–1^ cm^–1^).

The absorption spectra of the five derivatives reveal the first instance of a structure–property trend that will be a staple for the series: ^***t***^**BuPh-DMAC-TRZ** and **OMePh-DMAC-TRZ** have similar behavior, as do **CF**_**3**_**Ph-DMAC-TRZ**, and **dPh-DMAC-TRZ**, while the behavior of **CNPh-DMAC-TRZ** is rather distinct. Distinct from the absorption spectrum of **DMAC-TRZ**, there is a well-defined low-energy band (ε > 10^4^ M^–1^ cm^–1^) in the absorption spectra of all five emitters (Figure 4b). This band is assigned to a π–π* transition localized on the aryl-substituted DMAC donor, verified by TD-DFT calculations (Table S2).^10^ The donor–acceptor CT absorption bands of the five derivatives, characterized by their low energy and small extinction coefficient, are shown in Figure 4c. The CT absorption band falls around 410 nm for ^***t***^**BuPh-DMAC-TRZ**, **dPh-DMAC-TRZ,** and **OMePh-DMAC-TRZ**, with ε values of 3050, 3040, and 1190 M^–1^ cm^–1^, respectively. The CT band is slightly blue-shifted in **CF**_**3**_**Ph-DMAC-TRZ** at around 400 nm (ε = 2170 M^–1^ cm^–1^) and is of a similar profile to that of the highly twisted conformer of **DMAC-TRZ**. **CNPh-DMAC-TRZ** has two absorption bands, at 369 and 318 nm, assigned by TDDFT calculations (Figure S35) to different^1^ LE transitions on the acridine donor to the S_3_, and S_5_ states, respectively, with ε values of 40,150 M^–1^ cm^–1^ (at 369 nm) and 20,330 M^–1^ cm^–1^ (at 318 nm). The red-shifted, strong absorption bands of the CNPh-acridine donor obscure the weak ^1^CT absorption.

The absorption and emission spectra of **DMAC-TRZ** and its five derivatives were measured in different polarity solvents (Figure S36). The absorption spectra are mostly unaffected by the solvent polarity, presumably because of the low ground-state dipole moment of these compounds, while the emission spectra follow the expected bathochromic shift with increasing solvent polarity, which is associated with excited states of CT character. Indeed, toluene, THF, and DCM solutions show broad and unstructured spectra, typical of a CT-type emission, while methyl-cyclohexane shows a narrower and more structured LE-type emission, in all cases.

The photophysics of the emitters were then investigated in a degassed toluene solution (Figure S37). **DMAC-TRZ** in toluene emits at 500 nm. **CNPh-DMAC-TRZ**, and **CF**_**3**_**Ph-DMAC-TRZ** present blue-shifted emission (λ_PL_ of 484 and 487 nm, respectively), while the other derivatives emission is red-shifted (λ_PL_ of 511, 518, and 528 nm for **dPh-DMAC-TRZ**, ^***t***^**BuPh-DMAC-TRZ**, and **OMePh-DMAC-TRZ**, respectively), compared to **DMAC-TRZ** (Figure 4d). **DMAC-TRZ** in degassed toluene solution has a Φ_PL_ of 67%, which decreases to 21% when it is exposed to air. This is lower than the literature-reported value (Φ_PL_ ∼ 93%), presumably due to different excitation wavelength used and measurement methods^10^**CNPh-DMAC-TRZ**, **CF**_**3**_**Ph-DMAC-TRZ**, **dPh-DMAC-TRZ**, ^***t***^**BuPh-DMAC-TRZ**, **OMePh-DMAC-TRZ** have Φ_PL_ values (degassed/aerated) of 41/32, 72/28, 70/35, 74/24, and 75/23%, respectively. Thus, all compounds have larger Φ_PL_ values compared to the parent emitter, apart from **CNPh-DMAC-TRZ**, which also shows less sensitivity to oxygen quenching. To explore the latter, and more specifically the contribution of the delayed component, time-resolved photoluminescence measurements (TRPL) were carried out in both the degassed and aerated toluene solutions.

The TRPL of the degassed toluene solution of **DMAC-TRZ** possesses both prompt and delayed components with monoexponential decays and lifetimes, τ_p_, of 20.8 ns and τ_d_, of 5.21 μs, respectively (Figure S37). These lifetimes are comparable to those previously reported in the literature for the same compound.^5^ As expected, the delayed component disappears after the solution is exposed to air. The derivatives decorated with electron-donating groups have slightly longer τ_p_ than **DMAC-TRZ**, with monoexponential decays of 28.70 and 28.09 ns for ^***t***^**BuPh-DMAC-TRZ** and **OMePh-DMAC-TRZ**, respectively (Figure S37). Their degassed solutions possess much shorter delayed lifetimes than **DMAC-TRZ**, with monoexponential τ_d_ of 2.39 and 1.49 μs, for ^***t***^**BuPh-DMAC-TRZ** and **OMePh-DMAC-TRZ**, respectively, each of which disappears when the solution is exposed to air. This implies a more efficient reverse intersystem crossing rate (*k*_RISC_) for these two emitters, as both the amplitude and the lifetime of the delayed component improve. The TRPL of **CF**_**3**_**Ph-DMAC-TRZ** and **dPh-DMAC-TRZ** appears to behave in the opposite manner with shorter τ_p_ of 10.9 and 15.4 ns, respectively (Figure S37) and longer τ_d_ and biexponential decay kinetics (average τ_d_ of 20.7 and 25.8 μs, respectively), which also disappear after exposure to air. There is no delayed emission observed for **CNPh-DMAC-TRZ** and the τ_p_ is 8.24 ns, which decreases to 6.53 ns when exposed to air due to a small degree of singlet quenching caused by the presence of oxygen.^35^ The short prompt lifetime is consistent with emission from an LE state.

The photophysical properties of the materials in the solid state were first studied in the host matrix mCP (Figure S38). This host was chosen as it has a relatively high triplet energy (2.9 eV), which makes it suitable for both green and blue devices.^36^ The Φ_PL_ of ^***t***^**BuPh-DMAC-TRZ** appears to decrease slightly at concentrations above 10 wt % (Table S7). Thus, 10 wt % loading was chosen for all the emitters in mCP.^5^ The trend in the solid-state PL spectra of the six emitters at 10 wt % in mCP (Figure S38a) matches that observed in toluene (Figure 4d). Interestingly, the λ_PL_ values of **DMAC-TRZ**, **CNPh-DMAC-TRZ**, and **CF**_**3**_**Ph-DMAC-TRZ** are almost isoenergetic in mCP (Table S6). This is attributed to the stronger intermolecular interactions taking place in the films.^37,38^

The TRPL behavior in the mCP films is also comparable to that observed in toluene; notably, there is a small degree of delayed emission observed for **CNPh-DMAC-TRZ** (Figure S38). The presence of electron-donating substituents on the donor leads to a longer-lived prompt fluorescence, while the opposite trend is observed for the delayed fluorescence lifetimes. The TADF character of all of the materials was confirmed by temperature-dependent TRPL measurements, where the contribution from the delayed emission increases with increasing temperature (Figure S38). The delayed emission of both ^***t***^**BuPh-DMAC-TRZ** and **OMePh-DMAC-TRZ** is only modestly quenched at 80 K, attributed to the highly efficient RISC process in these two emitters; thus, at this temperature, triplet harvesting is not fully suppressed.^39^ This effect is also observed for their 80 K phosphorescence spectra, which are not structured, resulting in a misleading triplet energy value, while their 20 K phosphorescence spectra are more structured (indicating mixed CT/LE character) and the triplet energy value appears similar in both molecules, around 2.65 eV (Figure S39). This results in a Δ*E*_ST_ of 30 meV for both emitters and is in good agreement with the DFT calculations (Figure 3). For **CNPh-DMAC-TRZ**, **CF**_**3**_**Ph-DMAC-TRZ**, and **dPh-DMAC-TRZ**, 80 K is a sufficiently low temperature to effectively suppress TADF, and structured phosphorescence is observed with energy onset at 2.5, 2.63, and 2.54 eV, respectively (Figure S39). The resulting Δ*E*_ST_ values are 290, 210, and 150 meV for **CNPh-DMAC-TRZ**, **CF**_**3**_**Ph-DMAC-TRZ**, and **dPh-DMAC-TRZ**, respectively. From the modeled lowest triplet value of the aryl-decorated donor units (Figure S40), **CNPh-DMAC**, **CF**_**3**_**Ph-DMAC**, and **dPh-DMAC** have more stabilized T_1_ states compared to ^*t*^**BuPh-DMAC** and **OMePh-DMAC**, these trend in a similar manner to the experimentally obtained values. Consequently, we propose that in **CNPh-DMAC-TRZ**, **CF**_**3**_**Ph-DMAC-TRZ**, and **dPh-DMAC-TRZ** the T_1_ state is localized on the aryl-decorated acridine donor, while in ^*t*^**BuPh-DMAC-TRZ** and **OMePh-DMAC-TRZ,** the T_1_ state has a mixed CT/LE character.

The Φ_PL_ values of the six emitters in 10 wt % doped films in mCP are compiled in Table S6 In all cases, the Φ_PL_ of the mCP doped films is lower than that in toluene. It has already been established that the Φ_PL_ of **DMAC-TRZ** is higher in higher dipole moment matrices.^3^ For these reasons, screening of the Φ_PL_ in high triplet energy hosts mCP, mCBPCN, and DPEPO host was conducted using ^***t***^**BuPh-DMAC-TRZ** as a representative example (Table S7). As well, mCBPCN and DPEPO have relatively high MW and *T*_g_, which would be beneficial to influence the horizontal orientation of the TDM of these materials.^12^ At concentrations below 10 wt % the Φ_PL_ values are similar in the three hosts because the measurement error is higher.^5^ At higher concentrations, the Φ_PL_ values in mCBPCN and DPEPO are similar and higher than those in mCP. Thus, mCBPCN was chosen as the host material to carry on the solid-state photophysical analysis, as DPEPO is recognized to be unstable in the OLEDs and this would thus negatively impact the device lifetime.^40^ Furthermore, 10 wt % was chosen as the doping concentration as at this concentration there are few intermolecular interactions as already observed in films of **DMAC-TRZ**.^5,41^ The Φ_PL_ values in 10 wt % mCBPCN are 87, 34, 70, 70, 72, and 72% for **DMAC-TRZ, CNPh-DMAC-TRZ**, **CF**_**3**_**Ph-DMAC-TRZ**, **dPh-DMAC-TRZ**, ^***t***^**BuPh-DMAC-TRZ**, and **OMePh-DMAC-TRZ**, respectively (Table S6).

**Figure 5:**
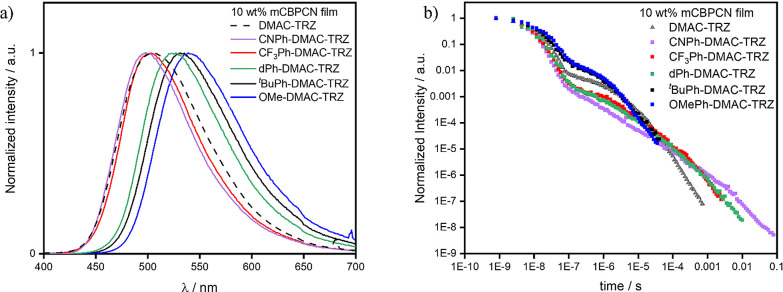
(a) Photoluminescence spectra and (b) time-resolved emission decays at room temperature of **DMAC-TRZ** and its five derivatives in 10 wt % doped films in mCBPCN (λ_exc_ = 355 nm).

The 10 wt % doped films in mCBPCN of **DMAC-TRZ**, **CNPh-DMAC-TRZ**, **CF**_**3**_**Ph-DMAC-TRZ**, **dPh-DMAC-TRZ**, ^***t***^**BuPh-DMAC-TRZ**, and **OMePh-DMAC-TRZ** emit at λ_PL_ of 505, 498, 503, 527, 531, and 539 nm, respectively (Figure 5a). The emission spectra of ^***t***^**BuPh-DMAC-TRZ**, **OMePh-DMAC-TRZ**, and **dPh-DMAC-TRZ** are red-shifted compared to the ones in 10 wt % mCP films, while the spectra of **CF**_**3**_**Ph-DMAC-TRZ** and **CNPh-DMAC-TRZ** do not change much (Table S6). The average room temperatures τ_PF_ are 7.8, 9.5, 10.5, 16.2, and 18.5 ns for **CNPh-DMAC-TRZ**, **CF**_**3**_**Ph-DMAC-TRZ**, **dPh-DMAC-TRZ**, ^***t***^**BuPh-DMAC-TRZ**, and **OMePh-DMAC-TRZ**, respectively (Table 1). As was observed in both solution and in mCP films, longer-lived prompt fluorescence is observed when the DMAC contains electron-donating substituents. The magnitude of the delayed component is similar to that of the doped mCP films for all emitters except ^***t***^**BuPh-DMAC-TRZ** and **OMePh-DMAC-TRZ**, which have a higher DF contribution in their decay profile. The average τ_DF_ is 1.2 ms, 163, 305, 2.64, and 1.99 μs for **CNPh-DMAC-TRZ**, **DF**_**3**_**Ph-DMAC-TRZ**, **dPh-DMAC-TRZ**, ^***t***^**BuPh-DMAC-TRZ**, and **OMePh-DMAC-TRZ**, respectively (Table 1). These lifetimes also follow the same trend as previously discussed for toluene, where the delayed lifetimes become shorter in emitters containing more electron-rich donors. **DMAC-TRZ** was measured in the same environment as a comparison, showing lifetimes that are intermediate, with average τ_PF_ and τ_DF_ of 12.1 ns and 15.6 μs, respectively.

**Table 1:** Kinetics Analysis^35^ of 10 wt % Doped Films of **DMAC-TRZ** and Its Five Derivatives in mCBPCN

R-DMAC-TRZ	*A*_PF_	τ_PF avg_^a^	*A*_DF_	τ_DF avg._^a^	Φ_PL_^b^	Φ_PF_^c^	Φ_DE_^c^	*k*_F avg._^a^	*k*_ISC avg._^a^	*k*_RISC avg._^a^
	(a.u.)	(×10^–9^ s)	(×10^–4^a.u.)	(×10^–6^ s)	(%)	(%)	(%)	(×10^7^ s^–1^)	(×10^7^ s^–1^)	(×10^5^ s^–1^)
^*t*^BuPh-	0.88	16.2	66.1	2.64	72 ± 5	32.6	39.4	6.19 ± 0.3	4.69 ± 0.5	7.59 ± 0.5
OMePh-	0.87	18.5	92.6	1.99	72 ± 5	34	38.1	5.41 ± 0.2	3.99 ± 0.4	9.59 ± 0.6
dPh-	0.96	10.5	0.29	305	70 ± 5	37.5	32.5	9.52 ± 0.3	6.37 ± 0.5	0.05 ± 0.002
CF_3_Ph-	0.98	9.5	0.64	163	70 ± 5	33	37	10.5 ± 0.6	7.71 ± 0.6	0.12 ± 0.01
CNPh-	0.95	7.8	0.06	1220	34 ± 3	17	17	12.8 ± 0.6	6.83 ± 0.3	0.01 ± 0.001
H-	0.89	12.1	9.87	15.6	87 ± 5	35.8	51.2	8.30 ± 0.4	7.08 ± 0.3	1.49 ± 0.1

a Average values were extracted from the averaging of the multiple exponentials used to fit the prompt and delayed lifetimes using the following equation:
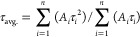

b Obtained using an integrating sphere, under a nitrogen atmosphere, λ_exc_ = 340 nm.

c Obtained from the integrated prompt and delayed emission of the TRPL decays.^42^

The decay kinetics were calculated following the method of Tsuchiya et al. (Table 1).^35^ All materials possess *k*_F_ and a *k*_ISC_ of the order of 10^7^–10^8^ s^–1^. The *k*_RISC_ were estimated to be 9.2 × 10^5^ and 7.8 × 10^5^ s^–1^ for ^***t***^**BuPh-DMAC-TRZ** and **OMePh-DMAC-TRZ**, respectively. These rates are four times faster compared to that of **DMAC-TRZ** (*k*_RISC_ = 2.2 × 10^5^ s^–1^), and 1 order of magnitude faster than the *k*_RISC_ of **CF**_**3**_**Ph-DMAC-TRZ**, **dPh-DMAC-TRZ**; the slowest *k*_RISC_ was observed for **CNPh-DMAC-TRZ** (Table 1). The trend in estimated *k*_RISC_ values aligns well with the relative magnitude of Δ*E*_ST_ (Figure S39).

### Orientation Measurements

**Figure 6:**
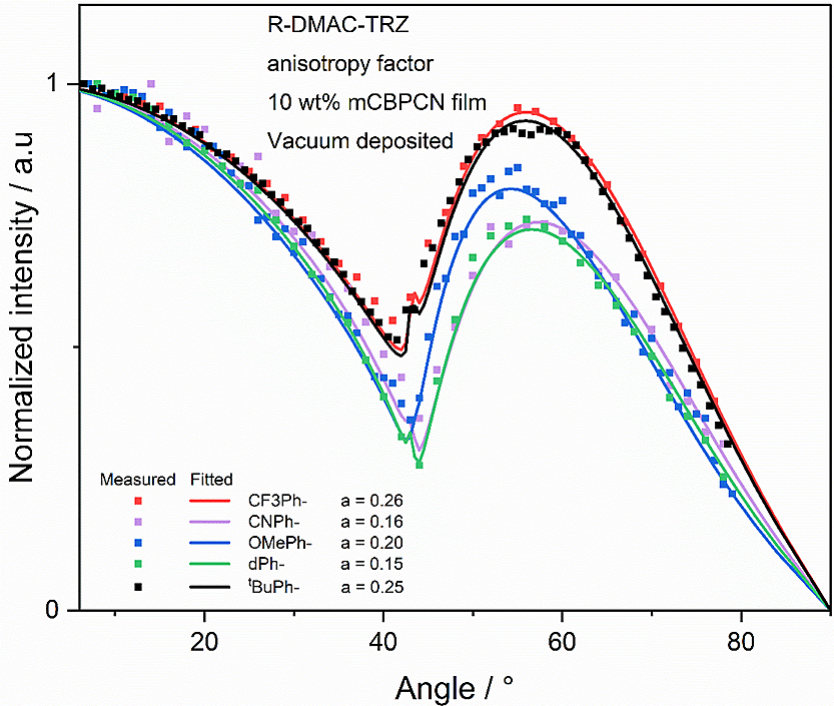
Angle-resolved photoluminescence measurement of **CNPh-DMAC-TRZ**, **CF**_**3**_**Ph-DMAC-TRZ**, **dPh-DMAC-TRZ**, ^***t***^**BuPh-DMAC-TRZ**, and **OMePh-DMAC-TRZ** in evaporated 10 wt % doped films in mCBPCN. The dotted line shows the measurement, and the continuous line of the matching color shows a fit using the dipole emission model, yielding the anisotropy factor, *a* (data taken at λ_PL_ = 490, 500, 520, 521, and 539 nm for **CNPh-DMAC-TRZ**, **CF**_**3**_**Ph-DMAC-TRZ**, **dPh-DMAC-TRZ**, ^***t***^**BuPh-DMAC-TRZ**, **OMePh-DMAC-TRZ**, respectively).

The orientation of the transition dipole moment (TDM) of the series of derivatives was measured in evaporated 10 wt % doped films in mCBPCN (Figure 6 and Table 2). All materials exhibit a preferential horizontal orientation of their TDM, with *a* values of 0.16, 0.26, 0.15, 0.25, and 0.20 for **CNPh-DMAC-TRZ**, **CF**_**3**_**Ph-DMAC-TRZ**, **dPh-DMAC-TRZ**, ^***t***^**BuPh-DMAC-TRZ**, and **OMePh-DMAC-TRZ**, respectively. The *a* value of **DMAC-TRZ** in the same environment of 0.21 lies in between those of the five derivatives.^4^ As previously mentioned, Tenopala-Carmona et al.^12^ found that generally for emitters with MW > 600 g/mol, the anisotropy factor improves with higher MW, higher *x*_E_/*x*_H_ (ratio of the length of the emitter to the host), and lower *z*_E_ (thickness of the emitter)^12^ (Table S8). All five derivatives (as well as the parent compound **DMAC-TRZ**) have the same *z*_E_ value; thus, it will not be considered in this analysis. The orthogonal conformation of the material means that the donor is responsible for the width of the material (*y*_E_) and the thickness of the material is dependent on the triazine, which is the same in all materials. The material with the lowest *a* value is **dPh-DMAC-TRZ**, which can be rationalized as it is the heaviest material (MW of 821.02 g/mol) and has the highest *x*_E_/*x*_H_ value of the series (*x*_E_/*x*_H_ = 1.35). However, the material with the second lowest *a* value is **CNPh-DMAC-TRZ**, which is the lightest material (MW of 718.84 g/mol) and has the smallest *x*_E_/*x*_H_ value of the series (*x*_E_/*x*_H_ = 1.08). The other materials that are categorized, in increasing *a*, are **OMePh-DMAC-TRZ** (*a* = 0.19), ^***t***^**BuPh-DMAC-TRZ** (*a* = 0.25), and **CF**_**3**_**Ph-DMAC-TRZ** (*a* = 0.26); no discernible trend was identified for these materials. Furthermore, **DMAC-TRZ** (MW = 516.63 g/mol) is even lighter than all its derivatives but still has an appreciable orientation factor (*a* = 0.21). Thus, the simple geometrical arguments do not apply here.

**Table 2:** Orientation Data of the Five Derivatives Obtained from Angle-Resolved Photoluminescence Measurements in Evaporated 10 wt % Doped Films in mCBPCN

emitter	*a*^a^	θ_h_^b^	*S*^c^
CNPh-DMAC-TRZ	0.16 ± 0.019	0.84	–0.275
CF_3_Ph-DMAC-TRZ	0.26 ± 0.008	0.74	–0.11
dPh-DMAC-TRZ	0.15 ± 0.010	0.85	–0.29
^*t*^BuPh-DMAC-TRZ	0.25 ± 0.010	0.75	–0.13
OMePh-DMAC-TRZ	0.20 ± 0.022	0.80	–0.22
DMAC-TRZ	0.21 ± 0.020	0.79	–0.19

a Anisotropy factor.

b Fraction of horizontal dipole (θ_h_ = 1 – *a*).

c Orientation order parameter (*S* = (3*a* – 1)/2.

### OLEDs

OLEDs were fabricated with the following architecture: ITO/NPB (35 nm)/NPB:mCBP 1:1 (5 nm)/mCBP (10 nm)/mCBPCN:**R-DMAC-TRZ***x* wt % (30 nm)/T2T (10 nm)/T2T:LiQ 1:1 (35 nm)/LiQ (1 nm)/Al (100 nm) (Figure S41). Initially, the OLEDs of ^***t***^**BuPh-DMAC-TRZ** at different doping concentrations were fabricated (Figure S42). While the efficiency roll-off improves at higher emitter doping concentrations, the overall efficiency significantly decreases with increasing emitter doping, from 10 wt % to the nondoped device, with an EQE_max_ of 28, 21, 18.6, 11.8, and 6.1% for the devices with 10, 20, 30, 60 wt % doping and nondoped device, respectively. From the 10 wt % to the nondoped device, a redshift of ca. 10 nm is observed. Thus, OLEDs of the five derivatives, at 10 wt % concentration, were fabricated.

**Figure 7:**
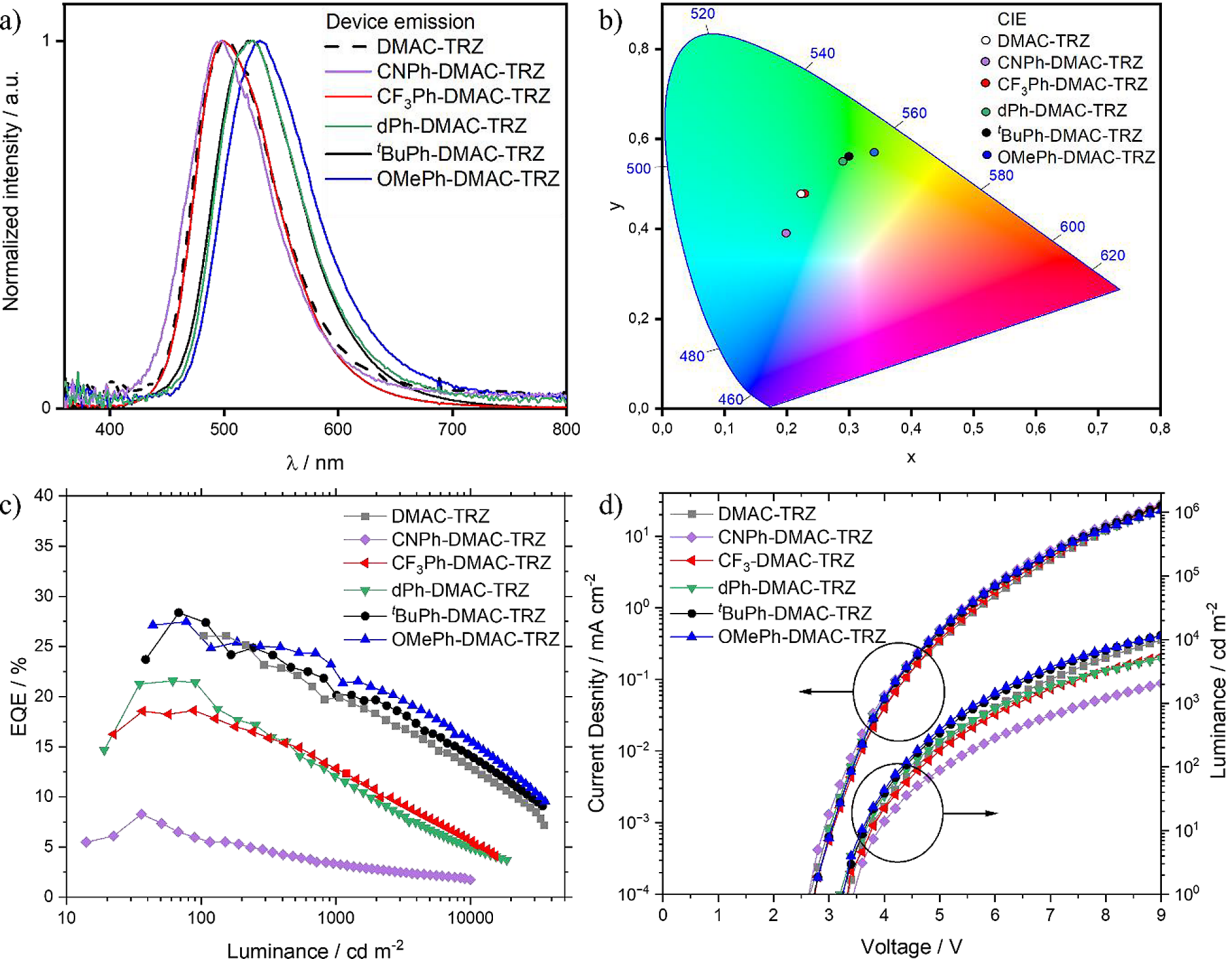
(a) Electroluminescence spectra (at 1000 cd m^–2^) of **CNPh-DMAC-TRZ**, **CF**_**3**_**Ph-DMAC-TRZ**, **dPh-DMAC-TRZ**, ^***t***^**BuPh-DMAC-TRZ**, **OMePh-DMAC-TRZ** 10 wt % doped in mCBPCN; (b) CIE coordinates of the devices (the CIE coordinates of the devices with **DMAC-TRZ** and **CF**_**3**_**Ph-DMAC-TRZ** are identical, precisely under the white dot); (c) EQE vs luminance curves of the OLEDs with **DMAC-TRZ** and its derivatives; and (d) current density vs voltage vs luminance curves of the OLEDs.

The performance of the OLEDs is summarized in Table 3. All devices have similar turn-on voltage between 3.2 and 3.4 V, and the electroluminescence maxima (λ_EL_) followed the same trend observed in the thin film SSPL (Figure 7a). The λ_EL_ was 501, 496, 498, 525, 526, and 531 nm for the **DMAC-TRZ**, **CNPh-DMAC-TRZ**, **CF**_**3**_**Ph-DMAC-TRZ**, **dPh-DMAC-TRZ**, ^***t***^**BuPh-DMAC-TRZ**, and **OMePh-DMAC-TRZ** OLEDs, respectively.

**Table 3:** Performance Metrics of the Corresponding OLEDs

emitter	λ_EL_/nm^a^	*V*_on_/V	EQE_max_/%	EQE_1000_/%	CE_max_/cd A^–1^	PE_max_/lm W^–^^1^	*L*_max_/cd m^–2^	CIE(*x*, *y*)^a^	LT_85_/h^a^
**DMAC-TRZ**	501	3.3	26 ± 3	20 ± 1.1	67 ± 6	45.5 ± 6	21,600	0.22; 0.48	1.1
**CNPh-DMAC-TRZ**	496	3.4	8.3 ± 1	3.3 ± 0.3	22.5 ± 3	16 ± 3	10,000	0.20; 0.39	0.9
**CF**_**3**_**Ph-DMAC-TRZ**	498	3.3	18.5 ± 1.9	12.8 ± 0.8	54.6 ± 5	40.8 ± 5	13,200	0.22; 0.48	0.2
**dPh-DMAC-TRZ**	525	3.2	21.6 ± 3.1	12.0 ± 1	63 ± 6	49.5 ± 6	16,200	0.29; 0.55	1.9
^***t***^**BuPh-DMAC-TRZ**	526	3.2	28 ± 3.3	21.5 ± 1.4	76.6 ± 6	50.1 ± 6	36,000	0.30; 0.56	0.6
**OMePh-DMAC-TRZ**	531	3.2	27.4 ± 3.3	22.1 ± 1.4	80.3 ± 6	54.9 ± 6	29,300	0.34; 0.57	0.3

a Recorded at 10 mA/cm^2^, time necessary for the emission intensity (*I*) to decrease to 85% of its initial value (*I*_0_), *I*_0_ is the luminance value taken at a current density of 10 mA/cm^2^, which corresponds to 957, 2891, 2860, 4113, 5099, and 5743 cd m^–2^ for **CNPh-DMAC-TRZ**, **CF**_**3**_**Ph-DMAC-TRZ**, **dPh-DMAC-TRZ**, **DMAC-TRZ**, ^***t***^**BuPh-DMAC-TRZ**, and **OMePh-DMAC-TRZ**.

The ^***t***^**BuPh-DMAC-TRZ** and **OMePh-DMAC-TRZ** devices showed the highest EQE_max_ of 28 and 27.4%, respectively, which is an improvement over the 26% for the device with **DMAC-TRZ** (Figure 7c). The **CF**_**3**_**Ph-DMAC-TRZ** and **dPh-DMAC-TRZ** devices showed lower EQE_max_ values of 18.5 and 21.6%, respectively, while the worst-performing device was the one with **CNPh-DMAC-TRZ**, with an EQE_max_ value of 8.3%, which reflects the poor exciton harvesting ability of the emitter. These results are in good agreement with the photophysical properties of the six emitters. At 1000 cd m^–2^ the EQE of the six OLEDs was 20, 3.3, 12.8, 12.0, 21.5, and 22.1% for **DMAC-TRZ, CNPh-DMAC-TRZ**, **CF**_**3**_**Ph-DMAC-TRZ**, **dPh-DMAC-TRZ**, ^***t***^**BuPh-DMAC-TRZ**, and **OMePh-DMAC-TRZ** respectively (Table 3). This verifies that at higher current densities, the device roll-off is proportional to the triplet harvesting efficiency (*k*_RISC_) of each individual emitter (^***t***^**BuPh-DMAC-TRZ**, **OMePh-DMAC-TRZ** > **DMAC-TRZ** > **CF**_**3**_**Ph-DMAC-TRZ**, **dPh-DMAC-TRZ** > **CNPh-DMAC-TRZ**).

Considering the variation of the EQE_max_ values across the OLEDs with the six emitters we have performed simulations of the light outcoupling factor as a function of TDM orientation (Figure S43). The light outcoupling efficiency was estimated to be 26, 22, 28, 24, 24, and 26%, for **CNPh-DMAC-TRZ**, **CF**_**3**_**Ph-DMAC-TRZ**, **dPh-DMAC-TRZ**, **DMAC-TRZ**, ^***t***^**BuPh-DMAC-TRZ**, and **OMePh-DMAC-TRZ**, respectively (Table S9). These results predict a small improvement in the outcoupling efficiency in **CNPh-DMAC-TRZ**, **dPh-DMAC-TRZ**, and **OMePh-DMAC-TRZ** compared to **DMAC-TRZ**. However, despite their extended molecular length, the light outcoupling efficiencies of the devices with **CF**_**3**_**Ph-DMAC-TRZ** and ^***t***^**BuPh-DMAC-TRZ** are, respectively, similar, or only slightly lower than the device with **DMAC-TRZ**. This makes the triplet harvesting efficiency of these emitters the main parameter that determines the EQE_max_ values of the devices. It is furthermore apparent from the light outcoupling simulations that the best devices with their measured EQEs in the range of 26–28% must basically have balanced carrier recombination as well as complete triplet harvesting.

## Conclusions

In this study, we report five modified **DMAC-TRZ** derivatives, **CNPh-DMAC-TRZ**, **CF**_**3**_**Ph-DMAC-TRZ**, **dPh-DMAC-TRZ**, ^***t***^**BuPh-DMAC-TRZ**, and **OMePh-DMAC-TRZ**, bearing substituted acridine donors, to explore the impact of the substitution on their photophysical and orientation properties and the impact in the performance of the OLEDs. The emission was modulated from sky blue to green in 10 wt % doped films in mCBPCN as a function of the nature of the substituent. The trend in tuning the energy of the lowest singlet excited state did not match that of tuning the energy of the lowest triplet excited state, as these states have contrasting charge-transfer and locally excited characters, respectively. This behavior results in a wide variation of the Δ*E*_ST_ across the five derivatives, and accordingly of *k*_RISC_ values (ranging from 10^3^ to 10^6^ s^–1^ for **CNPh-DMAC-TRZ** to **OMePh-DMAC-TRZ**, respectively), while the Φ_PL_ values are maintained around 70% in all compounds, except **CNPh-DMAC-TRZ** (34%) and **DMAC-TRZ** (87%). Angle-resolved PL measurements of the 10 wt % emitter in mCBPCN films revealed preferentially horizontally orientated TDMs for **CNPh-DMAC-TRZ**, **dPh-DMAC-TRZ**, and **OMe-DMAC-TRZ** compared to **DMAC-TRZ**; however, this is not the case for the **CF**_**3**_**Ph-DMAC-TRZ** and ^***t***^**BuPh-DMAC-TRZ** films. This translates to a small improvement in the light outcoupling efficiency for the OLEDs with **CNPh-DMAC-TRZ**, **dPh-DMAC-TRZ**, and **OMe-DMAC-TRZ** (estimated from the light outcoupling simulations), compared to the device with **DMAC-TRZ**; however, this was not the case for the devices with **CF**_**3**_**Ph-DMAC-TRZ** and ^***t***^**BuPh-DMAC-TRZ**.

Our study demonstrates that by decorating the donor with aryl groups in a D–A TADF emitter, the probability of achieving a higher horizontal orientation of the TDM can be improved but is highly dependent on the nature of the attached aryl group. Nevertheless, the dominant effect on OLED efficiency results from triplet harvesting whereby ^***t***^**BuPh-DMAC-TRZ** and **OMe-DMAC-TRZ** have the fastest RISC rates and, consequently, show the highest EQE_max_ of ∼28% and suppressed efficiency roll-off compared to the reference **DMAC-TRZ** OLEDs.

## Data Availability

The research data supporting this publication can be accessed at https://doi.org/10.17630/020b200d-1928-4aea-8ebf-cb20c6cfc201.

## Supporting Information Available

General methods; experimental section (^1^H and ^13^C NMR spectra, HRMS, EA, and HPLC of all target compounds); supplementary computational data; supplementary photophysical data; supplementary OLED data; outcoupling efficiency estimation; supplementary OLED lifetime data (PDF)
XYZ combined data (TXT)
