# Supplementary material for: Aryl-Substituted Acridine Donor Derivatives Modulate the Transition Dipole Moment Orientation and Exciton Harvesting Properties of Donor–Acceptor TADF Emitters
Source: J Phys Chem C Nanomater Interfaces. 2024 Aug 20;128(34):14429–41. doi: 10.1021/acs.jpcc.4c03344 (PMC11372754; doi:10.1021/acs.jpcc.4c03344)
Supplement: Supplementary file 1 — jp4c03344_si_001.pdf [file jp4c03344_si_001.pdf]

Aryl-Substituted Acridine Donor Derivatives Modulate the Transition Dipole Moment Orientation and Exciton Harvesting Properties of Donor-Acceptor TADF Emitters

Ettore Crovini,[a] Kleitos Stavrou,*[b] Prakhar Sahay,[c] Binh Minh Nguyễn,[c] Thomas Comerford,[a] Stuart Warriner,[d] Wolfgang Brütting,*[c] Andrew Monkman,*[b] and Eli Zysman-Colman*[a]

[a] Organic Semiconductor Centre, EaStCHEM School of Chemistry, University of St Andrews, St Andrews, Fife, KY16 9ST, United Kingdom. E-mail: eli.zysman-colman@st-andrews.ac.uk; Web: http://www.zysman-colman.com; Tel: +44 (0)1334 463826.
[b] OEM group, Department of Physics, Durham University, South Road, Durham DH1 3LE, United Kingdom. Email: kleitos.stavrou@durham.ac.uk; a.p.monkman@durham.ac.uk
[c] Experimental Physics IV, Institute of Physics, University of Augsburg, Augsburg, 86159, Germany. Email: wolfgang.bruetting@physik.uni-augsburg.de
[d] School of Chemistry, University of Leeds, Woodhouse Lane, Leeds, LS2 9JT, United Kingdom.

SUPPORTING INFORMATION

Experimental Section **2**
DFT Calculations **31**
Optoelectronic characterization **35**
OLEDs **43**
Light outcoupling efficiency simulations **44**
References **45**

Experimental Section

2-(4-bromophenyl)-4,6-diphenyl-1,3,5-triazine (BrPh-dPh-TRZ)

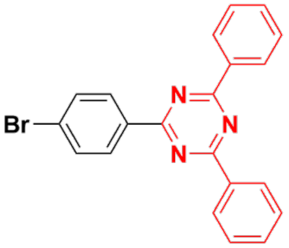

BrPh-dPh-TRZ was prepared following a literature procedure.[18] A mixture of 4-bromobenzoyl chloride (5.0 g, 22.78 mmol, 1 equiv.) and benzonitrile (4.699 mL, 45.57 mmol, 2 equiv.), in DCM (30 mL), was cooled to 0°C in an ice bath and stirred for 30 min. Antimony pentachloride (2.924 mL, 22.78 mmol, 1 equiv.) was then added dropwise with a glass pipette. The solution immediately turned from white to canary yellow. The solution was stirred at room temperature over-night. The yellow solid was filtered and washed with dichloromethane. With an ice-bath, the yellow solid was added to a 35% ammonia solution (175 mL) and stirred in the ice bath for 30 min, and then 3h at room temperature. The white solid was then filtered and washed with cold MeOH to yield the desired product. (8.701 g). **Yield**: 98%; **Mp**: 200-210°C ; **R_f:** 0.90 (ethyl acetate/cyclohexane = 1:4); **^{1}H NMR** (500 MHz, $CDCl_3$) δ: 8.78 – 8.76 (dt, *J*=6.9, 1.4, 1.1, 4H), 8.67-8.64 (dt, *J*=8.6, 2.4, 1.8, 2H), 7.73-7.70 (dt, *J*= 8.5, 2.5, 1.9, 2H), 7.64-7.57 (m, 6H); **^{13}C NMR** (126 MHz, $CDCl_3$) δ: 171.89, 171.00, 136.15, 135.33, 132.82, 132.06, 130.62, 129.12, 128.84, 127.63; **GC-MS retention time:** 13.529 min; **purity:** >99%; **Mass Theoretical:** ($C_{21}H_{14}BrN_3$) 387.04; **Mass found:** 387.

Spectral data matches those previously reported in the literature.[19]

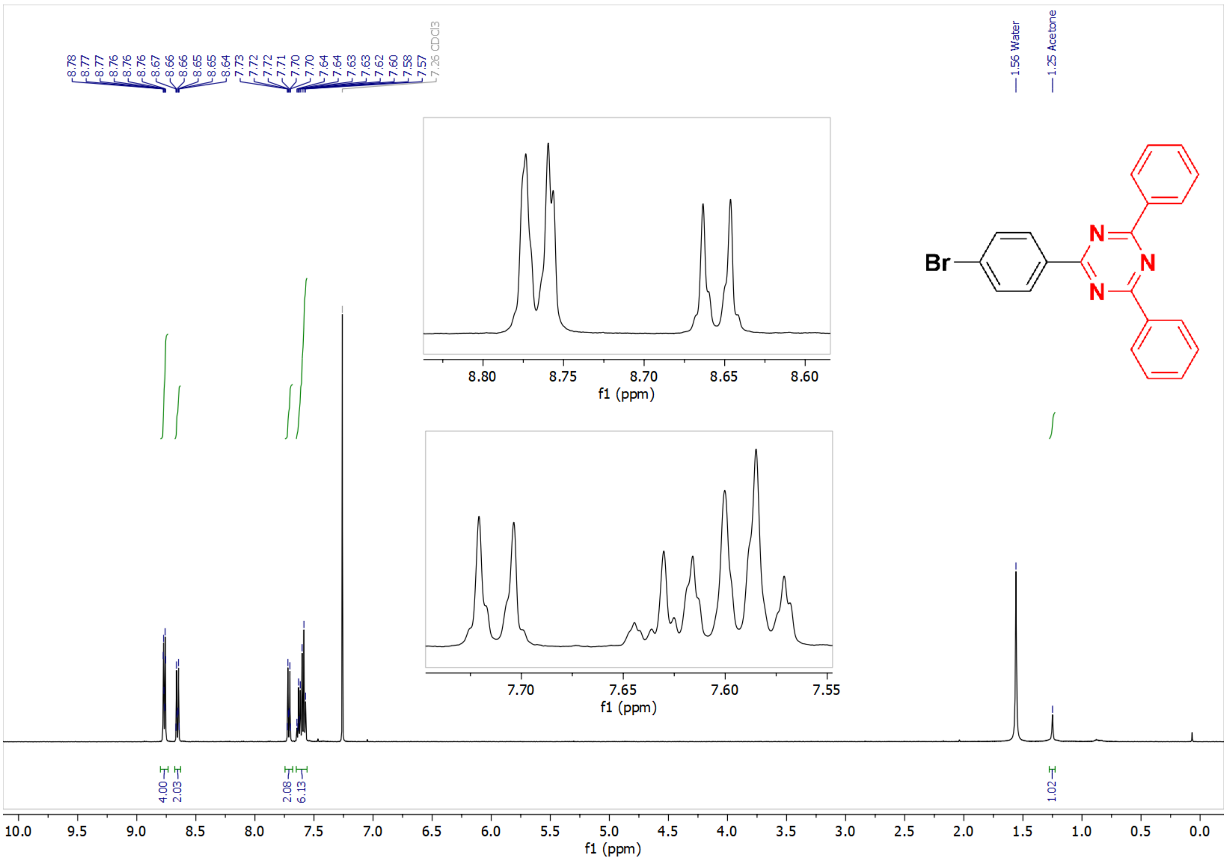

Figure **S1.** ^{1}H NMR of **BrPh-dPh-TRZ** in $CDCl_3$.

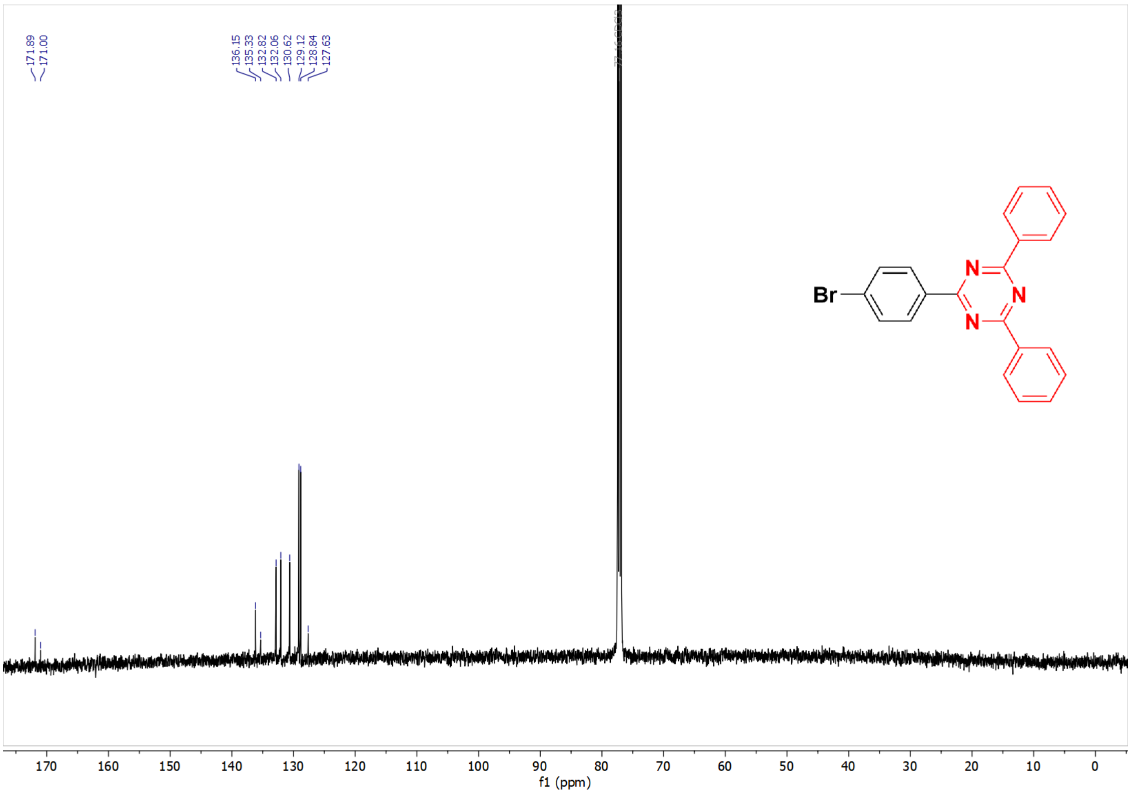

Figure **S2.** ^{13}C NMR of **BrPh-dPh-TRZ** in $CDCl_3$.

Sample Information

Analyzed by	: Admin
Analyzed	: 30/05/2022 13:43:25
Sample Type	: Unknown
Level #	: 1
Sample Name	: EC-Br-TRZ
Sample ID	: EC-Br-TRZ
IS Amount	: [1]=1
Sample Amount	: 1
Dilution Factor	: 1
Vial #	: 127
Injection Volume	: 1.00
Data File	: S:\Ettore\Missing GCMS\EC-dBr-Ph-TRZ.qgd
Org Data File	: S:\Ettore\Missing GCMS\EC-dBr-Ph-TRZ.qgd
Method File	: S:\standard method 1 for RTX-1 column 330 injection-long.qgm
Org Method File	: S:\standard method 1 for RTX-1 column 330 injection-long.qgm
Report File	:
Tuning File	: C:\GCMSsolution\System\Tune1\1 day after reinstall 09062021.qgt
Modified by	: Admin
Modified	: 30/05/2022 14:18:08

Chromatogram EC-Br-TRZ S:\Ettore\Missing GCMS\EC-dBr-Ph-TRZ.qgd

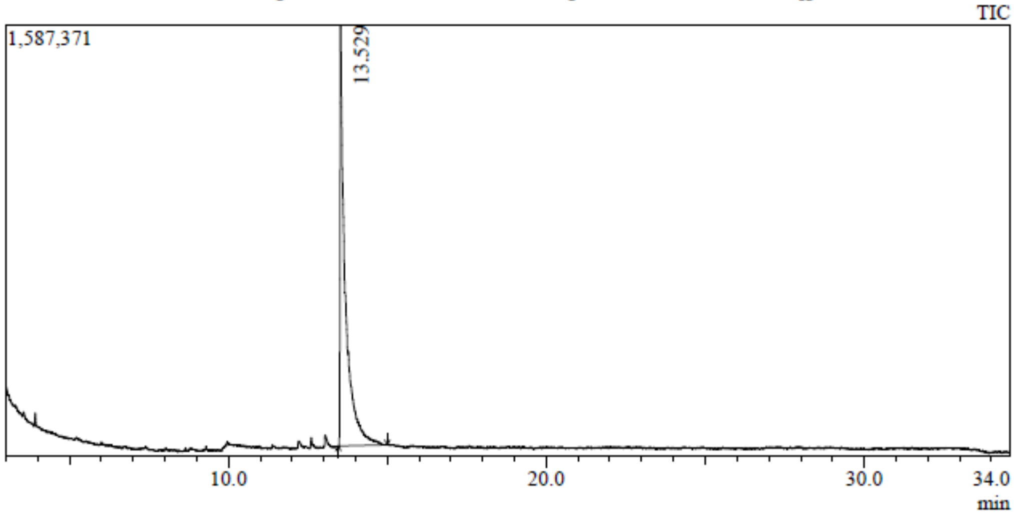

Peak Report TIC

Peak#	R.Time	Area	Area%	Height	A/H	Base m/z	Base Int.
1	13.529	17277601	100.00	1551203	11.14	103.10	301379
		17277601	100.00	1551203			

Line#:1 R.Time:13.567(Scan#:1269)
MassPeaks:556
RawMode:Single 13.567(1269) BasePeak:103.10(254314)
BG Mode:None Group 1 - Event 1 Scan

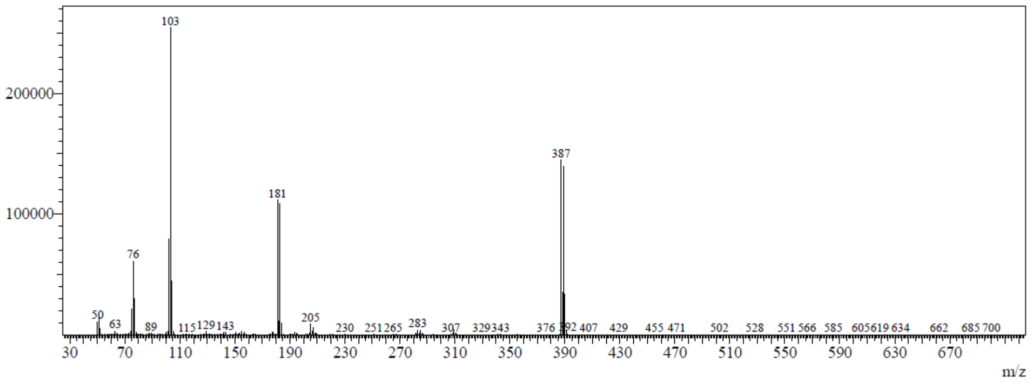

Figure **S3**. GC-MS trace of **BrPh-dPh-TRZ**.

10-(4-(4,6-diphenyl-1,3,5-triazin-2-yl)phenyl)-9,9-dimethyl-9,10-dihydroacridine (DMAC-TRZ)

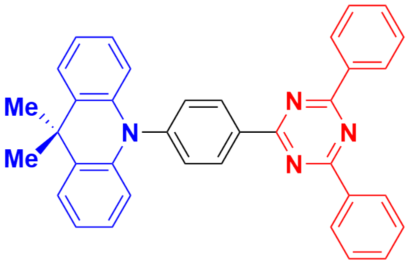

To a schlenk flask fitted with a reflux condenser was added DMAC (2.0 g, 9.6 mmol, 1 equiv.), 2-(4-bromophenyl)-4,6-diphenyl-1,3,5-triazine (**BrPh-dPh-TRZ**) (4.1 g, 10.5 mmol, 1.1 equiv.), tribasic potassium carbonate (4.0 g, 28.8 mmol, 3 equiv.) palladium(II) acetate (214.5 mg, 0.96 mmol, 0.1 equiv.), tri-*tert*-butylphosphonium tetrafluoroborate (1.1 g, 3.8 mmol, 0.4 equiv.), and dry toluene (200 mL). The flask was connected to a Schlenk line via a reflux condenser and the mixture was heated to reflux, under N_2 atmosphere. After 48 h, the reaction was diluted with water, and the organic layer was extracted with DCM (50 mL × 3). The combined organic extracts were washed with brine, dried over Mg_2SO_4 and concentrated by rotary evaporation. The crude was then washed with cold pentane (250 mL) and cold diethyl ether (250 mL) to achieve the desired product as a light brown solid (2.1 g). **Yield:** 42%; **Mp**: 250-253°C; **R*f*:** 0.76 (ethyl acetate/cyclohexane = 1 :4); **^{1}H NMR** (500 MHz, $CDCl_3$) δ: 9.04 – 9.02 (dt, *J*= 8.3, 2.3, 2.1, 2H), 8.83-8.81 (dt, *J*= 6.6, 1.4, 1.4, 4H), 766 – 7.56 (m, 8H), 7.50-7.48 (dd, *J*= 7.5, 1.7, 2H), 7.01-6.94 (m, 4H), 6.39-6.37 (dd, *J*= 8.0, 1.3, 2H), 1.73 (s, 6H); **^{13}C NMR** (126 MHz, $CDCl_3$) δ: 172.00, 171.22, 140.74, 136.23, 136.21, 132.86, 131.75, 131.68, 130.36, 129.17, 128.88, 126.60, 125.51, 121.98, 114.33, 36.19, 31.46.

Spectral data match those previously reported in the literature.[20]

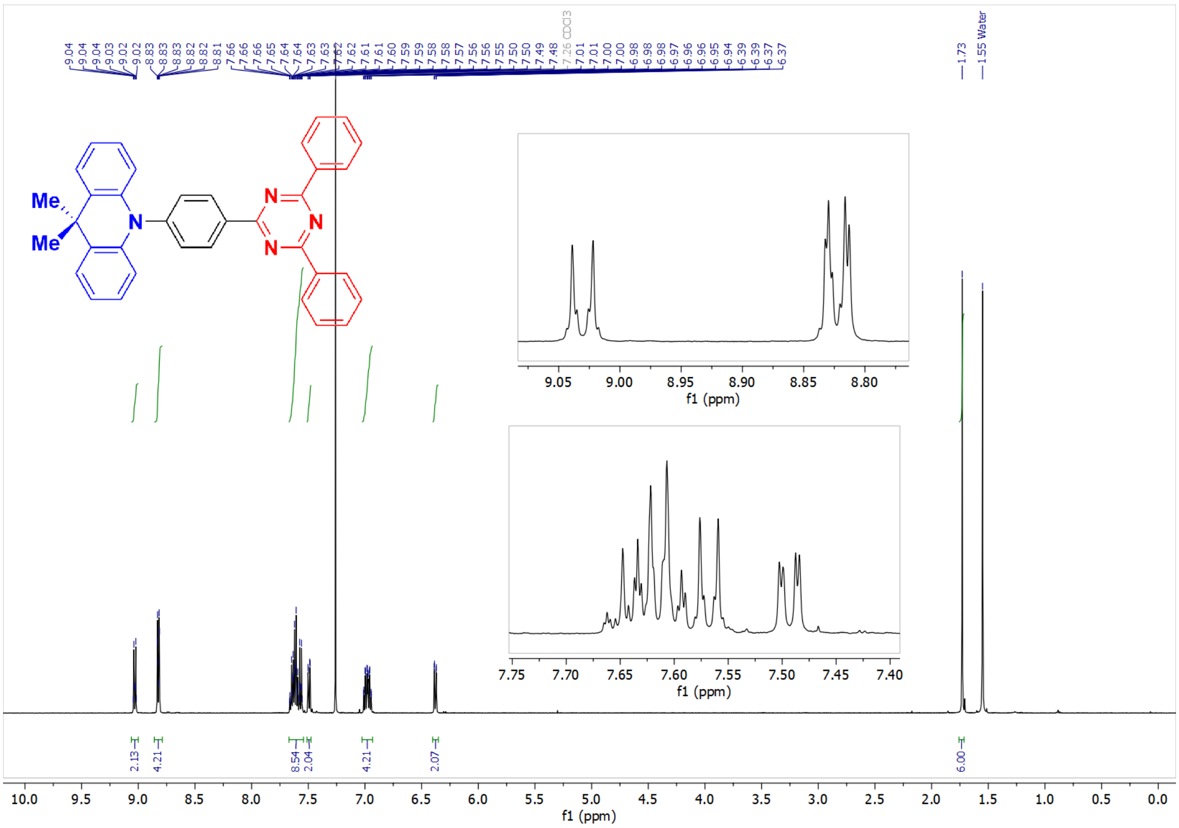

Figure **S4**. ^{1}H NMR of **DMAC-TRZ** in $CDCl_3$.

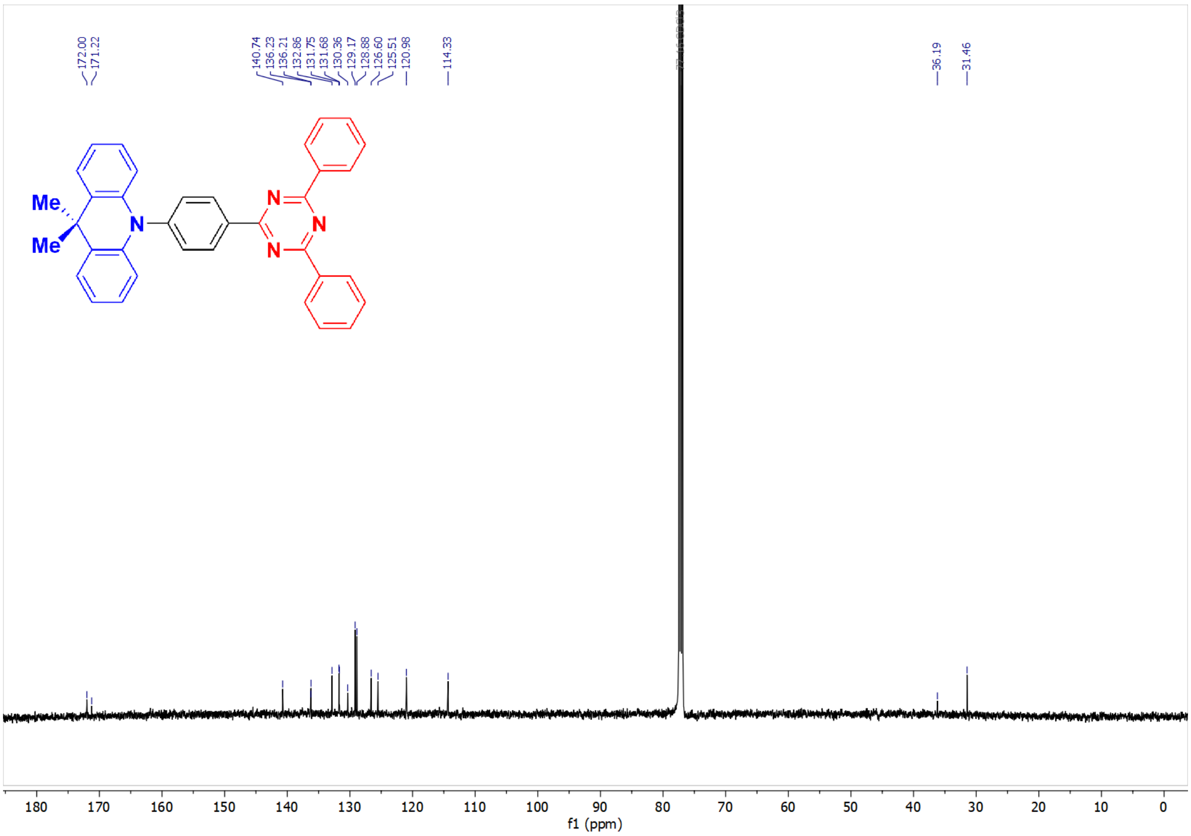

Figure **S5**. ^{13}C NMR of **DMAC-TRZ** in $CDCl_3$.

2,7-dibromo-10-(4-(4,6-diphenyl-1,3,5-triazin-2-yl)phenyl)-9,9-dimethyl-9,10-dihydroacridine (dBr-DMAC-TRZ)

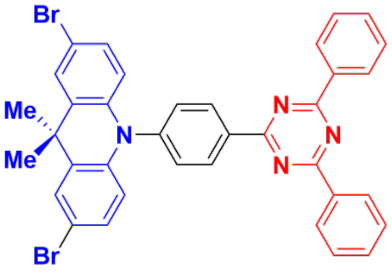

To a round bottom flask **DMAC-TRZ** (2.06 g, 3.99 mmol, 1 equiv.) and THF (90 mL) were added. The solution was cooled in an ice bath and a solution of *N*-bromosuccinimide (NBS, 1.63 g, 9.17 mmol, 2.3 equiv.) in THF (100 mL) was slowly added. The reaction mixture turned red, then yellow and then brown with the continuous addition of the NBS solution. The solution was stirred overnight, at room temperature, in darkness. The reaction was diluted with water, extracted with DCM (3 × 50 mL). The organic phase was washed with brine, dried over $MgSO_4$ and concentrated under reduced pressure. The crude was then washed with cold pentane (250 mL) and cold diethyl ether (250 mL) to achieve the desired product as a light brown solid (2.22 g). **Yield:** 75%; **Mp**: decomposition at 250 °C; **R*f*:** 0.83 (ethyl acetate/cyclohexane= 1:4); **^{1}H NMR** (300 MHz, $CDCl_3$) δ: 9.05-9.02 (dt, *J*= 8.7, 2.3, 1.9, 2H), 8.83-8.80 (dt, *J*= 6.3, 1.6, 1.3, 6H), 7.66 – 7.59 (m, 4H), 7.55-7.54 (d, *J*= 2.3, 2H), 7.52-7.49 (dt, *J*= 8.2, 2.8, 1.7, 2H), 7.10-7.06 (dd, *J*= 8.8, 2.3, 2H), 6.25-6.22 (d, *J*= 8.8, 2H), 1.68 (s, 6H). **^{13}C NMR** (126 MHz, $CDCl_3$) δ: 172.06, 170.99, 144.43, 139.58, 136.86, 136.10, 132.95, 131.98, 131.90, 131.31, 128.56, 128.17, 128.90, 128.36, 116.03, 113.77, 36.48, 31.17; **HR-MS** (APCI) $[M+H]^+$ **Calculated:** ($C_{36}H_{27}{}^{79}Br_2N_4^+$) 673.0579, **Found**: 673.0602.

Spectral data match those previously reported in the literature.[21]

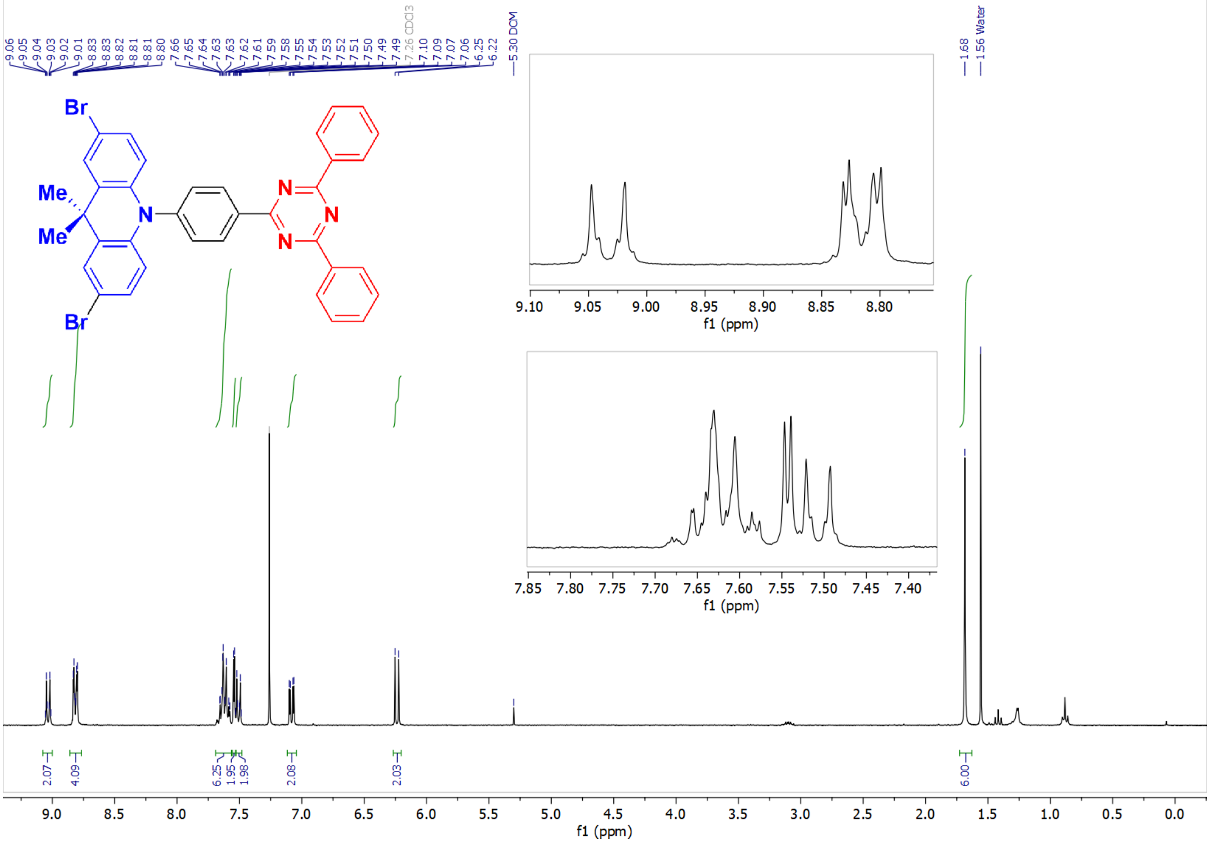

Figure **S6**. ^{1}H NMR of **dBr-DMAC-TRZ** in $CDCl_3$.

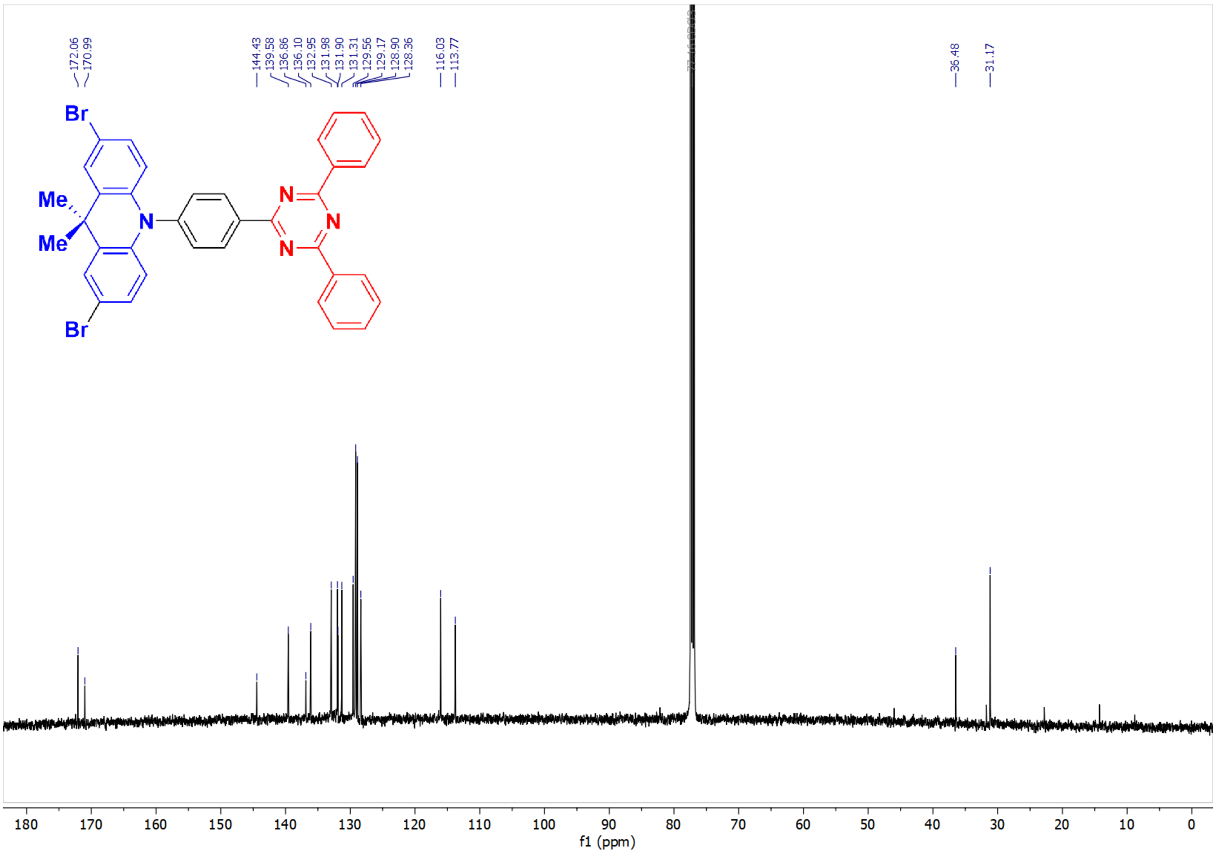

Figure **S7**. ^{13}C NMR of **dBr-DMAC-TRZ** in $CDCl_3$.

School of Chemistry Mass Spectrometry Service

**SampleID**		**Submitter**	
**Sample Description**			
**Analysis Name**	D:\Data\stuartwarriner\manual\dBrPh_DMAC_TRZ_a.d	**Supervisor**	
**Method**	DIP Pos 3.m	**Acquisition Date**	12/03/2022 17:43:07
**Instrument**	maXis impact **Source Type** APCI **Ion Polarity** Positive	**Scan Begin** 50 m/z	**Scan End** 2200 m/z

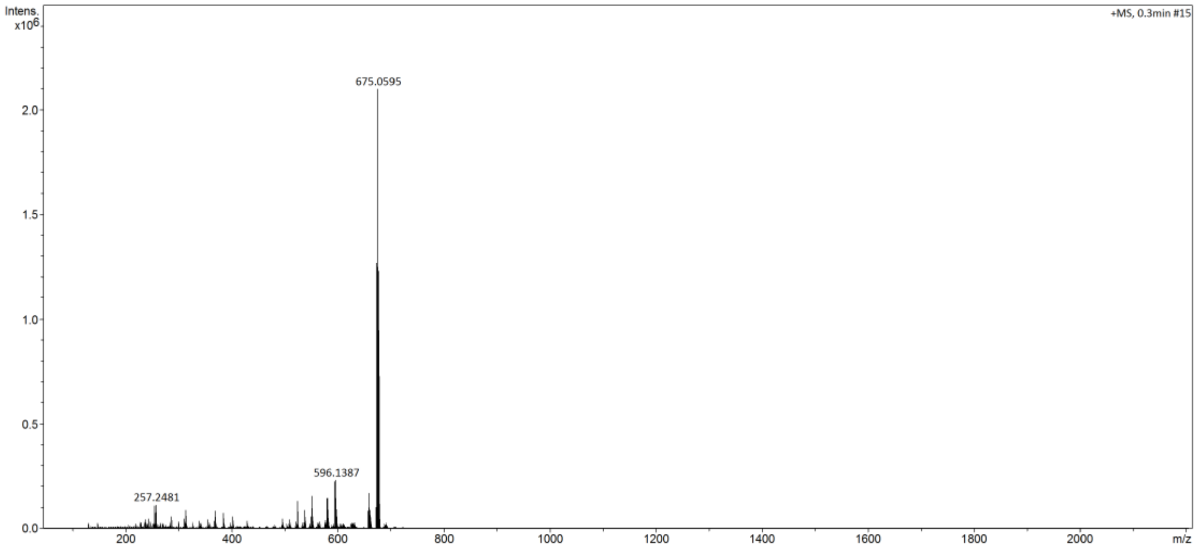

Bruker Compass DataAnalysis 4.3 Analysis Name dBrPh_DMAC_TRZ_a.d 12/03/2022 17:46:15 1 of 1

Figure **S8**. HRMS analysis report for **dBr-DMAC-TRZ**.

4,4'-(10-(4-(4,6-diphenyl-1,3,5-triazin-2-yl)phenyl)-9,9-dimethyl-9,10-dihydroacridine-2,7-diyl)dibenzonitrile (CNPh-DMAC-TRZ)

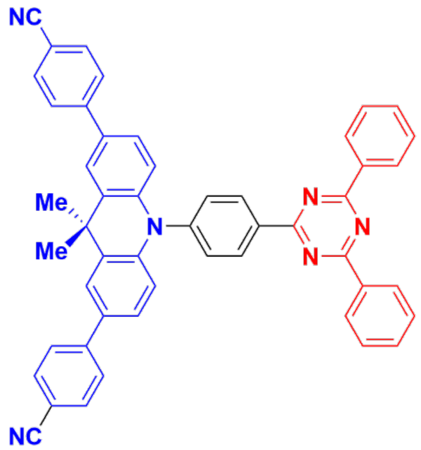

The same reaction procedure as ***ᵗ*BuPh-DMAC-TRZ** was followed but with a different boronic acid, (4-cyanophenyl)boronic acid (0.872 g, 5.931 mmol, 4 equiv.). After 72 h, the reaction was diluted with THF and filtered over celite to remove any unreacted palladium. The solution was diluted with water, and the organic layer was extracted with DCM (3 × 50 mL). The organic phase was washed with brine, dried over $MgSO_4$ and concentrated under reduced pressure. The target product was isolated as yellow solid after purification by column chromatography on silica gel using 5:1 hexane/EtOAc as eluent. It was then further purified *via* recrystallization in THF/MeOH. (0.448 g). The compound was further purified by gradient temperature sublimation. **Yield:** 42%; **Mp**: 390-395 °C; **R_f:** 0.82 (MeOH/DCM = 1:99); **^{1}H NMR** (500 MHz, $CDCl_3$) δ: 9.10-9.08 (d, *J*= 8.3, 2H), 8.84-8.82 (d, *J*= 7.0, 4H), 7.75 – 7.60 (m, 20H), 6.50-6.48 (d, *J*= 8.6, 2H), 1.85 (s, 6H); **^{13}C NMR** (126 MHz, $CDCl_3$) δ: 171.98, 170.89, 145.37, 144.31, 140.62, 136.87, 135.96, 132.87, 132.65, 131.97, 131.91, 131.28, 130.61, 129.05, 128.80, 126.96, 125.62, 124.58, 119.19, 115.05, 110.01, 36.42, 31.94; **HPLC retention time:** 10.365; **purity:** 100.00%; **HR-MS** (APCI) $[M+H]^+$ **Calculated:** ($C_{50}H_{35}N_6^+$) 719.2918, **Found:** 719.2947; **EA** ($C_{50}H_{34}N_6$) **Calculated** C: 83.54, H: 4.77, N: 11.69; **Found** C: 82.63, H: 4.78, N: 11.55. The carbon content is not within 0.4% error; however, the remaining characterization is consistent and demonstrates the identity and purity of the compound.

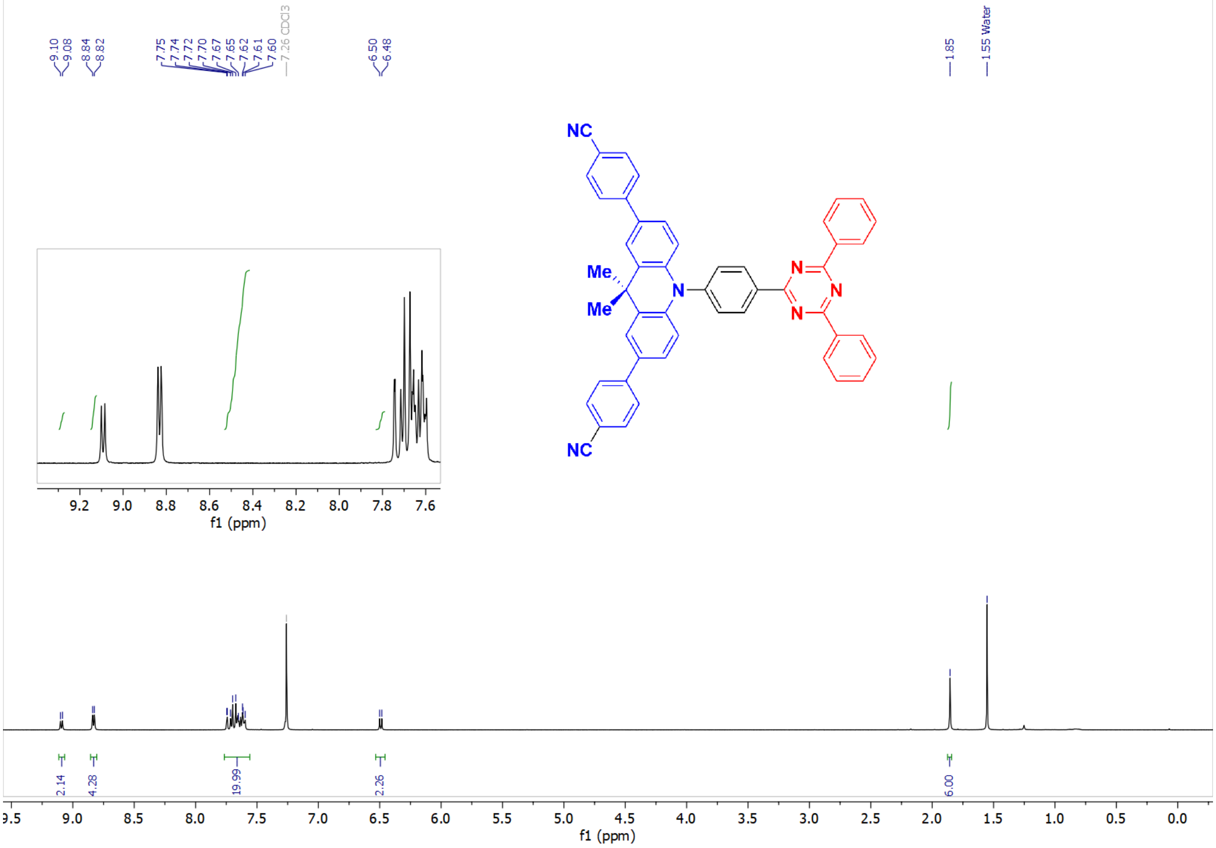

Figure **S9**. ^{1}H NMR of **CNPh-DMAC-TRZ** in $CDCl_3$.

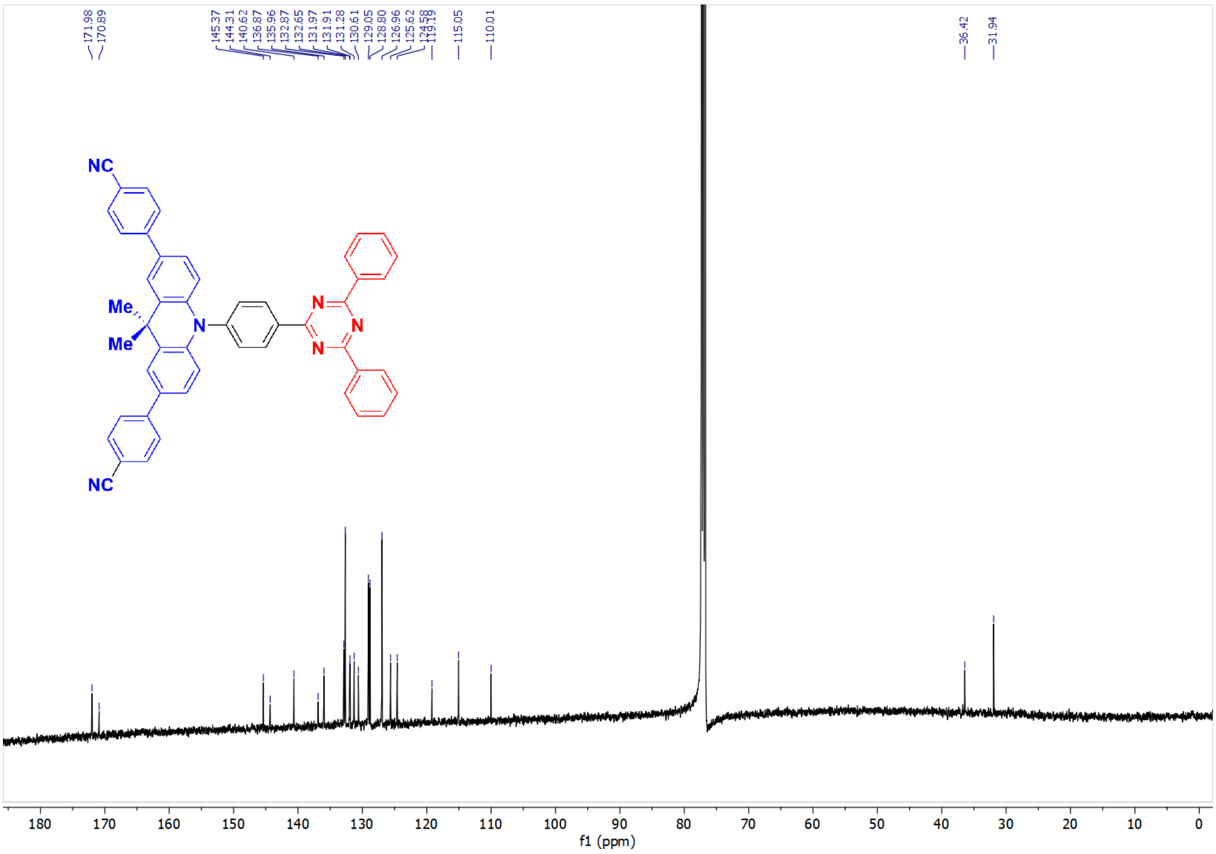

Figure **S10**. ^{13}C NMR of **CNPh-DMAC-TRZ** in $CDCl_3$.

HPLC Trace Report31May2022

<Sample Information>

Sample Name	: EC-4046-dCNPh-DT		
Sample ID	: EC-4046		
Method Filename	: 100% THF 20 mins 280nm - DH.lcm		
Batch Filename	: EC-4046-dCN.lcb		
Vial #	: 1-50	Sample Type	: Unknown
Injection Volume	: 10 uL		
Date Acquired	: 31/05/2022 11:12:10	Acquired by	: System Administrator
Date Processed	: 31/05/2022 11:32:13	Processed by	: System Administrator

<Chromatogram>

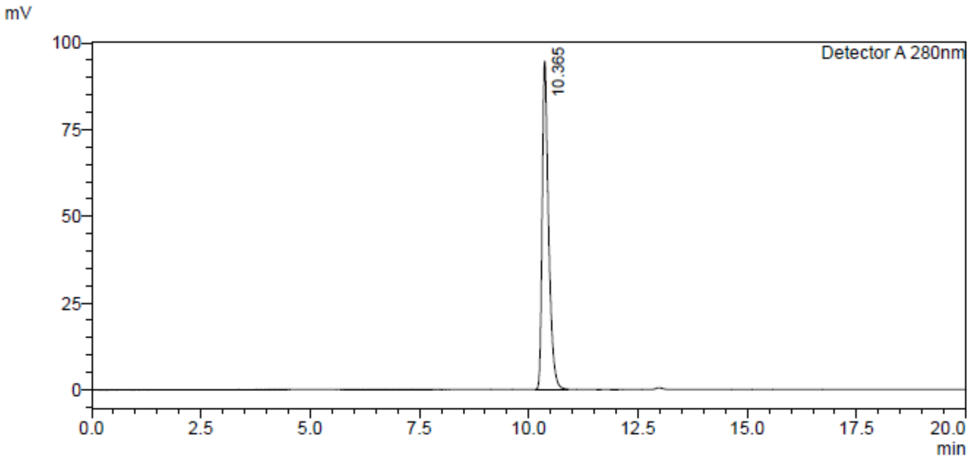

<Peak Table>

Detector A 280nm

Peak#	Ret. Time	Area	Height	Area%	Area/Height	Width at 5% Height
1	10.365	967160	94466	100.000	10.238	0.370
Total		967160	94466	100.000		

Figure **S11**. HPLC analysis report for **CNPh-DMAC-TRZ**.

School of Chemistry Mass Spectrometry Service

SampleID				Submitter			
Sample Description							
Analysis Name	D:\Data\stuartwarriner\manual\EC_4046_a.d			Supervisor			
Method	DIP Pos 3.m			Acquisition Date	18/05/2022 15:17:10		
Instrument	maXis impact	Source Type	APCI	Ion Polarity	Positive	Scan Begin	50 m/z
				Scan End	2200 m/z		

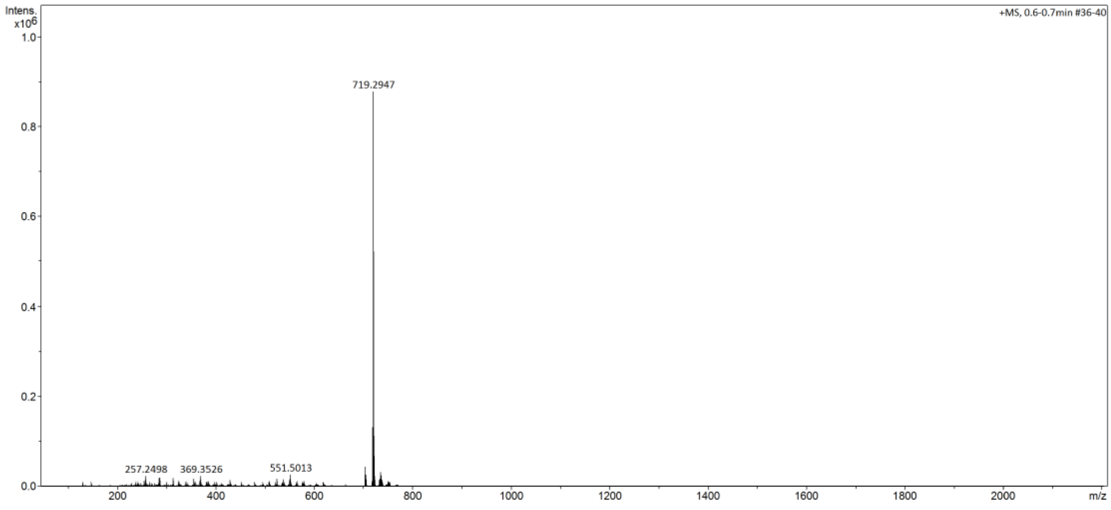

Bruker Compass DataAnalysis 4.3 | Analysis Name EC_4046_a.d | 18/05/2022 15:20:28 | 1 of 1

Figure **S12**. HRMS analysis report for **CNPh-DMAC-TRZ**.

Elemental Analysis Service Request Form

Researcher name Ettore Crovini

Researcher email ec254@st-andrews.ac.uk

NOTE: Please submit ca. 10 mg of sample

**Sample reference number**	EC-4046
**Name of Compound**	**dCNPh-DMAC-TRZ**
**Molecular formula**	$C_{50}H_{34}N_6$
**Stability**	Air stable
**Hazards**	None
**Other Remarks**	None

Analysis type: Elemental Analysis

Single ☐ **Duplicate** ☑ **Triplicate** ☐

Analysis Result:

Element	Expected %	Found (1)	Found (2)	Found (3)
Carbon	83.54	82.44	82.63	
Hydrogen	4.77	4.68	4.78	
Nitrogen	11.69	11.51	11.55	

Authorising Signature:

**Date completed**	23.05.22
**Signature**	[illegible]
**comments**	

Figure **S13**. EA analysis report for **CNPh-DMAC-TRZ**.

10-(4-(4,6-diphenyl-1,3,5-triazin-2-yl)phenyl)-9,9-dimethyl-2,7-bis(4-(trifluoromethyl)phenyl)-9,10-dihydroacridine (CF_3Ph-DMAC-TRZ)

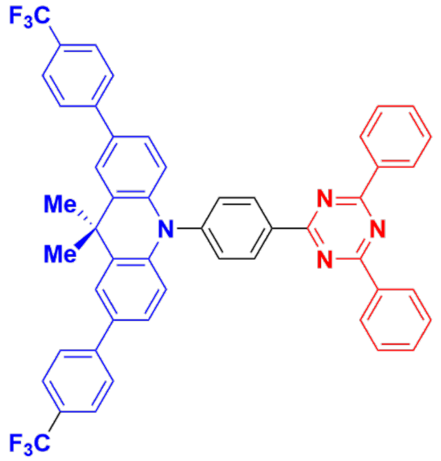

The same reaction procedure as **^{t}BuPh-DMAC-TRZ** was followed but with a different boronic acid, 4-trifluoromethyl-phenylboronic acid (1.126 g. 5.931 mmol, 4 equiv.). After 72 h, the reaction was diluted with THF and filtered over celite to remove any unreacted palladium. The solution was diluted with water, and the organic layer was extracted with DCM (3 × 50 mL). The organic phase was washed with brine, dried over $MgSO_4$ and concentrated under reduced pressure. The target product was isolated as yellow solid after purification by column chromatography on silica gel using 5:1 hexane/EtOAc as eluent. It was then further purified *via* recrystallization in THF/EtOH. (0.6706 g). The compound was further purified by gradient temperature sublimation. **Yield:** 56%; **Mp**: 320-325 °C; **R_f:** 0.49 (ethyl acetate/cyclohexane = 1:9); **^{1}H NMR** (500 MHz, $CDCl_3$) δ: 9.10-9.08 (d, *J*=8.5, 2H), 8.85-8,83 (dd, *J*=8.2, 1.4, 4H), 7.76-7.75 (d, *J*=2.1, 2H), 7.68 – 7.61 (m, 16H), 7.28 (d, *J*=2.1, 1H), 6.50-6.49 (d, *J*=8.6, 2H), 1.87 (s, 6H); **^{13}C NMR** (126 MHz, $CDCl_3$) δ: 172.08, 171.08, 144.74, 144.66, 140.50, 136.83, 136.13, 132.96, 132.66, 131.98, 131.51, 130.65, 129.18, 128.92, 126.87, 125.87, 125.84, 125.69, 125.61, 124.74, 115.01, 36.54, 32.04; **HPLC retention time:** 10.185 min; **purity:** 100.00%; **HR-MS** (APCI) $[M+H]^+$ **Calculated:** ($C_{50}H_{35}F_6N_4^+$) 805.2760, **Found:** 805.2798; **EA** ($C_{50}H_{34}F_6N_4$) **Calculated** C: 74.62, H: 4.26, N: 6.96; **Found** C: 74.88, H: 4.30, N: 6.89.

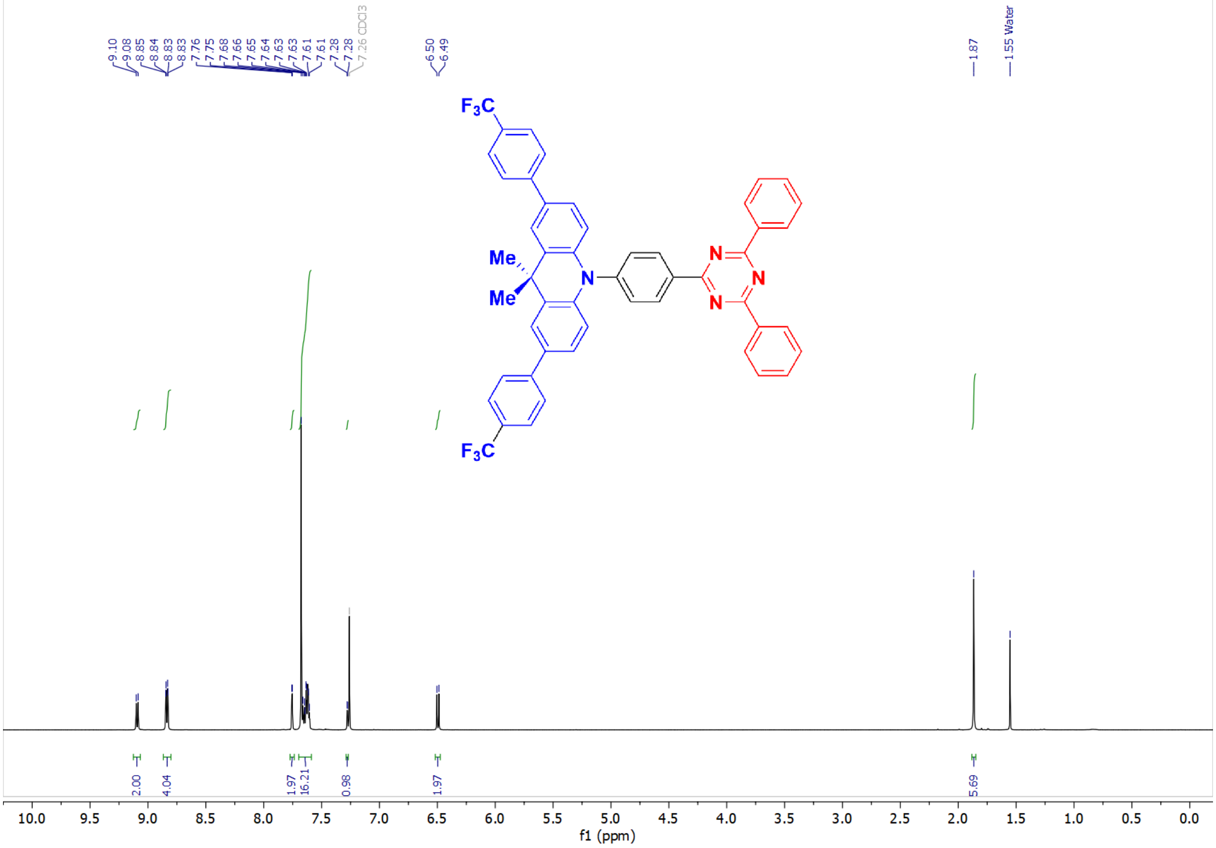

Figure **S14**. ^{1}H NMR of **CF_3Ph-DMAC-TRZ** in $CDCl_3$.

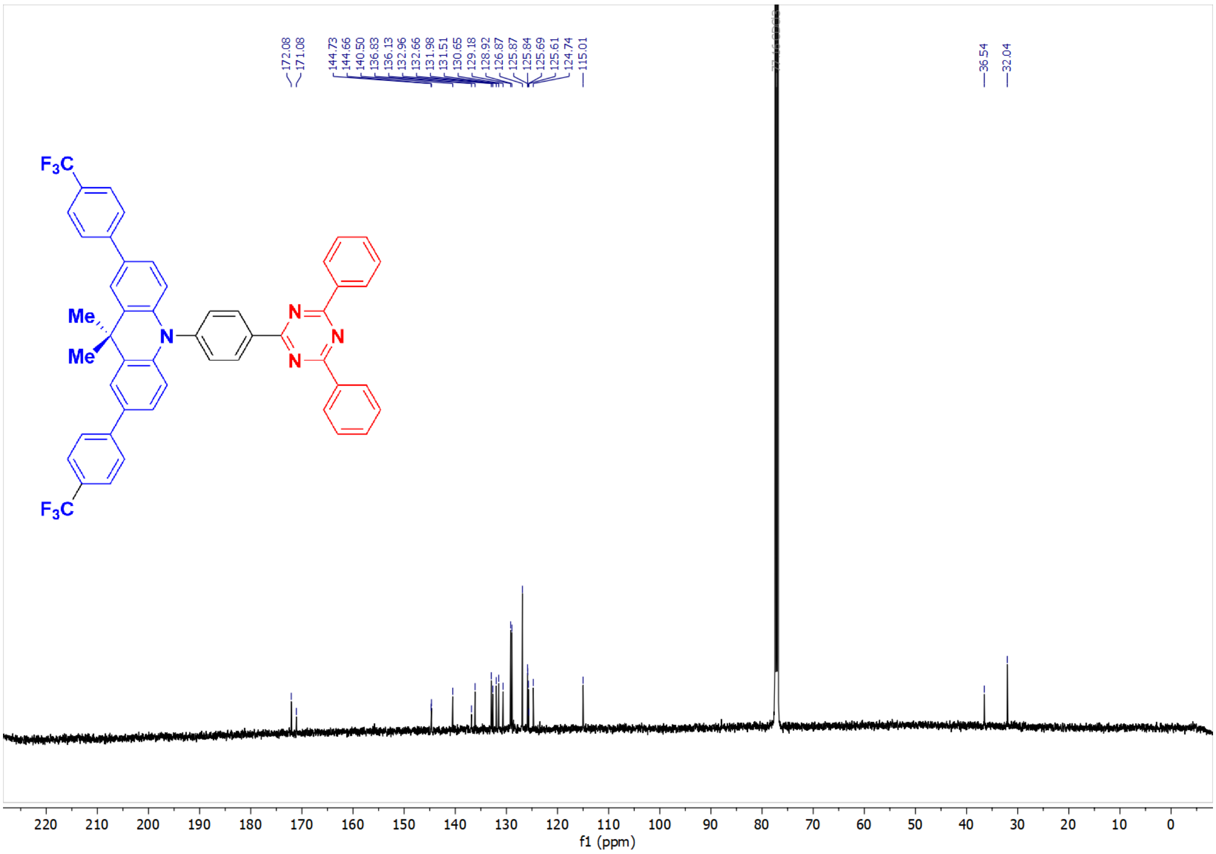

Figure **S15**. ^{13}C NMR of **CF_3Ph-DMAC-TRZ** in $CDCl_3$.

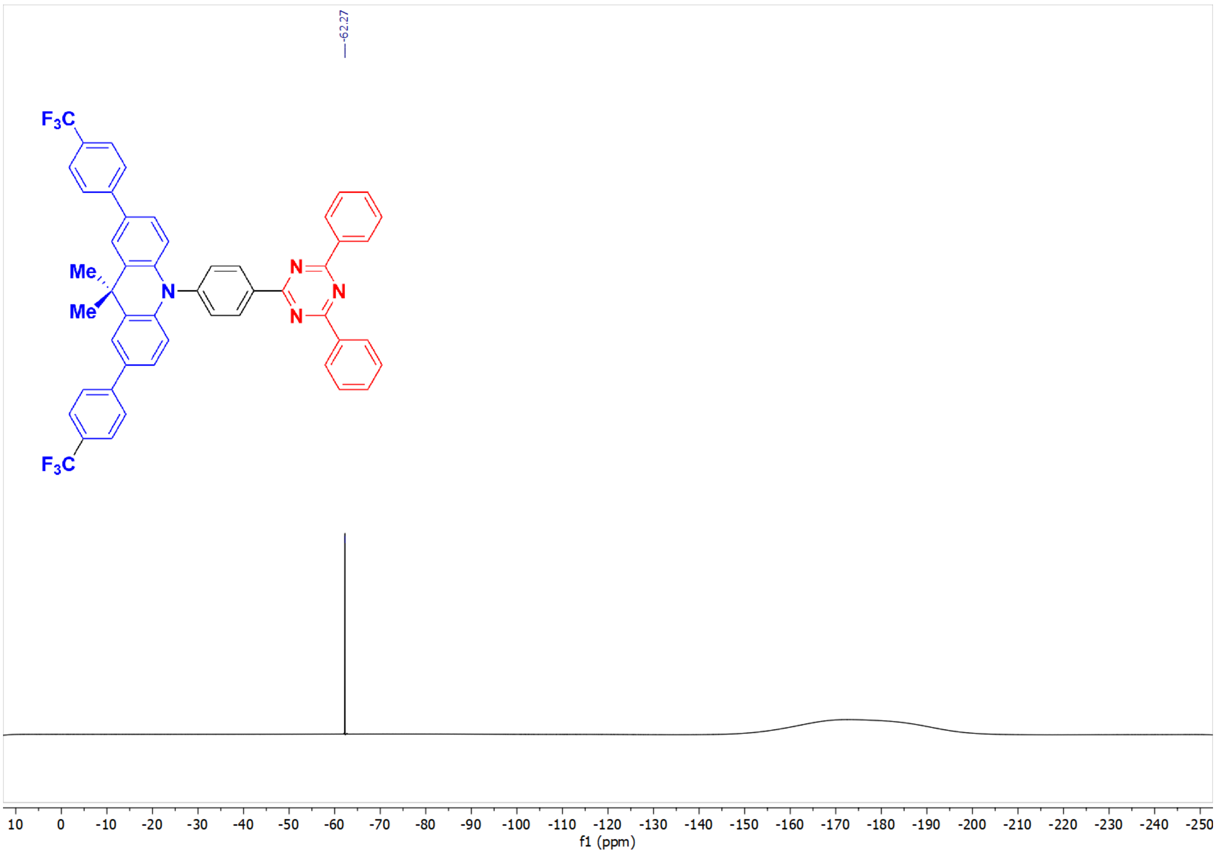

Figure **S16**. ^{19}F NMR of **CF_3Ph-DMAC-TRZ** in $CDCl_3$.

HPLC Trace Report24Mar2022

<Sample Information>

Sample Name	: dCF3PhDMAC-TRZ-S2		
Sample ID	:		
Method Filename	: 100% THF 20 mins 280nm - DH.lcm		
Batch Filename	: 23-03-22.lcb		
Vial #	: 1-3	Sample Type	: Unknown
Injection Volume	: 1 uL		
Date Acquired	: 23/03/2022 17:59:17	Acquired by	: System Administrator
Date Processed	: 23/03/2022 18:19:19	Processed by	: System Administrator

<Chromatogram>

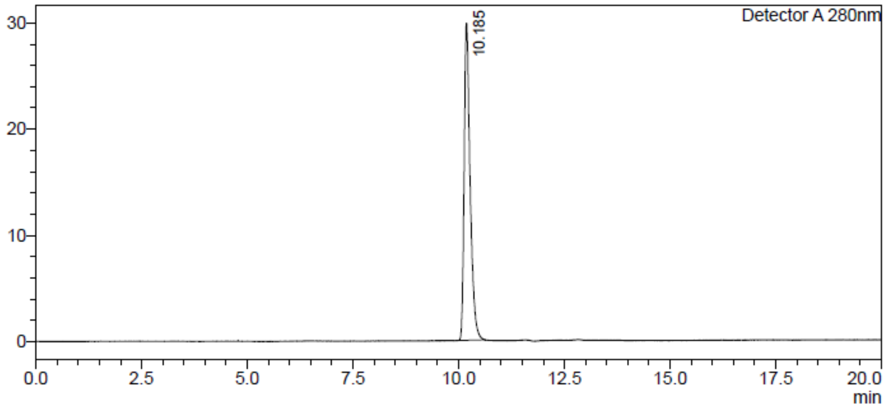

<Peak Table>

Detector A 280nm

Peak#	Ret. Time	Area	Height	Area%	Area/Height	Width at 5% Height
1	10.185	292613	29803	100.000	9.818	0.353
Total		292613	29803	100.000		

Figure **S17**. HPLC analysis report for **CF_3Ph-DMAC-TRZ**.

School of Chemistry Mass Spectrometry Service

SampleID				Submitter			
Sample Description							
Analysis Name	D:\Data\stuartwarriner\manual\EC_3620_a.d			Supervisor			
Method	DIP Pos 3.m			Acquisition Date		18/05/2022 14:06:33	
Instrument	maXis impact	Source Type APCI	Ion Polarity Positive	Scan Begin	50 m/z	Scan End	2200 m/z

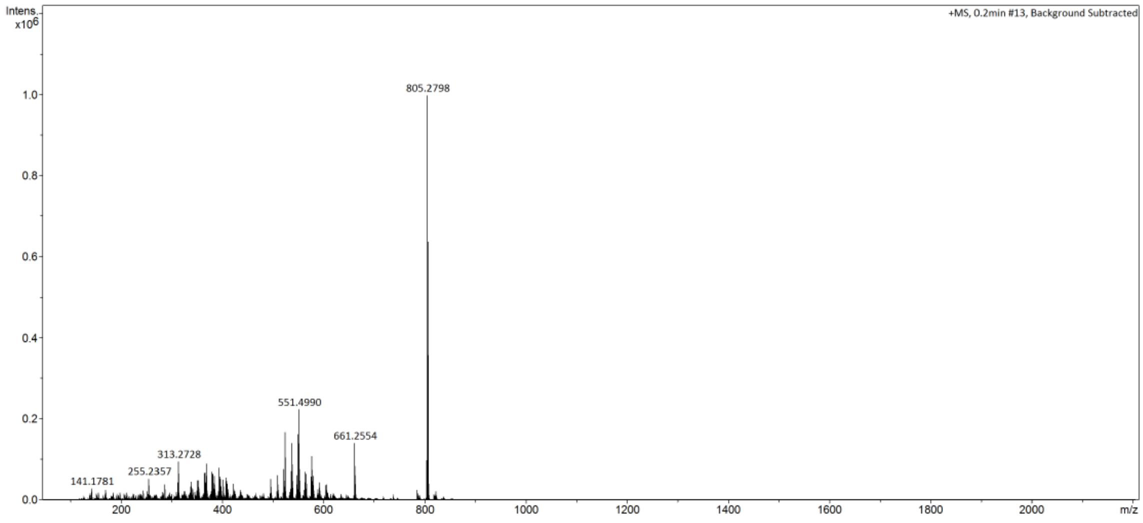

Bruker Compass DataAnalysis 4.3 | Analysis Name | EC_3620_a.d | 18/05/2022 14:10:37 | 1 of 1

Figure **S18**. HRMS analysis report for **CF_3Ph-DMAC-TRZ**.

Elemental Analysis Service Request Form

Researcher name Ettore Crovini

Researcher email ec254@st-andrews.ac.uk

NOTE: Please submit ca. 10 mg of sample

**Sample reference number**	EC-3620
**Name of Compound**	dCF₃Ph-DMAC-TRZ
**Molecular formula**	$C_{50}H_{34}F_6N_4$
**Stability**	Air stable
**Hazards**	None
**Other Remarks**	None

Analysis type: Elemental Analysis

Single ☐ **Duplicate** ☒ **Triplicate** ☐

Analysis Result:

Element	Expected %	Found (1)	Found (2)	Found (3)
Carbon	74.62	74.99	74.76	
Hydrogen	4.26	4.30	4.31	
Nitrogen	6.96	6.90	6.88	
Fluorine	14.16			

Authorising Signature:

**Date completed**	29.04.22
**Signature**	J -PL
**comments**	

Figure **S19**. EA analysis report for **CF_3Ph-DMAC-TRZ**.

2,7-di([1,1'-biphenyl]-4-yl)-10-(4-(4,6-diphenyl-1,3,5-triazin-2-yl)phenyl)-9,9-dimethyl-9,10-dihydroacridine (dPh-DMAC-TRZ)

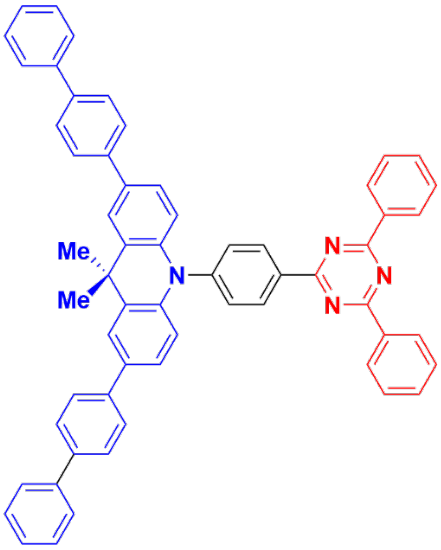

The same reaction procedure as **^{t}BuPh-DMAC-TRZ** was followed but with a different boronic acid, [1,1'-biphenyl]-4-ylboronic acid (1.174 g, 5.931 mmol, 4 equiv.). After 72 h, the reaction was diluted with THF and filtered over celite to remove any unreacted palladium. The solution was diluted with water, and the organic layer was extracted with DCM (3 × 50 mL). The organic phase was washed with brine, dried over $MgSO_4$ and concentrated under reduced pressure. The target product was isolated as yellow solid after purification by column chromatography on silica gel using 5:1 hexane/EtOAc as eluent. It was then further purified *via* recrystallization in THF/MeOH. (0.650 g). The compound was further purified by gradient temperature sublimation. **Yield:** 53%; **Mp**:330-335 °C ; **R*f*:** 0.67 (ethyl acetate/cyclohexane = 1:4); **^{1}H NMR** (500 MHz, $CDCl_3$) δ: 9.10-9.08 (dt, *J*= 8.4, 2.1, 1.4, 2H), 8.85-8.83 (dt, *J*= 6.7, 1.6, 1.0, 2H), 7.81-7.80 (d, *J*= 2.1, 4H), 7.67-7.70 (m, 20H), 7.48 – 7.45 (t, *J*= 7.7, 7.7, 4H), 7.37-7.34 (t, *J*= 7.4, 7.4, 2H), 7.31-7.29 (dd, *J*= 8.6, 2.1, 2H), 6.50-6.48 (d, *J*= 8.6, 2H), 1.89 (s, 6H); **^{13}C NMR** (126 MHz, $CDCl_3$) δ: 172.05, 171.18, 140.97, 140.24, 140.02, 139.57, 136.57, 136.19, 133.51, 132.91, 131.90, 131.65, 130.60, 129.19, 128.95, 128.90, 127.64, 127.36, 127.15, 127.08, 125.36, 124.56, 114.83, 36.57, 32.09; **HPLC retention time:** 10.326; **purity:** 100.00%; **HR-MS** (APCI) $[M+H]^+$ **Calculated:** ($C_{60}H_{45}N_4^+$) 821.3639, **Found:** 821.3653; **EA** ($C_{60}H_{44}N_4$) **Calculated** C: 87.77, H: 5.40, N: 6.82; **Found** C: 87.36, H: 5.41, N: 6.79.

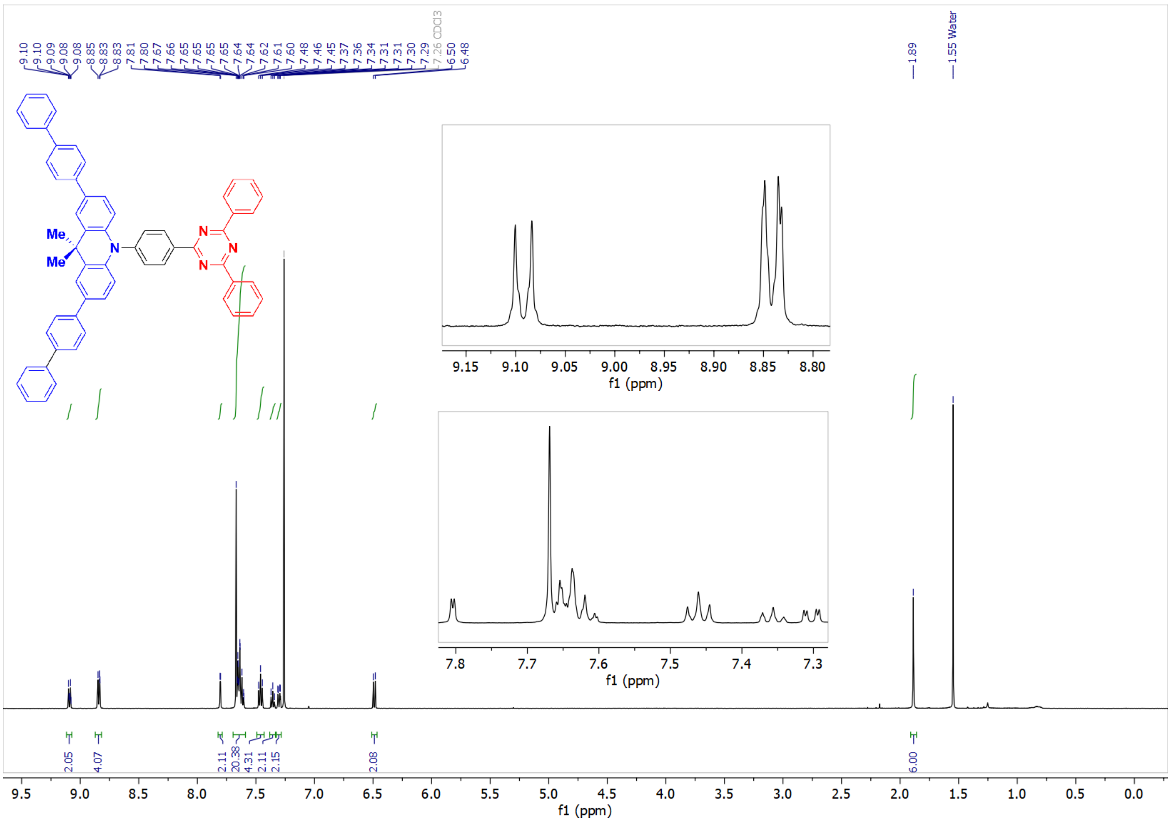

Figure **S20**. ^{1}H NMR of **dPh-DMAC-TRZ** in $CDCl_3$.

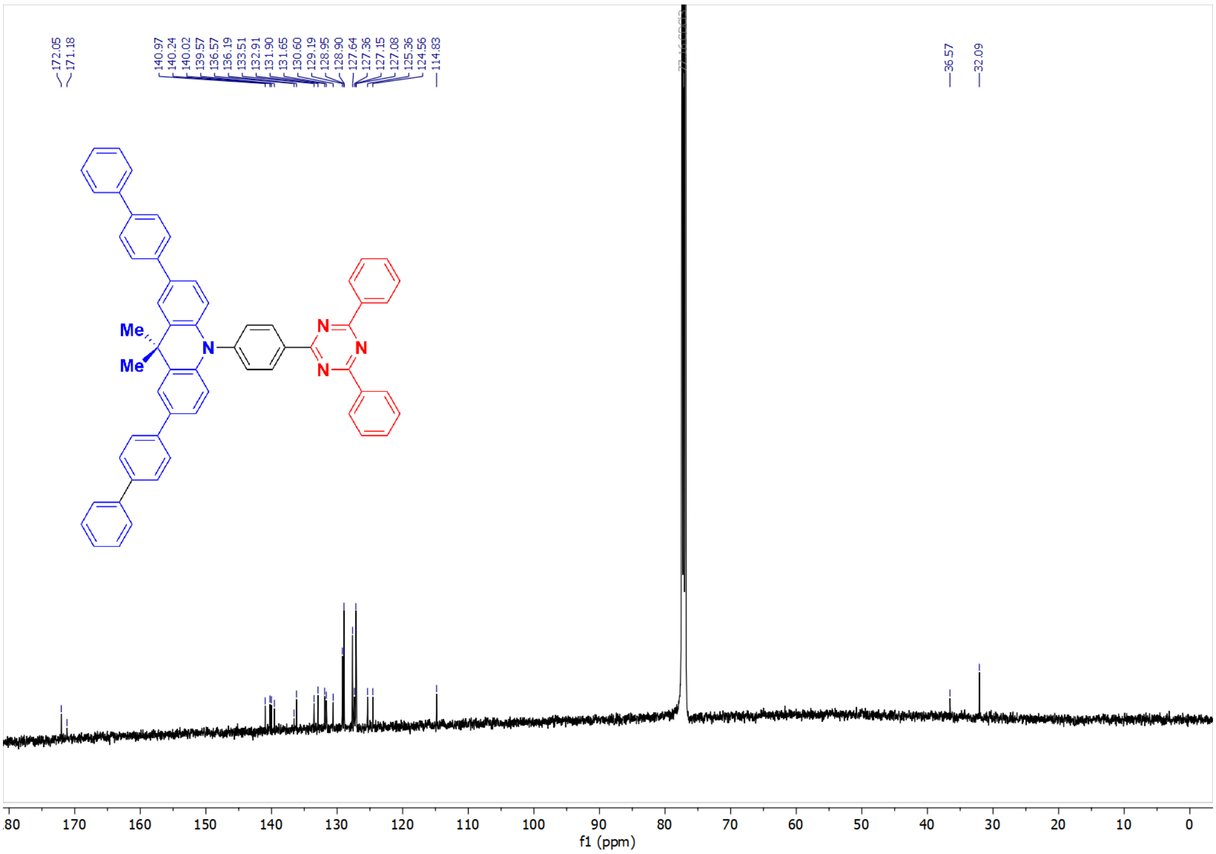

Figure **S21**. ^{13}C NMR of **dPh-DMAC-TRZ** in $CDCl_3$.

HPLC Trace Report06Apr2022

<Sample Information>

Sample Name	: EC-4044-dP-DMAC-TRZ
Sample ID	:
Method Filename	: 100% THF 20 mins 280nm - DH.lcm
Batch Filename	: 06-04-22.lcb
Vial #	: 1-47
Sample Type	: Unknown
Injection Volume	: 10 uL
Date Acquired	: 06/04/2022 11:55:36
Acquired by	: System Administrator
Date Processed	: 06/04/2022 12:15:39
Processed by	: System Administrator

<Chromatogram>

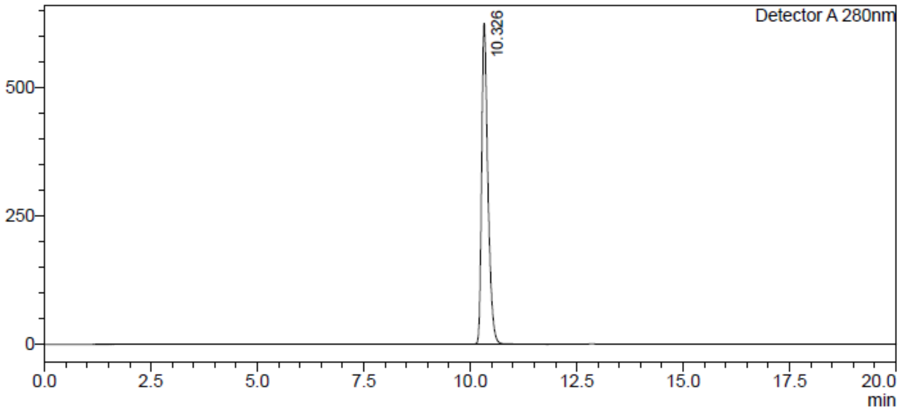

<Peak Table>

Detector A 280nm

Peak#	Ret. Time	Area	Height	Area%	Area/Height	Width at 5% Height
1	10.326	6401268	622597	100.000	10.282	0.364
Total		6401268	622597	100.000		

Figure **S22**. HPLC analysis report for **dPh-DMAC-TRZ**.

School of Chemistry Mass Spectrometry Service

**SampleID**				**Submitter**	
**Sample Description**					
**Analysis Name**	D:\Data\stuartwarriner\manual\EC_4044_a.d			**Supervisor**	
**Method**	DIP Pos 3.m			**Acquisition Date**	18/05/2022 15:08:34
**Instrument**	maXis impact	**Source Type** APCI	**Ion Polarity** Positive	**Scan Begin** 50 m/z	**Scan End** 2200 m/z

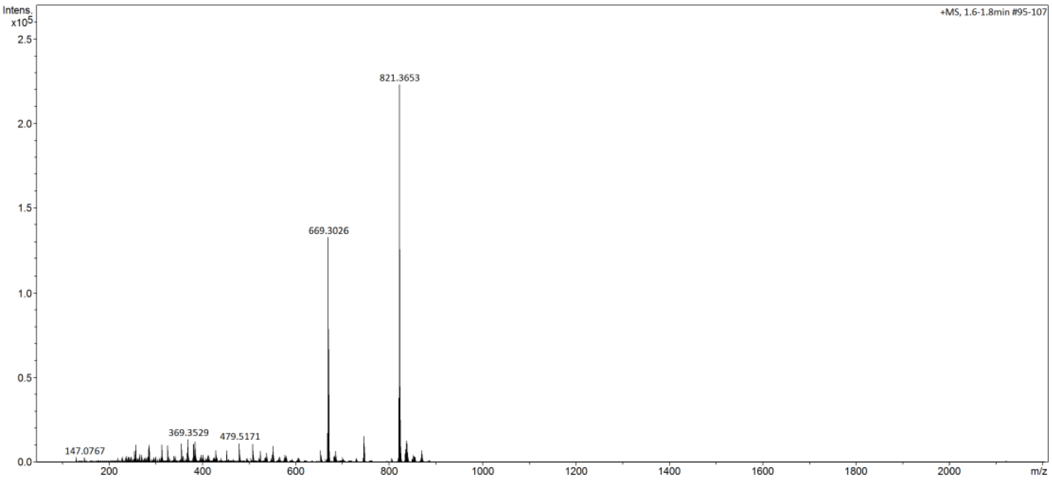

Bruker Compass DataAnalysis 4.3 Analysis Name EC_4044_a.d 18/05/2022 15:13:45 1 of 1

Figure **S23**. HRMS analysis report for **dPh-DMAC-TRZ**.

Elemental Analysis Service Request Form

Researcher name Ettore Crovini

Researcher email ec254@st-andrews.ac.uk

NOTE: Please submit ca. 10 mg of sample

**Sample reference number**	EC-4044
**Name of Compound**	**dPh-DMAC-TRZ**
**Molecular formula**	$C_{60}H_{44}N_4$
**Stability**	Air stable
**Hazards**	None
**Other Remarks**	None

Analysis type: Elemental analysis

Single ☐ **Duplicate** ☒ **Triplicate** ☐

Analysis Result:

Element	Expected %	Found (1)	Found (2)	Found (3)
Carbon	87.77	87.93	86.79	
Hydrogen	5.40	5.47	5.35	
Nitrogen	6.82	6.84	6.74	

Authorising Signature:

**Date completed**	29.04.22
**Signature**	J-PC
**comments**	

Figure **S24**. EA analysis report for **dPh-DMAC-TRZ**.

2,7-bis(4-(tert-butyl)phenyl)-10-(4-(4,6-diphenyl-1,3,5-triazin-2-yl)phenyl)-9,9-dimethyl-9,10-dihydroacridine (*t*BuPh-DMAC-TRZ)

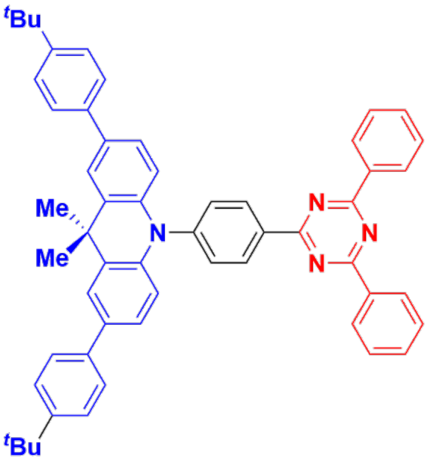

To a pressure vessel were added 2,7-dibromo-10-(4-(4,6-diphenyl-1,3,5-triazin-2-yl)phenyl)-9,9-dimethyl-9,10-dihydroacridine (**dBr-DMAC-TRZ**) (1.0 g, 1.48 mmol, 1 equiv.) and 4-*tert*-butylphenyl-boronic acid (1.1 g, 5.93 mmol, 4 equiv.) and the vessel was degassed by alternating vacuum and nitrogen 3 times. THF (22 mL), and 16.5 mL of a 2 M Cs_2CO_3 solution in water were added and the bi-phasic solution was degassed by bubbling N_2 trough for 30 min. $Pd(PPh_3)_4$ was then added and the reaction was left to stir under pressure at 110°C for 72h. A yellow crystalline solid crashed out of the solution. The mixture was left to cool, and the yellow solid was filtered and washed with water (250 mL) and MeOH (250 mL) to achieve the desired product (0.73 g). The compound was further purified by gradient temperature sublimation **Yield:** 63%; **Mp**: 325-330 °C; **R_f:** 0.50 (ethyl acetate/cyclohexane = 1 :9); **^{1}H NMR** (300 MHz, $CDCl_3$) δ: 9.08-9.06 (d, *J*= 7.1, 2H),, 8.84-8.82 (d, *J*= 4.2, 4H), 7.73 (d, *J*= 2.0, 2H), 7.67-, 7.60 (m, 8H), 7.53-7.45 (dd, *J*= 22.3, 8.84, 8H), 7.24-7.22 (dd, *J*= 8.55, 2.05, 2H), 6.46-6.44 (d, *J*= 8.5, 2H), 1.83 (s, 6H), 1.36 (s,18H); **^{13}C NMR** (126 MHz, $CDCl_3$) δ = 172.02, 171.20, 149.65, 145.33, 139.81, 138.54, 136.41, 136.20, 133.92, 132.88, 131.83, 131.67, 130.47, 129.18, 128.89, 126.44, 125.81, 125.28, 124.55, 114.67, 36.52, 34.63, 31.91, 31.54; **HPLC retention time:** 10.239 min; **purity:** >99%; **HR-MS** (APCI) $[M+H]^+$ **Calculated:** ($C_{56}H_{53}N_4^+$) 781.4268, **Found:** 781.4268; **EA** ($C_{56}H_{52}N_4$) **Calculated** C: 86.12, H: 6.71, N: 7.17; **Found** C: 86.51, H: 6.76, N: 7.15.

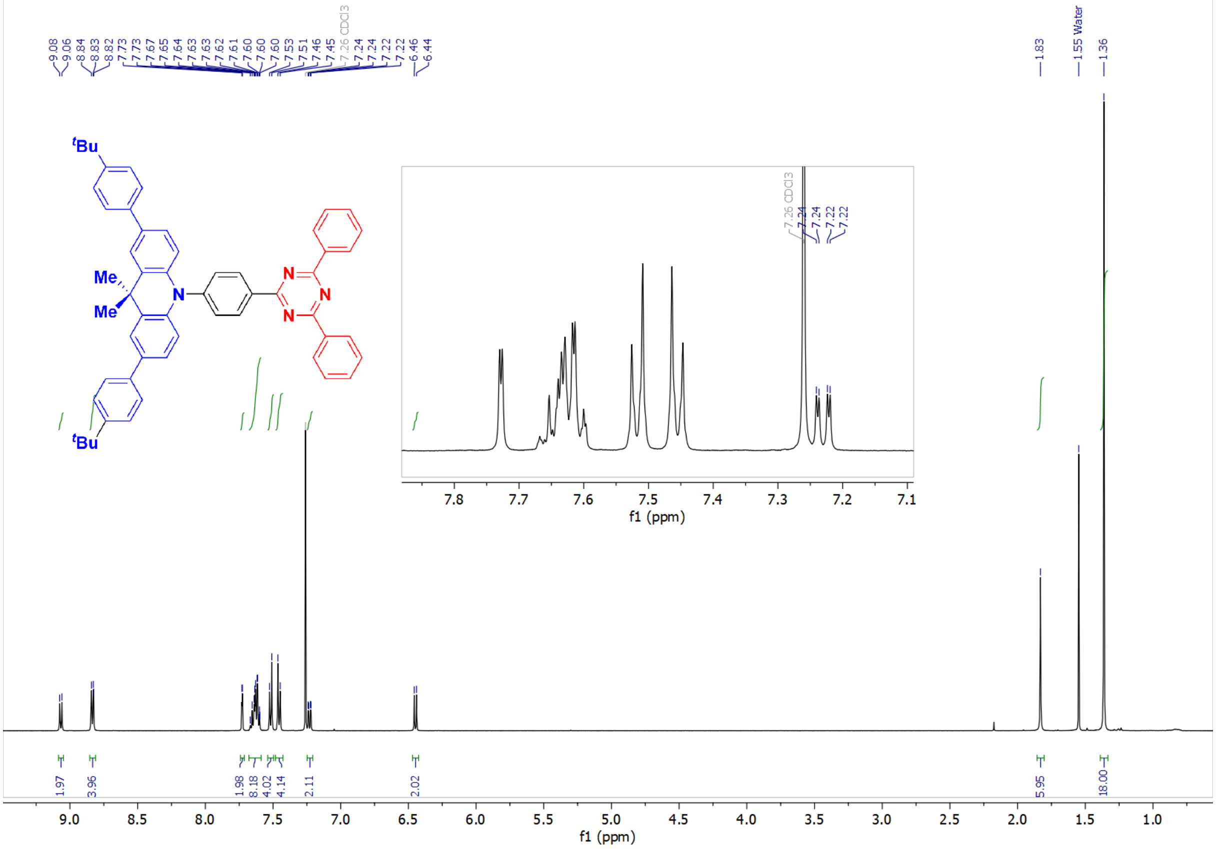

Figure **S25**. ^{1}H NMR of ***t*BuPh-DMAC-TRZ** in $CDCl_3$.

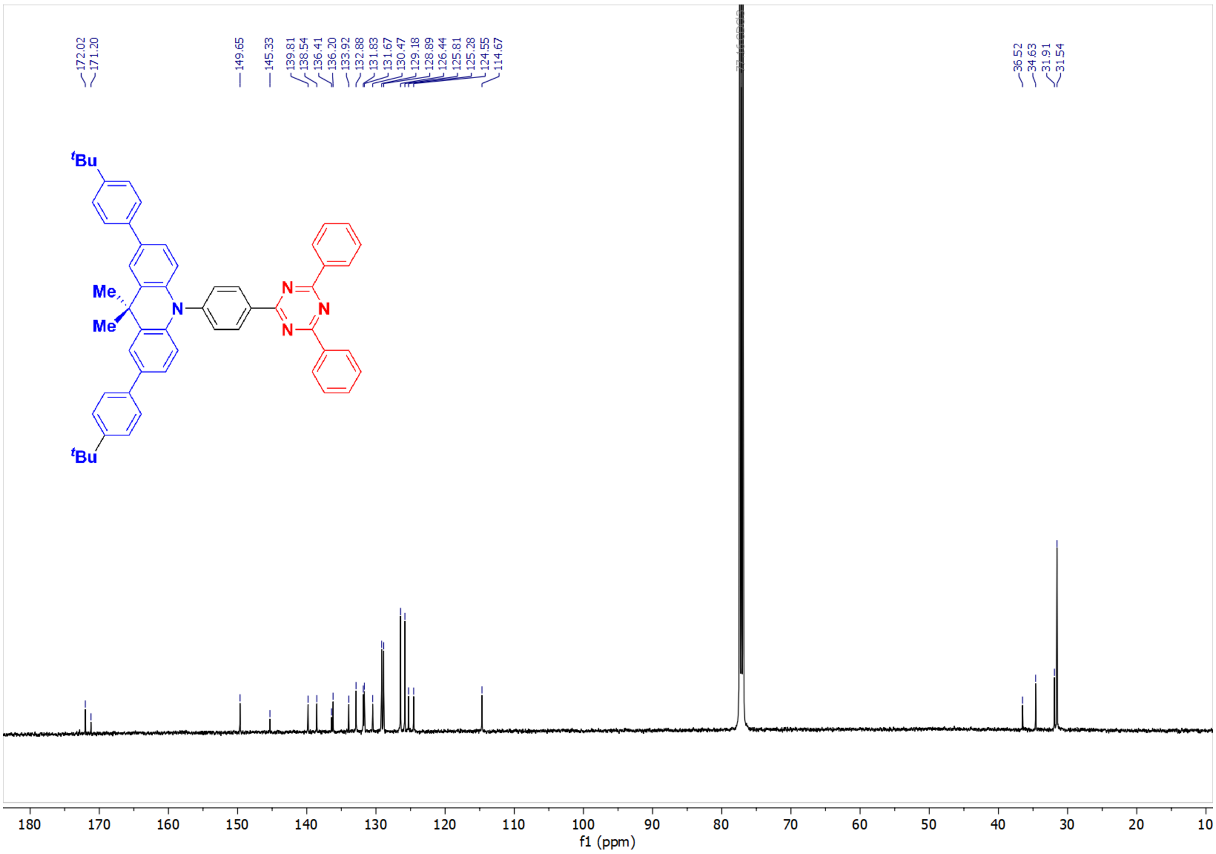

Figure **S26**. ^{13}C NMR of ***t*BuPh-DMAC-TRZ** in $CDCl_3$.

HPLC Trace Report24Mar2022

<Sample Information>

Sample Name	: dtBuPhDMAC-TRZ-S1		
Sample ID	:		
Method Filename	: 100% THF 20 mins 280nm - DH.lcm		
Batch Filename	: 23-03-22.lcb		
Vial #	: 1-2	Sample Type	: Unknown
Injection Volume	: 10 uL		
Date Acquired	: 23/03/2022 17:18:29	Acquired by	: System Administrator
Date Processed	: 23/03/2022 17:38:33	Processed by	: System Administrator

<Chromatogram>

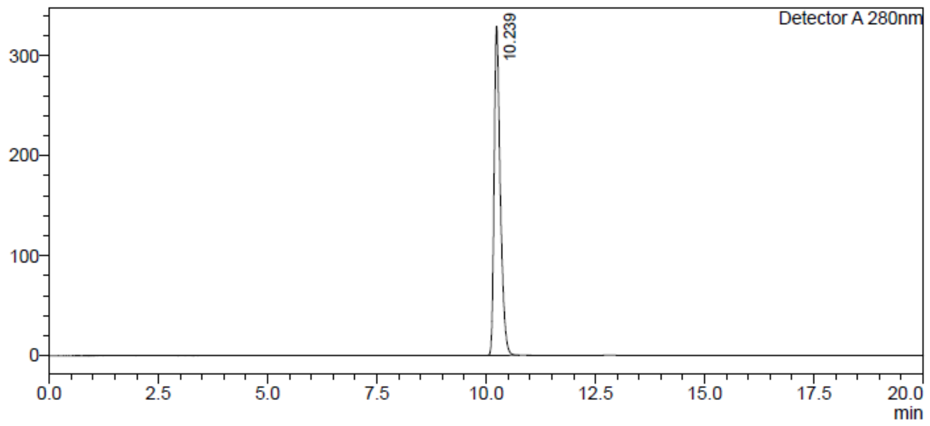

<Peak Table>

Detector A 280nm

Peak#	Ret. Time	Area	Height	Area%	Area/Height	Width at 5% Height
1	10.239	3232827	328784	100.000	9.833	0.349
Total		3232827	328784	100.000		

Figure **S27**. HPLC analysis report for **^{t}BuPh-DMAC-TRZ**.

School of Chemistry Mass Spectrometry Service

SampleID				Submitter			
Sample Description							
Analysis Name	D:\Data\stuartwarriner\manual\dtBuPh_DMAC_TRZ_b.d			Supervisor			
Method	DIP Pos 3.m			Acquisition Date	12/03/2022 17:48:23		
Instrument	maXis impact	Source Type	APCI	Ion Polarity	Positive	Scan Begin 50 m/z	Scan End 2200 m/z

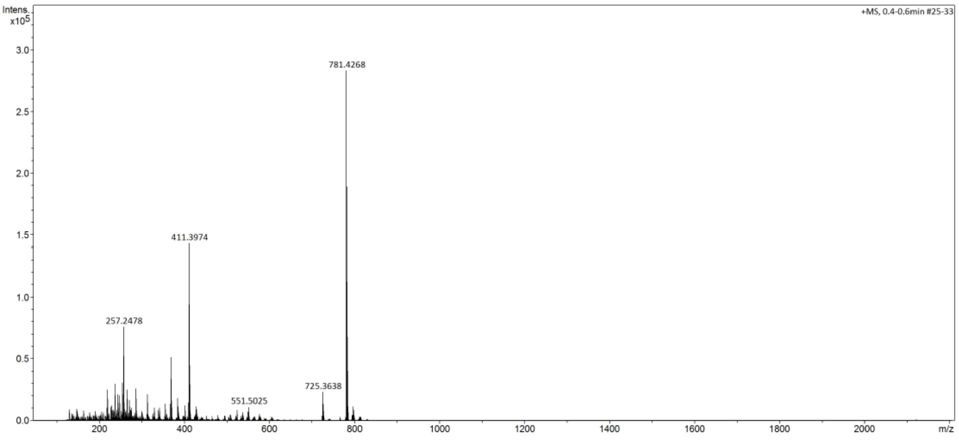

Bruker Compass DataAnalysis 4.3 Analysis Name dtBuPh_DMAC_TRZ_b.d 12/03/2022 17:50:34 1 of 1

Figure **S28**. HRMS analysis report for **^{t}BuPh-DMAC-TRZ**.

Elemental Analysis Service Request Form

Researcher name Ettore Crovini

Researcher email ec254@st-andrews.ac.uk

NOTE: Please submit ca. 10 mg of sample

**Sample reference number**	EC-3621
**Name of Compound**	d^{t}BuPh-DMAC-TRZ
**Molecular formula**	$C_{56}H_{52}N_4$
**Stability**	Air stable
**Hazards**	No hazards
**Other Remarks**	None

Analysis type: Elemental analysis

Single ☐ **Duplicate** ☒ **Triplicate** ☐

Analysis Result:

Element	Expected %	Found (1)	Found (2)	Found (3)
Carbon	86.12	86.77	86.25	
Hydrogen	6.71	6.75	6.77	
Nitrogen	7.17	7.15	7.16	

Authorising Signature:

**Date completed**	29.04.22
**Signature**	J-PC
**comments**	

Figure **S29**. EA analysis report for ^{t}**BuPh-DMAC-TRZ**.

10-(4-(4,6-diphenyl-1,3,5-triazin-2-yl)phenyl)-2,7-bis(4-methoxyphenyl)-9,9-dimethyl-9,10-dihydroacridine (OMePh-DMAC-TRZ)

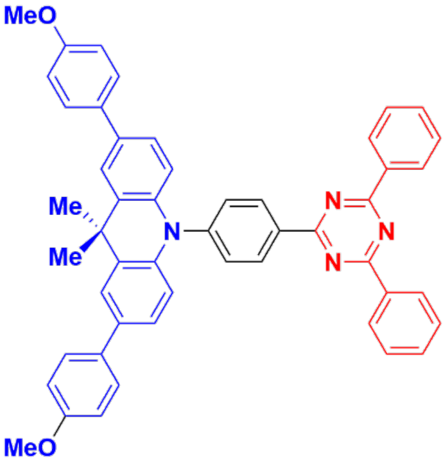

The same reaction procedure as ***t*BuPh-DMAC-TRZ** was followed, but with a different boronic acid, 4-methoxy-phenyl-boronic acid (0.901 g, 5.931 mmol, 4 equiv.). After 72 h, the reaction was diluted with THF and filtered over celite to remove any unreacted palladium. The solution was diluted with water, and the organic layer was extracted with DCM (3 × 50 mL). The organic phase was washed with brine, dried over $MgSO_4$ and concentrated under reduced pressure. The target product was isolated as yellow solid after purification by column chromatography on silica gel using 5:1 hexane/EtOAc as eluent. It was then further purified *via* recrystallization in THF/Ether. (0.502 g). The compound was furtherly purified by gradient temperature sublimation. **Yield:** 47%; **Mp**: 300-305 °C; **R_f:** 0.39 (ethyl acetate/cyclohexane= 1:9); **^{1}H NMR** (500 MHz, $CDCl_3$) δ: 9.08-9.05 (dt, *J*= 8.4, 2.3, 1.7, 2H), 8.84 – 8.82 (dt, *J*= 7.0, 1.6, 1.6 4H), 7.69 (d, *J*= 2.1, 2H), 7.67-7.60 (m, 8H) 7.52-7.49 (dt, *J*= 8.5, 2.6, 2.0, 4H), 7.20-7.18 (dd, *J*= 8.56, 2.08, 2H), 6.99-6.96 (dt, *J*= 8.7, 3.0, 1.5, 4H), 6.45-6.43 (d, *J*= 8.5, 2H), 3.85 (s, 6H), 1.84 (s, 6H); **^{13}C NMR** (126 MHz, $CDCl_3$) δ: 172.02, 171.20, 158.77, 145.37, 139.56, 136.38, 136.19, 134.02, 133.68, 132.88, 131.83, 131.62, 130.53, 129.18, 128.89, 127.77, 125.02, 124.22, 114.71, 114.32, 55.52, 36.52, 31.98; **HPLC retention time:** 10.444 min; **purity:** 100.00%; **HR-MS** (APCI) $[M+H]^+$ **Calculated:** ($C_{50}H_{41}N_4O_2^+$) 729.3224, **Found:** 729.3253; **EA** ($C_{50}H_{40}N_4O_2$) **Calculated** C: 82.39, H: 5.53, N: 7.69; **Found** C: 82.59, H: 5.57, N: 7.62.

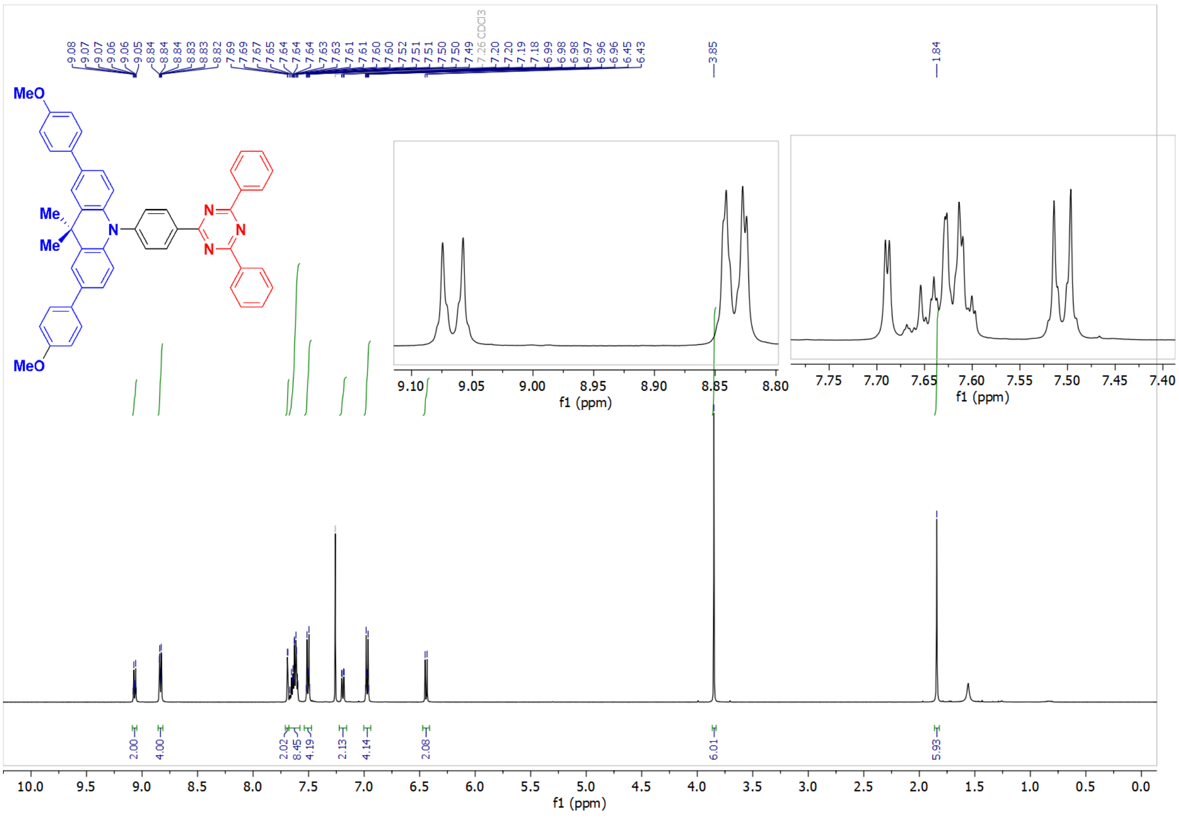

Figure **S30**. ^{1}H NMR of **OMePh-DMAC-TRZ** in $CDCl_3$.

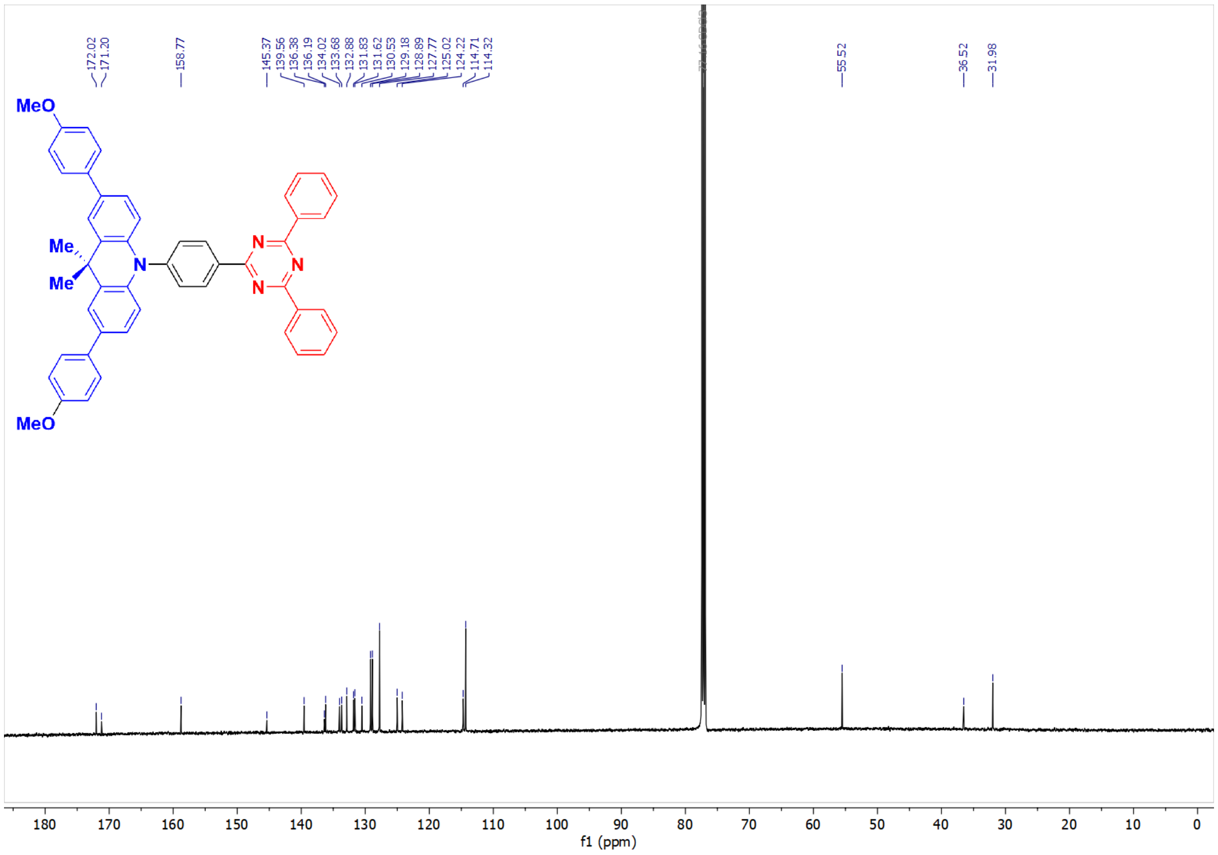

Figure **S31**. ^{13}C NMR of **OMePh-DMAC-TRZ** in $CDCl_3$.

HPLC Trace Report24Mar2022

<Sample Information>

Sample Name	: dOMePhDMAC-TRZ-S1		
Sample ID	:		
Method Filename	: 100% THF 20 mins 280nm - DH.lcm		
Batch Filename	: 23-03-22.lcb		
Vial #	: 1-4	Sample Type	: Unknown
Injection Volume	: 1 uL		
Date Acquired	: 23/03/2022 18:40:01	Acquired by	: System Administrator
Date Processed	: 23/03/2022 19:00:04	Processed by	: System Administrator

<Chromatogram>

mV

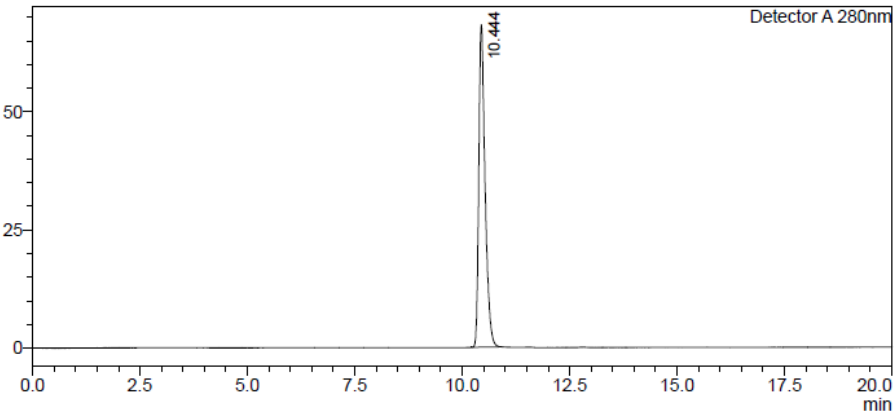

<Peak Table>

Detector A 280nm

Peak#	Ret. Time	Area	Height	Area%	Area/Height	Width at 5% Height
1	10.444	693776	68194	100.000	10.174	0.362
Total		693776	68194	100.000		

Figure **S32**. HPLC analysis report for **OMePh-DMAC-TRZ**.

School of Chemistry Mass Spectrometry Service

**SampleID**				**Submitter**			
**Sample Description**							
**Analysis Name**	D:\Data\stuartwarriner\manual\EC_4043_a.d			**Supervisor**			
**Method**	DIP Pos 3.m			**Acquisition Date**	18/05/2022 14:32:15		
**Instrument**	maXis impact	**Source Type** APCI	**Ion Polarity** Positive	**Scan Begin**	50 m/z	**Scan End**	2200 m/z

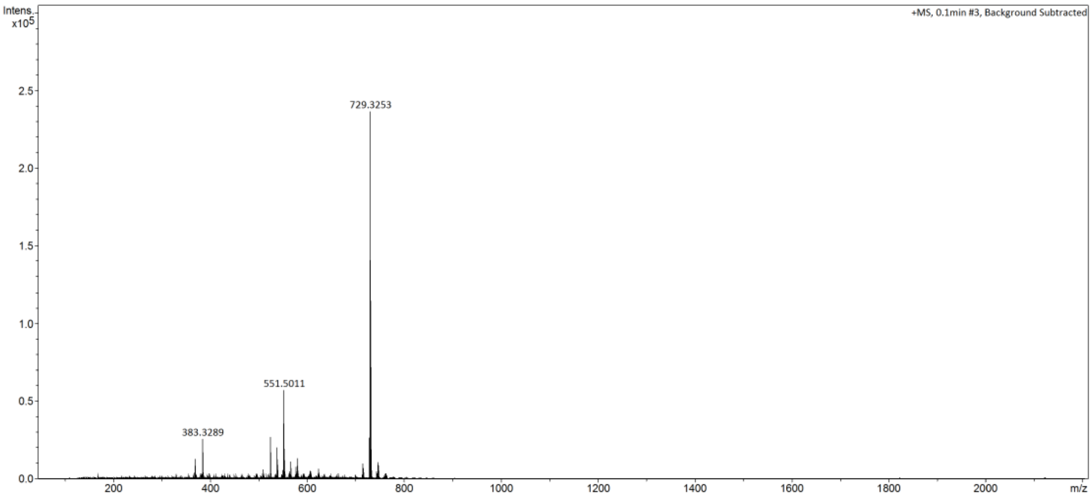

Bruker Compass DataAnalysis 4.3 Analysis Name EC_4043_a.d 18/05/2022 14:38:57 1 of 1

Figure **S33**. HRMS analysis report for **OMePh-DMAC-TRZ**.

Elemental Analysis Service Request Form

Researcher name Ettore Crovini

Researcher email ec254@st-andrews.ac.uk

NOTE: Please submit ca. 10 mg of sample

**Sample reference number**	EC-4043
**Name of Compound**	**dOMePh-DMAC-TRZ**
**Molecular formula**	$C_{50}H_{40}N_4O_2$
**Stability**	Air stable
**Hazards**	None
**Other Remarks**	None

Analysis type: Elemental Analysis

Single ☐ **Duplicate** ☒ **Triplicate** ☐

Analysis Result:

Element	Expected %	Found (1)	Found (2)	Found (3)
Carbon	82.39	82.71	82.46	
Hydrogen	5.53	5.57	5.57	
Nitrogen	7.69	7.62	7.61	
Oxygen	4.39			

Authorising Signature:

**Date completed**	29.04.22
**Signature**	[illegible]
**comments**	

Figure **S34**. EA analysis report for **OMePh-DMAC-TRZ**.

DFT Calculations

Table **S1**. Excited states properties of **DMAC-TRZ** and its derivatives.

Excited State[a]	Energy / eV	Nature[b]	Character of the transition
		**DMAC-TRZ**	
**T_1**	2.56	H→L (97%)	^{3}CT
		H→(L+2) 2%	
**S_1** (f=0.00)	2.57	H→L (97%)	^{1}CT
		H→(L+2) 2%	
		**CNPh-DMAC-TRZ**	
**T_1**	2.76	H→L (97%)	^{3}CT
**S_1** (f=0.00)	2.77	H→L (97%)	^{1}CT
		**CF_3Ph-DMAC-TRZ**	
**T_1**	2.66	H→L (97%)	^{3}CT
**S_1** (f=0.05)	2.67	H→L (97%)	^{1}CT
		**dPh-DMAC-TRZ**	
**T_1**	2.47	H→L (97%)	^{3}CT
**S_1** (f=0.00)	2.48	H→L (97%)	^{1}CT
		**OMePh-DMAC-TRZ**	
**T_1**	2.36	H→L (97%)	^{3}CT
**S_1** (f=0.00)	2.37	H→L (97%)	^{1}CT
		**^{t}BuPh-DMAC-TRZ**	
**T_1**	2.43	H→L (97%)	^{3}CT
**S_1** (f=0.00)	2.43	H→L (97%)	^{1}CT

[a] PBE0/6-31G(d,p); [b] the transitions highlighted in red are the main ones contributing to the excited state character.

Table **S2**. Properties of the excited states involved in the UV-vis absorption transitions of **CNPh-DMAC-TRZ**, **CF_3Ph-DMAC-TRZ**, **dPh-DMAC-TRZ**, **^{t}BuPh-DMAC-TRZ**, **OMePh-DMAC-TRZ**.

**Excited State**^{a}	**Energy / eV; nm**	**Nature**	**Character of the transition**
		**CNPh-DMAC-TRZ**	
S_3 (f=1.15)	3.39; 366	H→(L+2) (98%)	^{1}LE (DMAC)
S_4 (f=0.23)	3.78; 328	H→(L+3) (95%)	^{1}LE (DMAC)
		**CF_3Ph-DMAC-TRZ**	
S_3 (f=1.00)	3.64; 341	H→(L+2) (97%)	^{1}LE (DMAC)
S_5 (f=0.15)	4.09; 303	H→(L+3) (82%)	^{1}LE (DMAC)
		H→(L+6) (9%)	
		**dPh-DMAC-TRZ**	
S_3 (f=1.41)	3.53; 351	H→(L+2) (96%)	^{1}LE (DMAC)
S_5 (f=0.25)	3.93; 315	H→(L+3) (89%)	
		H→(L+4) (4%)	
		**^{t}BuPh-DMAC-TRZ**	
S_4 (f=0.57)	3.74; 331	H→(L+2) (80%)	^{1}LE (DMAC)
		H→(L+4) (16%)	
		**OMePh-DMAC-TRZ**	
S_5 (f=0.25)	3.72; 333	H→(L+3) (77%)	^{1}LE (DMAC)
		H→(L+4) (21%)	

^{a} PBE0/6-31G(d,p); ^{b} the transitions highlighted in red are those involved in the UV transitions.

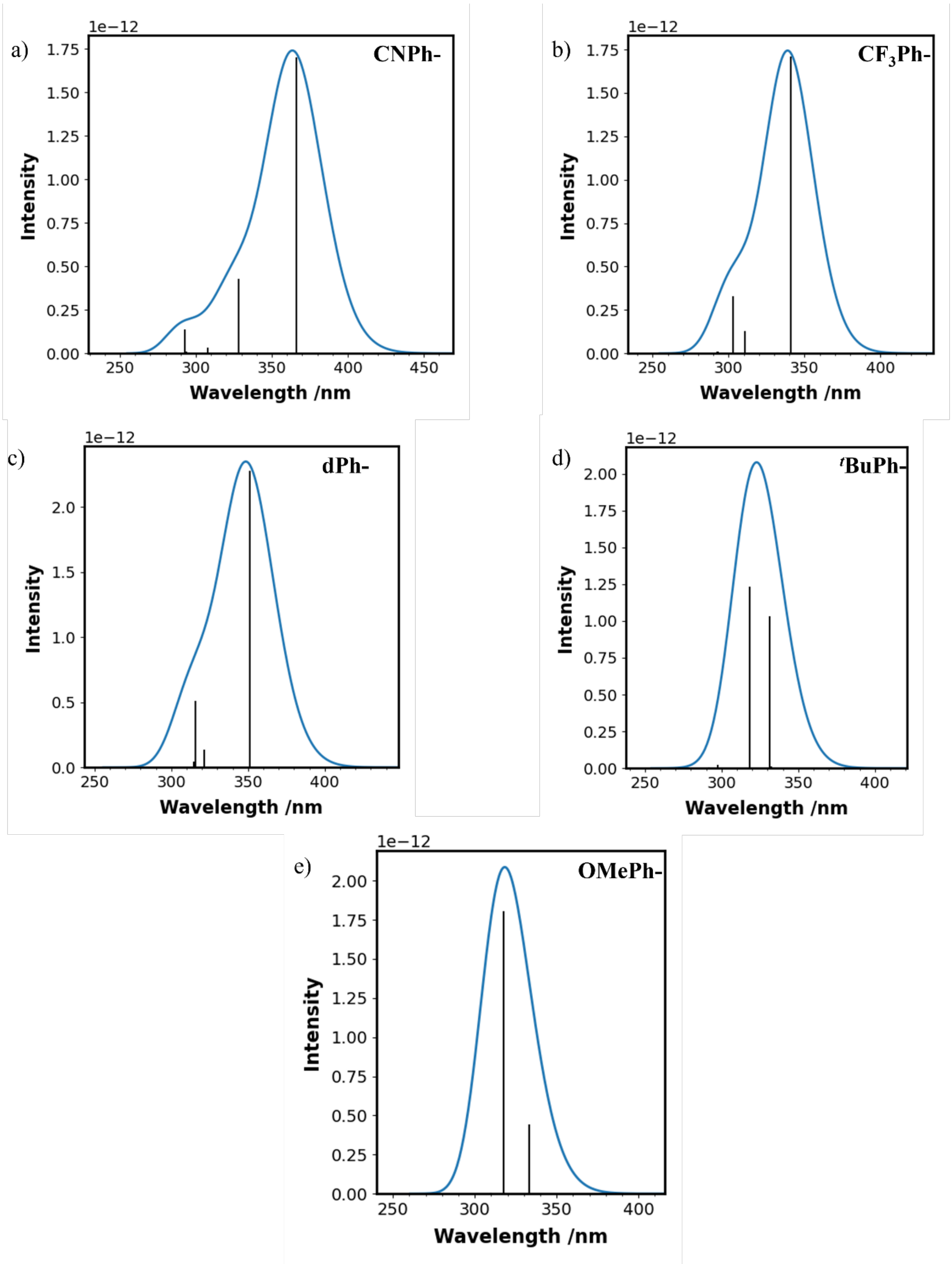

Figure **S35**. Simulated absorption spectra of **DMAC-TRZ** and derivatives (Obtained at the TDA-DFT-PBE0/6-31G(d,p) level, Isovalue for new surfaces: MO=0.02, Density=0.0004).

Table **S3**. Calculated optoelectronic properties of **DMAC-TRZ** and derivatives.

**R-DMAC-TRZ**	**S_1 / eV**	**T_1 / eV**	**HOMO / eV**	**LUMO / eV**
**DMAC-TRZ**	2.57	2.56	-5.13	-1.91
**CNPh-**	2.77	2.76	-5.57	-2.17
**CF_3Ph-**	2.67	2.66	-5.38	-2.08
**dPh-**	2.48	2.47	-5.05	-1.97
**^{t}BuPh-**	2.37	2.36	-4.97	-1.93
**OMePh-**	2.43	2.43	-4.87	-1.91

Optoelectronic characterization

Table **S4**. Electrochemical properties of **DMAC-TRZ** and derivatives.

**R-DMAC-TRZ**	**E_{ox} / V[a]**	**E_{red} / V[a]**	**HOMO / eV[b]**	**LUMO / eV[b]**
**DMAC-TRZ**	0.97	-1.72	-5.31	-2.62
**CNPh-**	1.04	-1.68	-5.38	-2.66
**CF_3Ph-**	0.80	-1.68	-5.35	-2.66
**dPh-**	0.86	-1.69	-5.21	-2.65
**^{t}BuPh-**	0.81	-1.71	-5.15	-2.63
**OMePh-**	0.79	-1.69	-5.13	-2.65

[a] obtained from DPV; [b] obtained from the redox potentials from the DPV, $E_{HOMO/LUMO}$=-($E_{ox/red}$+4.8), where $E_{ox/red}$ was obtained from the DPV corrected vs Fc/Fc^+.

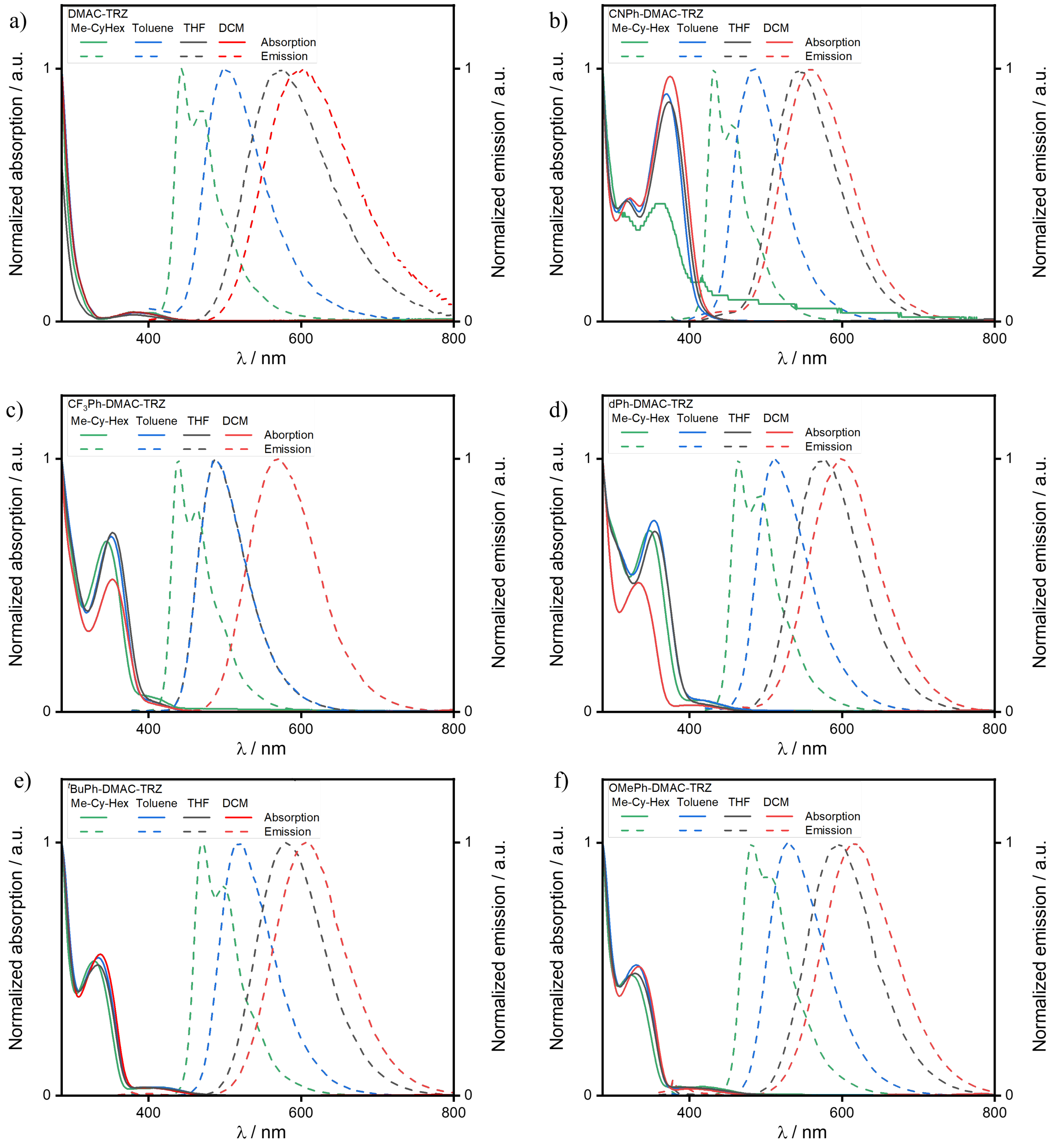

Figure **S36**. Absorption and emission spectra of **DMAC-TRZ** and derivatives in different solvents (λ_{exc}= 340 nm).

Table S5. Emission wavelength of **DMAC-TRZ** and derivatives in different solvents.

**R-DMAC-TRZ**	**λ_{PL} / nm Me-Cyhex**	**λ_{PL} / nm Toluene**	**λ_{PL} / nm THF**	**λ_{PL} / nm DCM**
**DMAC-TRZ**	443/ 470[a]	500	573	602
**CNPh-**	433/ 457[a]	484	544	561
**CF_3Ph-**	439/ 498[a]	487	560	567
**dPh-**	465/ 491[a]	511	576	598
**^{t}BuPh-**	470/ 498[a]	518	580	604
**OMePh-**	482/ 504[a]	528	594	620

[a] peak maxima of the two bands in the Me-Cyhex solution.

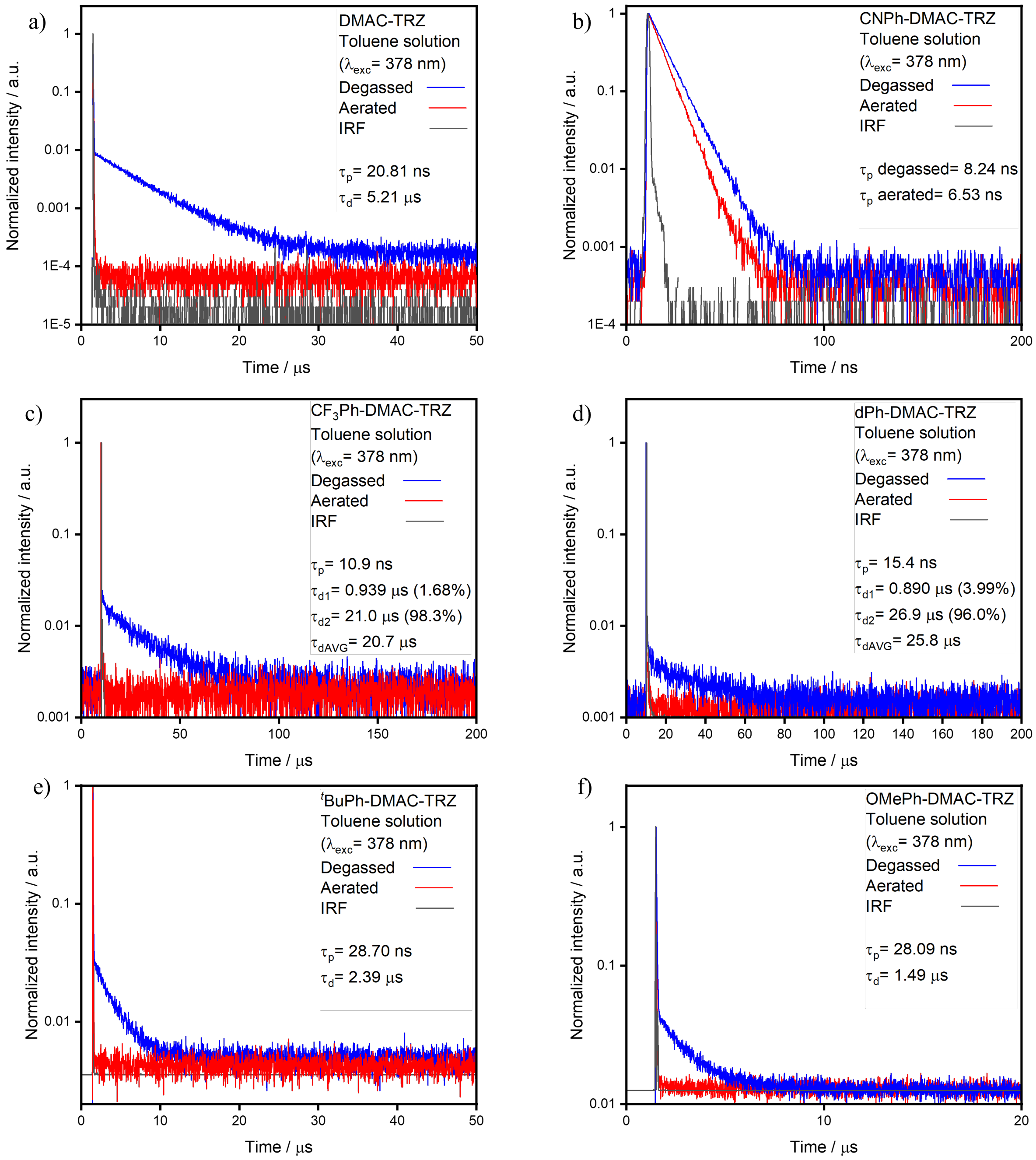

Figure **S37**. Time-resolved fluorescence decay of degassed and aerated solutions of **DMAC-TRZ** and derivatives (Toluene, 10^{-5} M solutions, λ_{exc}= 378 nm).

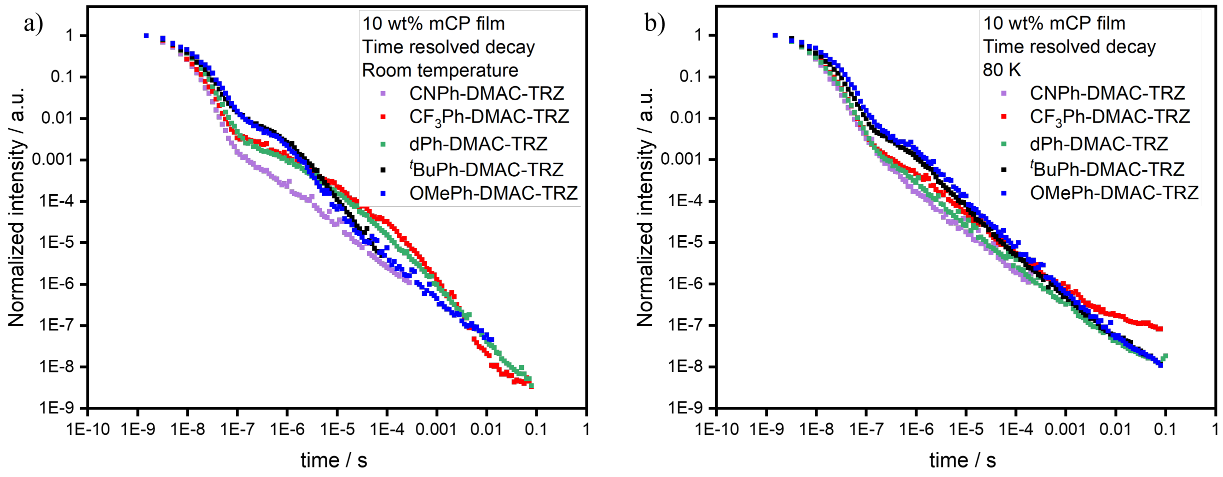

Figure **S38**. Time-resolved fluorescence decay at a) room temperature; b) 80 K of the extended derivatives in 10 wt% doped films in mCP (λ_{exc}= 355 nm).

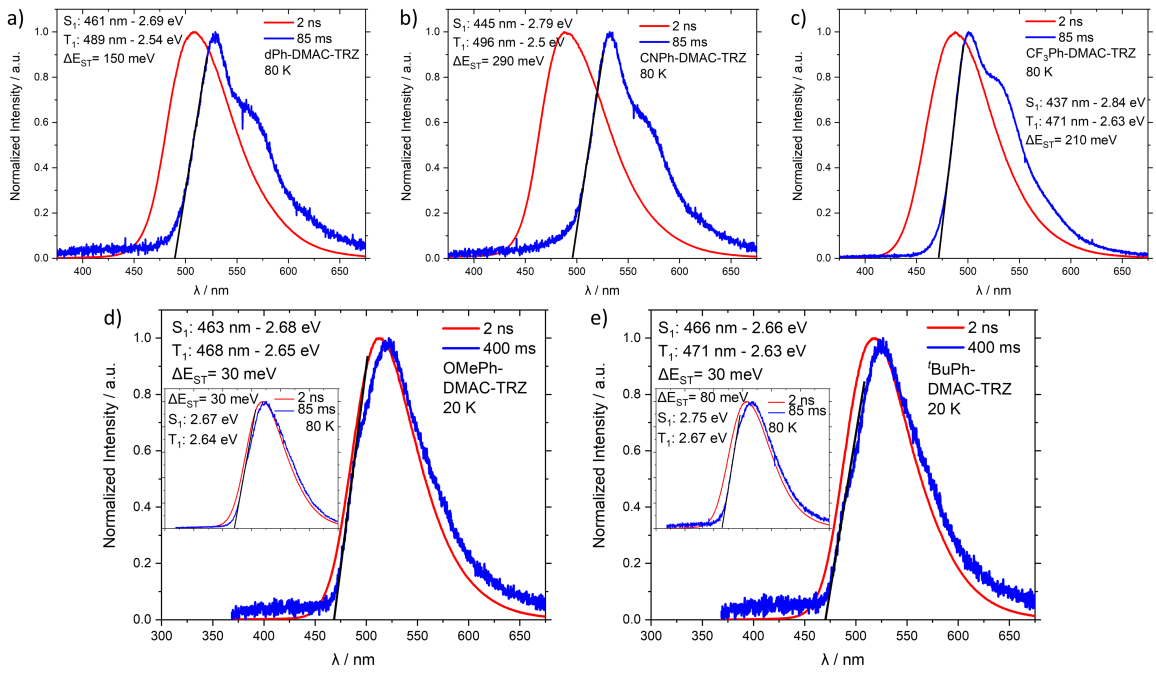

Figure **S39**. Prompt fluorescence (time delay 2 ns) and phosphorescence (time delay 85 ms) spectra of the extended derivatives, in 10 wt% doped films in mCP, at 80 K (a-c). d) OMePh-DMAC-TRZ and e) tBuPh-DMAC-TRZ measured at 20 K because of their small ΔE_{ST}. Inset: the 80 K spectra.

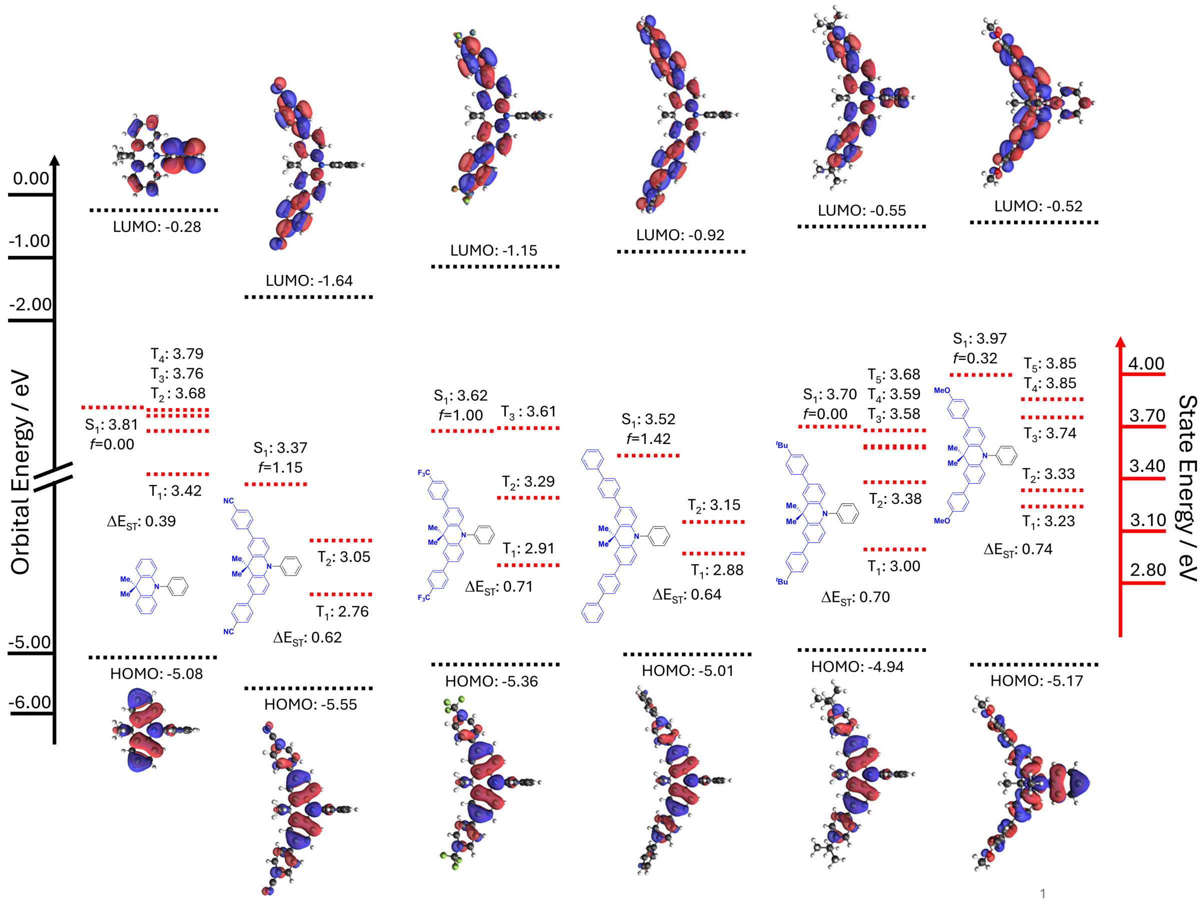

Figure **S40**. HOMO and LUMO electron density distributions and energy levels, excited-state energy levels of DMAC and its five aryl decorated derivatives (Obtained via DFT and TD-DFT at the PBE0/6-31G(d,p) level, Isovalues: MO=0.02).

Table **S6.** Photophysical properties of **DMAC-TRZ** and extended derivatives in toluene solution (10^{-5} M), 10 wt% doped films in mCP and 10 wt% doped films in mCBPCN.

R-DMAC-TRZ	Environment	λ_{PL} / nm[a]	PLQY N_2; Air[b]/ %
**H-**	Toluene (10^{-5} M)	500	67; 21[c]
	mCP 10 wt%	499	47; 45[d]
	mCBPCN 10 wt%	503	87; 62[d]
**CNPh-**	Toluene (10^{-5} M)	481	41; 32[c]
	mCP 10 wt%	499	44; 19[d]
	mCBPCN 10 wt%	498	34; 19[d]

**CF_3Ph-**	Toluene (10^{-5} M)	532	72; 28[c]
	mCP 10 wt%	498	51; 38[d]
	mCBPCN 10 wt%	500	70; 44[d]
**dPh-**	Toluene (10^{-5} M)	514	70; 35[c]
	mCP 10 wt%	516	66; 50[d]
	mCBPCN 10 wt%	527	70; 43[d]
**^{t}BuPh-**	Toluene (10^{-5} M)	524	74; 24[c]
	mCP 10 wt%	515	66; 64[d]
	mCBPCN 10 wt%	531	72; 61[d]
**OMePh-**	Toluene (10^{-5} M)	539	75; 23[c]
	mCP 10 wt%	529	55; 51[d]
	mCBPCN 10 wt%	539	72; 56[d]

[a] measured at room temperature; [b] λ_{exc} = 340 nm; [c] obtained *via* the optically dilute method,[3] quinine sulfate (0.5 M) in H_2SO_4 (aq) was used as the reference, Φ_{PL}: 54.6%, λ_{exc}= 360 nm;[22] [d] obtained *via* integrating sphere, for this method we need to consider an average error of ± 5%.

Table **S7**. Host and concentration screen of **^{t}BuPh-DMAC-TRZ** in mCP, mCBPCN and DPEPO.

**Host**	**Doping concentration / wt%**	**PLQY N_2; Air/ %**
**mCP**	3	65; 61
	5	66; 63
	10	66; 64
	15	62; 60
	20	62; 61
**mCBPCN**[a]	3	62; 57
	5	65; 60
	10	72; 61
	15	79; 64
	20	70; 60
**DPEPO**[b]	3	63; 51

5	67; 49
10	75; 50
15	80; 54
20	73; 55

[a] λ_{exc}=340 nm; [b] λ_{exc}=310 nm

Table **S8**. Emitter properties that might influence the orientation in evaporated films.

**10 wt% emitter in mCBPCN host**	***a***[a]	**MW (g/mol)**	x_E/x_H	z_E **(Å)**
**CNPh-DMAC-TRZ**	0.16	718. 84	1.08	11.53
**CF_3Ph-DMAC-TRZ**	0.26	804.82	1.09	11.53
**dPh-DMAC-TRZ**	0.15	821.02	1.35	11.53
**^{t}BuPh-DMAC-TRZ**	0.25	780.04	1.21	11.53
**OMe-DMAC-TRZ**	0.20	728.88	1.10	11.53
**DMAC-TRZ**	0.21	516.63	0.91	11.53

[a]anisotropy factor.

OLEDs

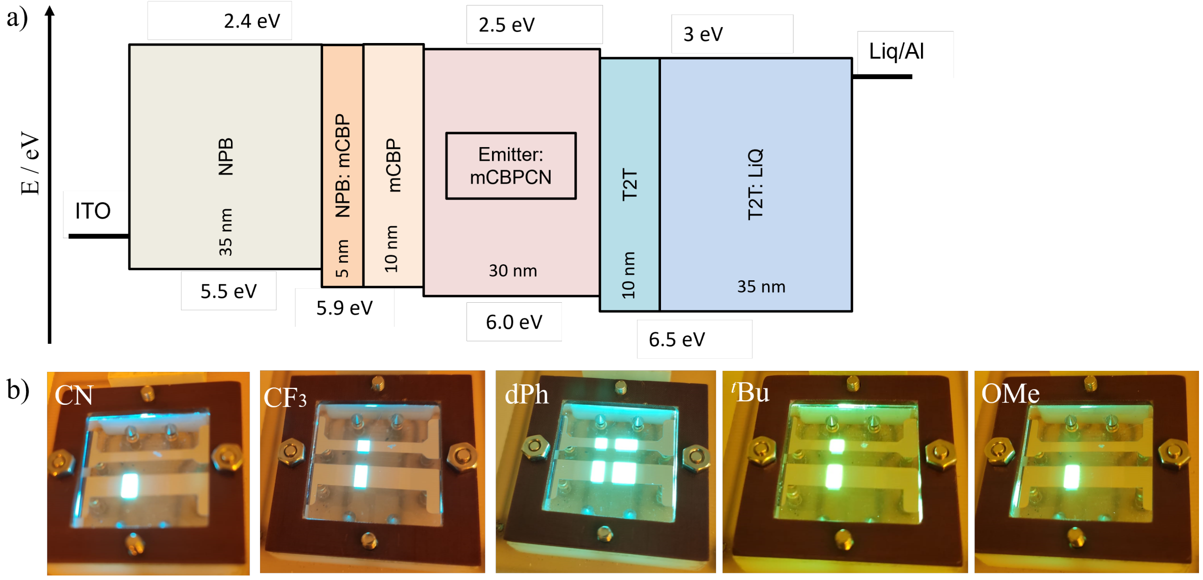

Figure **S41**. **a)** OLED stack; **b)** pictures of the operating OLEDs.

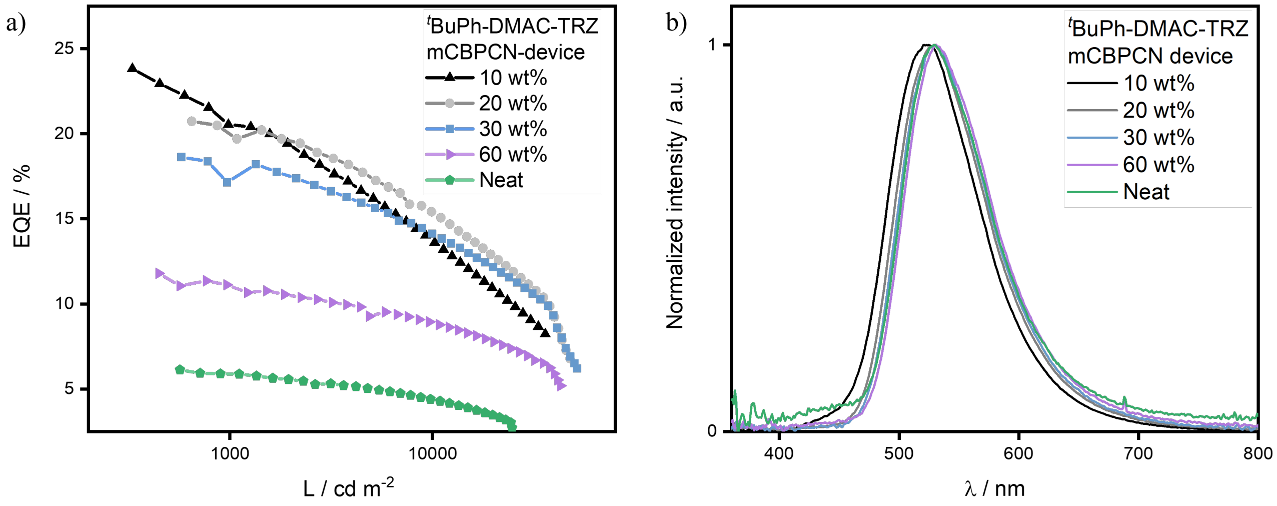

Figure **S42**. a) EQE vs Luminance curves taken above 500 cd m^{-2} and b) Electroluminescence spectra (at 1000 cd m^{-2}) of the ***t*****BuPh-DMAC-TRZ** OLEDs doped at different concentrations.

Light outcoupling efficiency simulations

The light outcoupling for the device stack reported in the manuscript has been simulated using the commercial software SETFOS from Fluxim.[23] A mode analysis for the six OLEDs (with their individual PL emission spectra considered) was performed while sweeping the transition dipole orientation *a* from horizontal (*a*=0 to vertical *a*=1). The experimentally measured orientation is marked by a black square for each emitter and is given together with the simulated light outcoupling in the table below.

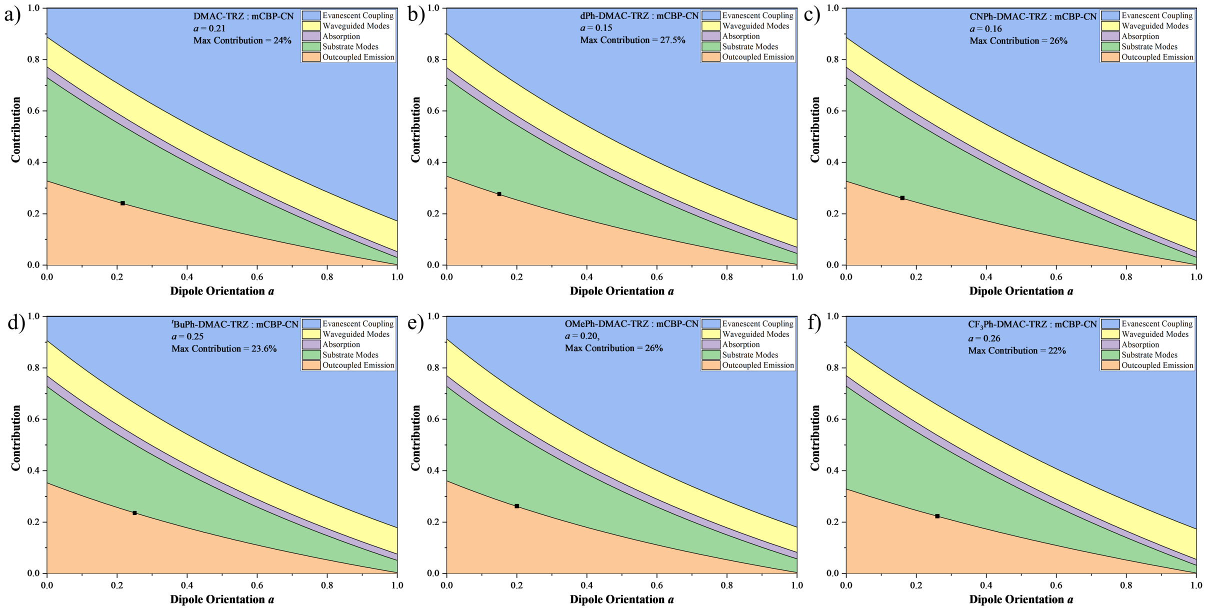

Figure **S43**. OLED light outcoupling simulation. Emitters at 10 wt% concentration in mCBPCN host of a) **DMAC-TRZ,** b) **dPh-DMAC-TRZ**, c) **CNPh-DMAC-TRZ**, d) ***t*BuPh-DMAC-TRZ**, e) **OMePh-DMAC-TRZ**, and f) **CF_3Ph-DMAC-TRZ**.

Table **S9**. Simulated light outcoupling efficiency.

10 wt% emitter in mCBPCN host	*a*	*Outcoupling Efficiency*
**dPh-DMAC-TRZ**	0.15	28%
**CNPh-DMAC-TRZ**	0.16	26%
**OMe-DMAC-TRZ**	0.20	26%
**DMAC-TRZ**	0.21	24%
***t*BuPh-DMAC-TRZ**	0.25	24%
**CF_3Ph-DMAC-TRZ**	0.26	22%

References

(1) Furue, R.; Nishimoto, T.; Park, I. S.; Lee, J.; Yasuda, T. Aggregation-Induced Delayed Fluorescence Based on Donor/Acceptor-Tethered Janus Carborane Triads: Unique Photophysical Properties of Nondoped OLEDs. *Angew. Chemie - Int. Ed.* **2016**, *55* (25), 7171–7175. https://doi.org/10.1002/anie.201603232.

(2) Wu, Z. C.; Boger, D. L. Synthesis, Characterization, and Cycloaddition Reactivity of a Monocyclic Aromatic 1,2,3,5-Tetrazine. *J. Am. Chem. Soc.* **2019**, *141* (41), 16388–16397. https://doi.org/10.1021/jacs.9b07744.

(3) Tsai, W. L.; Huang, M. H.; Lee, W. K.; Hsu, Y. J.; Pan, K. C.; Huang, Y. H.; Ting, H. C.; Sarma, M.; Ho, Y. Y.; Hu, H. C.; Chen, C. C.; Lee, M. T.; Wong, K. T.; Wu, C. C. A Versatile Thermally Activated Delayed Fluorescence Emitter for Both Highly Efficient Doped and Non-Doped Organic Light Emitting Devices. *Chem. Commun.* **2015**, *51* (71), 13662–13665. https://doi.org/10.1039/c5cc05022g.

(4) Hsieh, Y. Y.; Sánchez, R. S.; Raffy, G.; Shyue, J. J.; Hirsch, L.; Del Guerzo, A.; Wong, K. T.; Bassani, D. M. Supramolecular Gating of TADF Process in Self-Assembled Nano-Spheres for High-Resolution OLED Applications. *Chem. Commun.* **2022**, *58* (8), 1163–1166. https://doi.org/10.1039/d1cc06120h.

(5) Song, F.; Xu, Z.; Zhang, Q.; Zhao, Z.; Zhang, H.; Zhao, W.; Qiu, Z.; Qi, C.; Zhang, H.; Sung, H. H. Y.; Williams, I. D.; Lam, J. W. Y.; Zhao, Z.; Qin, A.; Ma, D.; Tang, B. Z. Highly Efficient Circularly Polarized Electroluminescence from Aggregation-Induced Emission Luminogens with Amplified Chirality and Delayed Fluorescence. *Adv. Funct. Mater.* **2018**, *28* (17), 1800051. https://doi.org/10.1002/adfm.201800051.

(6) Ruhstaller, B.; Flatz, T.; Rezzonico, D.; Moos, M.; Reinke, N.; Huber, E.; Haeusermann, R.; Perucco, B. Comprehensive Simulation of Light-Emitting and Light-Harvesting Organic Devices. *Org. Light Emit. Mater. Devices XII* **2008**, *7051* (May 2014), 70510J. https://doi.org/10.1117/12.802447.
